# Identification of Candidate Olfactory Genes in the Antennal Transcriptome of the Stink Bug *Halyomorpha halys*

**DOI:** 10.3389/fphys.2020.00876

**Published:** 2020-07-24

**Authors:** Dongdong Sun, Yuan Huang, Zhenjie Qin, Haixia Zhan, Jinping Zhang, Yang Liu, Shiyong Yang

**Affiliations:** ^1^College of Life Sciences, Anhui Normal University, Wuhu, China; ^2^State Key Laboratory for Biology of Plant Diseases and Insect Pests, Institute of Plant Protection, Chinese Academy of Agricultural Sciences, Beijing, China; ^3^MoA-CABI Joint Laboratory for Bio-safety, Institute of Plant Protection, Chinese Academy of Agricultural Sciences, Beijing, China; ^4^Anhui Provincial Key Laboratory for the Conservation and Exploitation of Biology Resources, College of Life Sciences, Anhui Normal University, Wuhu, China

**Keywords:** *Halyomorpha halys*, antennal transcriptome, chemosensory genes, expression patterns, odorant-binding protein, chemosensory protein

## Abstract

The brown marmorated stink bug, *Halyomorpha halys* (Hemiptera: Pentatomidae), is a serious agricultural and urban pest that has become an invasive species in many parts of the world. Olfaction plays an indispensable role in regulating insect behaviors, such as host plant location, partners searching, and avoidance of predators. In this study, we sequenced and analyzed the antennal transcriptomes of both male and female adults of *H. halys* to better understand the olfactory mechanisms in this species. A total of 241 candidate chemosensory genes were identified, including 138 odorant receptors (ORs), 24 ionotropic receptors (IRs), 15 gustatory receptors (GRs), 44 odorant-binding proteins (OBPs), 17 chemosensory proteins (CSPs), and three sensory neuron membrane proteins (SNMPs). The results of semi-quantitative reverse transcription PCR (RT-PCR) assays showed that some *HhalOBP* and *HhalCSP* genes have tissue-specific and sex-biased expression patterns. Our results provide an insight into the molecular mechanisms of the olfactory system in *H. halys* and identify potential novel targets for pest control strategies.

## Introduction

Natural environments are characterized by a wide variety of odors, and insects have developed corresponding olfactory systems that enable them to recognize and interpret the complicated odorant information (Su et al., 2009). The complex chemosensory system enables insects to sense the volatile odors of host plants, conspecific individuals, and natural enemies, which allows them to locate food sources, mating partners, and predators (Field et al., 2000; Hansson, 2002; Bruce et al., 2005; Asahina et al., 2008; Sato and Touhara, 2009). The recognition of odor molecules by insects is a very sophisticated process. The odorants are detected by olfactory receptor neurons (ORNs) distributed in cuticular sensilla, which are mainly housed in the antennae wherein the chemical signals are converted into electrical signals that are further transmitted to the antennal lobe. The electrical signals then activate the central nervous system and ultimately guide the insect to respond accordingly (Hansson, 2002; Anton et al., 2003; Sato and Touhara, 2009; Rospars et al., 2010; Fleischer et al., 2018).

In the process by which chemical signals are converted into electrical signals at the peripheral nerve level, both receptor and non-receptor gene families are involved. The former includes odorant receptors (ORs), ionotropic receptors (IRs), and gustatory receptors (GRs), whereas the latter includes at least odorant-binding proteins (OBPs), chemosensory proteins (CSPs), and sensory neuron membrane proteins (SNMPs) (Dunipace et al., 2001; Dahanukar et al., 2005; Jin et al., 2008; Vogt et al., 2009; Koh et al., 2014; Wicher, 2015; Agnihotri et al., 2016; Sun et al., 2016; Du et al., 2018).

Insect OR is a type of transmembrane receptor that has seven transmembrane domains (TMDs; Clyne et al., 1999; Smart et al., 2008) and is mainly expressed in the dendritic membrane of the ORNs. The ORs form a heterodimer composed of a conserved, non-conventional OR co-receptor (Orco) and a variable, conventional ORx (Benton et al., 2006; Sato et al., 2008; Leal, 2013). IRs were originally discovered by expression of olfactory neurons and belong to the ionotropic glutamate receptor (iGluR) gene family, a highly conserved family of ligand-gated ion channels (Benton et al., 2009; Croset et al., 2010; Rytz et al., 2013). Recent functional studies indicate that IRs have diverse functions in chemical reception and participate in the sensation of odorants, temperature, humidity, and salt (Chen et al., 2015; Zhang et al., 2019). GRs play key roles in the sensing of CO_2_, sugars, bitter compounds, salts, and some gustatory pheromones (Dahanukar et al., 2007; Kwon et al., 2007; Wanner and Robertson, 2008; Kent and Robertson, 2009; Xu et al., 2012; Mang et al., 2016; Fleischer et al., 2018).

In addition to the three types of receptors, there are a variety of non-receptor proteins involved in insects olfactory perception including OBPs, CSPs, and SNMPS. OBPs and CSPs are small hydrophilic proteins that are abundant in the sensillum lymph. OBPs have six conserved cysteines, which are known as their most prominent characteristic, and can bind to the hydrophobic odor molecules and finally transport them through the sensory lymph to the ORNs around the membrane, activating ORs (Brito et al., 2016). CSPs belong to another class of relatively low-molecular-weight hydrophilic proteins, which are ubiquitous in the sensillum lymph (Jin et al., 2005). CSPs also are expressed in non-olfactory tissues (Jin et al., 2006) and are involved in insect growth and development. The SNMPs are homologous to mammalian CD36 proteins family (Rogers et al., 2001; Nichols and Vogt, 2008). Three subgroups of SNMPs (SNMP1, SNMP2, and SNMP3) have been identified in many insects orders (Rogers et al., 2001; Nichols and Vogt, 2008; Vogt et al., 2009; Gu et al., 2013b; Jiang et al., 2016; Yang et al., 2017; Zhang et al., 2020). SNMP1 plays a role in the detection of sex pheromones, but the exact functions of SNMP2 and SNMP3 are presently unknown (Gomez-Diaz et al., 2016).

The brown marmorated stink bug *Halyomorpha halys* (Hemiptera: Pentatomidae) is a major agricultural pest that is native to much of Asia. In the past 20 years, this pest has invaded parts of North America, South America, and Europe, causing great economic losses and degrading the ecosystem dynamics (Acebes-Doria et al., 2018). *H. halys* is a polyphagous pest that can feed on at least 88 host plants including fruits, vegetables, crops, nuts, weeds, and wild plants (Bergmann et al., 2016; Botch and Delfosse, 2018). This pest often invades human dwellings and commercial buildings and secretes volatile compounds that have a detrimental effect on human skin and eyes (Anderson et al., 2012; Shen and Hu, 2017). Two previous studies have reported transcriptome datasets from distinct developmental stages of *H. halys*, but neither of these studies involved the antennal transcriptome, especially chemosensory genes (Ioannidis et al., 2014; Sparks et al., 2014). Paula et al. (2016) identified a total of 30 full-length putative *OBP* genes in the antennal transcriptome of *H. halys* and examined their expression in the presence of food, aggregation pheromone, and alarm pheromone stimuli, but they did not analyze other olfactory genes. In this study, we identified 241 candidate olfactory genes in *H. halys* comprising 138 ORs, 24 IRs, 15 GRs, 44 OBPs, 17 CSPs, and three SNMPs in the adult *H. halys* antennae. The sequence and phylogenetic analyses were performed on these important olfactory genes. Tissue-specific expression patterns of the *OBP* and *CSP* genes were determined using semi-quantitative reverse transcription PCR (RT-PCR). The results of this study not only lay a foundation for the functional characterization of these chemosensory genes but also provide a theoretical basis for the future development of new technology to control *H. halys* using insect-related olfactory genes as targets.

## Materials and Methods

### Insect Rearing and Tissue Collection

Overwintering *H. halys* adults were collected from Baiwang Mountain Forest Park (116°21′43″–116°28′12″E, 39°57′52″–40°02′11″), Beijing, China. Male and female adults were identified by sexing. The antennae were removed with tweezers, flash frozen in liquid nitrogen, and stored at −70°C until use.

### cDNA Library Construction and Sequencing

Total RNA was extracted from one pool each of 30 male antennae and 30 female antennae of *H. halys* using TRIzol reagent, following the manufacturer’s instructions (Invitrogen, Carlsbad, CA, United States). The purity and concentration of the RNA samples were determined using a NanoDrop ND-2000 Spectrophotometer (NanoDrop Technologies, Inc., Wilmington, DE, United States), and RNA integrity was verified by gel electrophoresis. Two micrograms of RNA from each sample was used for cDNA library construction. The construction and sequencing of the two *H. halys* antennae cDNA libraries (male and female) were performed at Beijing Genomics Institute (Shenzhen, China). The insert sequence length was ∼280 bp. The two libraries were pair-end sequenced using PE150 strategy in Illumina HiSeq 4000 platform (Illumina, San Diego, CA, United States).

### Transcriptome Assembly and Functional Annotation

Datasets of clean reads were generated from the raw reads by removing adaptor sequences, poly-*N*-containing reads, and low-quality sequences. The clean reads of the two transcriptomes produced in this study are stored in the National Center for Biotechnology Information (NCBI) Sequence Read Archive (SRA) database under the accession numbers SRR11748354 (female antennae) and SRR11747758 (male antennae). The clean-read datasets of male and female antenna were fed to Trinity (release-20130225) for *de novo* transcriptome assembly using the pair-end reads mode with default parameters (Grabherr et al., 2011). The Trinity outputs were clustered using TGICL V2.1 (Pertea et al., 2003). The consensus cluster sequences and singletons made up the final unigene dataset.

### Identification of Chemosensory Genes

Unigenes were annotated using blastx searches against the NCBI non-redundant (nr) database with an e-value < 1e^–5^. Candidate unigenes encoding putative ORs, IRs, GRs, OBPs, CSPs, and SNMPs were identified based on to the nr annotation results. All candidate chemosensory genes were then manually checked using the blastx program against the nr database. And all predicted olfactory gene sequences possessed overlapping regions with low identity and, therefore, likely represent unigenes.

### Sequence and Phylogenetic Analyses

The open reading frames (ORFs) of all genes were predicted using ExPASy server^1^. Putative N-terminal signal peptides of OBPs and CSPs were predicted by SignalP 4.0^2^ in default parameters (Petersen et al., 2011). The TMDs of ORs, IRs, and GRs were predicted using the TMHMM server version 2.0^3^. Amino acid sequence alignments were performed with MAFFT^4^. Phylogenetic trees of CSPs were constructed by RAxML version 8 using the Jones–Taylor–Thornton (JTT) amino acid substitution model (Stamatakis, 2014), and a bootstrap procedure of 1000 replicates was used to evaluate the node support. The OR phylogenetic tree was constructed using the total of 319 ORs from three Hemiptera species: 138 ORs from *H. halys*, 122 from *Oncopeltus fasciatus*, and 59 from *Tessaratoma papillosa* (Wu et al., 2017; Panfilio et al., 2019). For IRs, a phylogenetic analysis was conducted using a dataset containing all 24 IRs in *H. halys* together with other insects including 39 IRs from *Nilaparvata lugens*, 37 from *O. fasciatus*, 32 from *Rhodnius prolixus* 12 from *T. papillosa*, and 80 from *Drosophila melanogaster* (Benton et al., 2009; Mesquita et al., 2015; Wu et al., 2017; He et al., 2018; Panfilio et al., 2019). For GRs, a phylogenetic analysis that included 15 GRs from *H. halys*, 28 from *N. lugens*, 169 from *O. fasciatus*, and 31 from *R. prolixus* was performed (Mesquita et al., 2015; He et al., 2018; Panfilio et al., 2019). The OBP phylogenetic tree was constructed using 44 OBPs from our *H. halys*, 17 from *R. prolixus*, 33 from *T. papillosa*, five from *Acyrthosiphon pisum*, and 10 from *Cyrtorhinus lividipennis* (De Biasio et al., 2015; Mesquita et al., 2015; Wang G.Y. et al., 2017; Wu et al., 2017). For CSPs, the phylogenetic tree was constructed using 17 CSPs from *H. halys*, nine from *Aphis gossypii*, 11 from *N. lugens*, and eight from *R. prolixus* (Gu et al., 2013a; Mesquita et al., 2015; Xue et al., 2016).

### Semi-Quantitative Reverse Transcription PCR

Reverse transcription PCR was performed to examine the expression of candidate OBP and CSP genes in different tissues and both sexes of *H. halys*. Thirty pairs of antennae, 10 heads (without antennae), five thoraxes, five abdomens, and 36 legs (including the fore, median, and hind legs) were collected from male and female *H. halys* adults. Total RNA was extracted as described above. The cDNA was synthesized from total RNA using the RevertAid First Strand cDNA Synthesis Kit (Thermo Fisher Scientific, Pittsburgh, PA, United States). Gene-specific primers were designed using Primer Premier 5.0 software (PREMIER Biosoft, Palo Alto, CA, United States) and synthesized by Sangon Biotech (Shanghai, China). An actin gene fragment was used as a reference. The sequences of primers are listed in Supplementary Table S1. The RT-PCR assays were conducted with a Veriti Thermal Cycler (Applied Biosystems, Carlsbad, CA, United States) and performed in a 20-μl reaction system, which contained 10 μl of 2 × Taq MasterMix (CWBIO, Beijing, China), 1 μl of each primer (10 μM), 1 μl of cDNA, and 7 μl of deionized water. The PCRs were 94°C for 3 min, followed by 27–35 cycles of 94°C for 30 s, 53–58°C (primer-dependent) for 30 s, and 72°C for 30 s, with a final extension at 72°C for 10 min. The PCR products were separated by electrophoresis on 2% agarose gels, stained by ethidium bromide (EB), and photographed under UV light using a Gel Doc XR + System with Image Lab Software (Bio-Rad, Hercules, CA, United States). The RT-PCR was repeated three times using one group of RNA samples (three technical replicates).

## Results

### Sequencing and Unigene Assembly

The antennal transcriptomes of female and male *H. halys* adults were constructed and sequenced separately on Illumina HiSeq 4000 platform. A total of 80.33 million and 85.57 million raw reads were obtained from the male and female antennae, respectively. After filtering, 74.26 million and 78.97 million clean reads were generated from the male and female *H. halys* antennae transcriptomes, respectively. The *de novo* assemblies led to the generation of 48,875 and 58,935 unigenes from the female antenna and male antenna, respectively. After merging and clustering, a final transcript dataset with 65,914 unigenes was produced. The dataset was 71.5 megabases in size with a mean length of 1085 nt and N50 of 2342 nt (Supplementary Table S2).

### Identification of Candidate Odorant Receptors

Candidate ORs were identified using key word searches of the blastx annotation. We identified 138 putative OR genes in *H. halys*. Of these candidate ORs, 93 unigenes were full-length putative OR genes with complete ORFs and an average length of 1200 bp and five to eight predicted TMDs, which is characteristic of typical insect ORs. The OR co-receptor, named HhalOrco, was also found. Other putative ORs (HhalOR1–HhalOR137) were given names followed by a numeral in descending order of the length of their coding regions. Information including unigene reference, length, and best blastx hit of all putative ORs is listed in Supplementary Table S3. The amino acid sequences of the ORs identified in this study are provided in Supplementary File S1.

**FIGURE 1:**
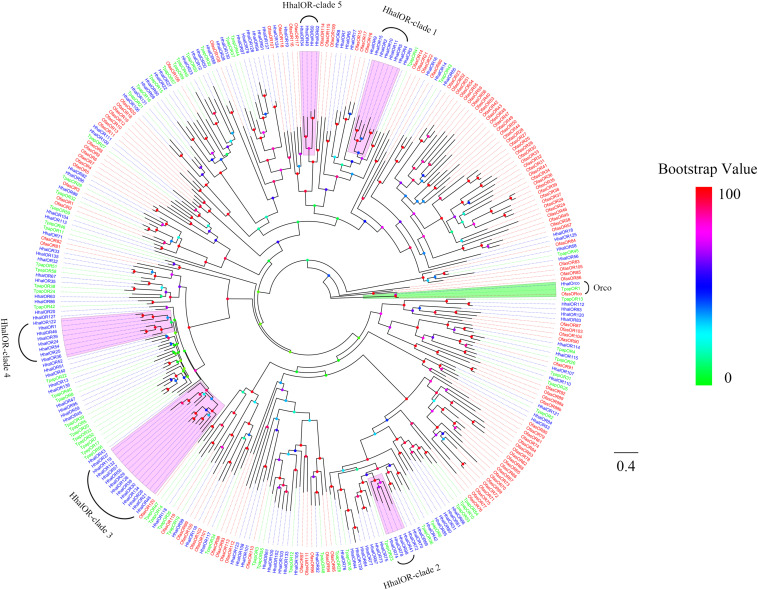
Phylogenetic tree of putative HhalORs with known Hemiptera odorant receptors (ORs). Hhal, *Halyomorpha halys* (blue); Tpap, *Tessaratoma papillosa* (green); Ofas, *Oncopeltus fasciatus* (red). The clade in green indicates the Orco and in lavender the *H. halys* expansions ORs. The scale bar equals 0.4 substitutions per sequence position. The bootstrap values are shown in the circles of different colors.

The phylogeny of *H. halys* ORs is shown in Figure 1. The phylogenetic tree was constructed using the candidate ORs of *H. halys* and other Hemipteran ORs containing *T. papillosa* and *R. prolixus*. As expected, the highly conserved HhalOrco belonged to the same clade as the orthologous proteins from the two other Hemiptera species (Figure 1). Compared with OfasORs, HhalORs were clustered more closely to TpapORs. Compared with the ORs from the other two species, we found five expansions in the number of ORs in *H. halys*, which were marked as HhalOR-clade 1 to HhalOR-clade 5 in Figure 1.

### Identification of Candidate Ionotropic Receptors

The second type of olfactory receptor, IR, belongs to an ancient chemosensory receptor family. In the *H. halys* transcriptome in the present study, 24 putative IRs were identified based on their similarity to known insect IRs. Of these IRs, eight sequences contained full-length ORFs, and the remaining 16 sequences were incomplete owing to lacking a 5′ and/or 3′ terminus. A total of 17 IRs contain more than three TMDs as predicted by TMHMM 2.0 (Supplementary Table S3), which is consistent with the characteristics of insect IRs. The two IR co-receptors HhalIR8a and HhalIR25a were readily detected. Eight HhalIRs (HhalIR21a.1, 21a.2, 60f, 68a, 75d.1, 75d.2, 76b, and 93a) were named based on their orthologous relationships with IRs from *D. melanogaster*. Other putative IRs were named based on their homology from *O. fasciatus* and *R. prolixus* or by a numeral in descending order of the length of their corresponding coding regions. Information including unigene reference, length, and best blastx hit of all putative IRs is listed in Supplementary Table S4. The amino acid sequences of the IRs identified in this study are provided in Supplementary File S1.

**FIGURE 2:**
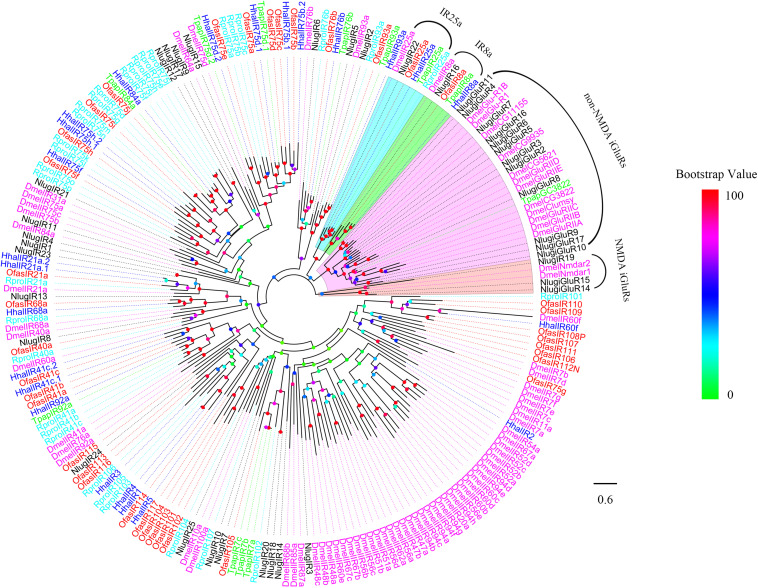
Phylogenetic tree of putative HhalIRs with known Hemiptera ionotropic receptors (IRs). Hhal, *Halyomorpha halys* (blue); Tpap, *Tessaratoma papillosa* (green); Ofas, *Oncopeltus fasciatus* (red); Rpro, *Rhodnius prolixus* (cyan); Dmel, *Drosophila melanogaster*; Nlug, *Nilaparvata lugens* (black). The clade in violet indicates the non-NMDA ionotropic glutamate receptors, in orange indicates NMDA ionotropic glutamate receptors, in cyan indicates the IR25a, and in green indicates the IR8a. The scale bar equals 0.6 substitutions per sequence position. The bootstrap values are shown in the circles of different colors.

We performed a phylogenetic analysis using the candidate HhalIRs and IRs of *T. papillosa*, *O. fasciatus*, *R. prolixus*, *N. lugens*, and *D. melanogaster* (Figure 2). Obviously, the co-receptors Hhal8a and IR25a clustered to form the IR8a and IR25a evolutionary clades, respectively. The remaining IRs clustered on different branches of other species, and almost all IRs are convergent to *T. papillosa* and *O. fasciatus*. Significant separation of iGluRs from IRs was found in the phylogenetic tree. A unique HhalIR2 gene that has no homologous gene in other close species was found (Figure 2).

### Identification of Candidate Gustatory Receptors

Fifteen putative GRs were identified in the *H. halys* transcriptome. Four GR sequences contained a full-length ORF; the remaining 11 sequences were incomplete owing to the absence of the 5′ and/or 3′ terminus (Supplementary Table S5). All putative GRs (HhalGR1–HhalGR15) were given a name followed by a numeral in descending order of the length of their coding regions. Information including unigene reference, length, and best blastx hit of all putative GRs is listed in Supplementary Table S5. The amino acid sequences of the GRs identified in this study are provided in Supplementary File S1.

**FIGURE 3:**
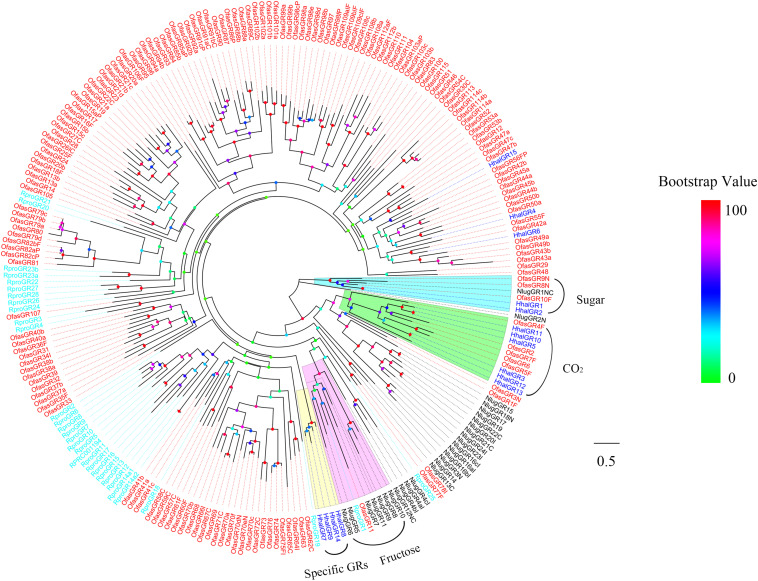
Phylogenetic tree of putative HhalGRs with known Hemiptera gustatory receptors (GRs). Hhal, *Halyomorpha halys* (blue); Ofas, *Oncopeltus fasciatus* (red); Rpro, *Rhodnius prolixus* (cyan); Nlug, *Nilaparvata lugens* (black). The clade in cyan indicates the sugar receptors, in green the CO_2_ receptors, in violet the fructose receptors, and in yellow the *H. halys* specific GRs. The scale bar equals 0.5 substitutions per sequence position. The bootstrap values are shown in the circles of different colors.

To identify GRs in *H. halys*, the putative proteins were analyzed phylogenetically against known Hemipteran GRs including sugar receptor and CO_2_ receptor from *O. fasciatus*, *R. prolixus*, and *N. lugens* (Figure 3). The phylogenetic analysis showed that HhalGR1 and HhalGR2 are in a clade with the sugar receptors, whereas the highly similar HhalGR3, HhalGR5, and HhalGR10-13 were members of the candidate CO_2_ receptor. There was no GR in *H. halys* that clustered with the fructose lineage. HhalGR6 clustered with OfasGR42a; and HhalGR15 clustered together with OfasGR56; and both clusters had high bootstrap support. We identified a species-specific branch that included four HhalGRs: HhalGR7–9 and HhalGR14.

### Identification of Candidate Odorant-Binding Proteins

Forty-four putative unigenes encoding OBPs were identified from the *H. halys* transcriptome. Among them, 37 were full-length putative OBP genes, and the remaining seven were incomplete owing to the absence of the 5′ or 3′ terminus (Supplementary Table S6). All the 30 OBPs (HhalOBP1–30) identified in the previous study were found in our transcriptome, and we also found an additional 14 new OBPs (HhalOBP31–HhalOBP44), which were named using the same convention for the ORs, IRs, and GRs. Information including unigene reference, length, and best blastx hit for all putative OBPs is listed in Supplementary Table S6. The amino acid sequences of the OBPs identified in this study are provided in Supplementary File S1.

**FIGURE 4:**
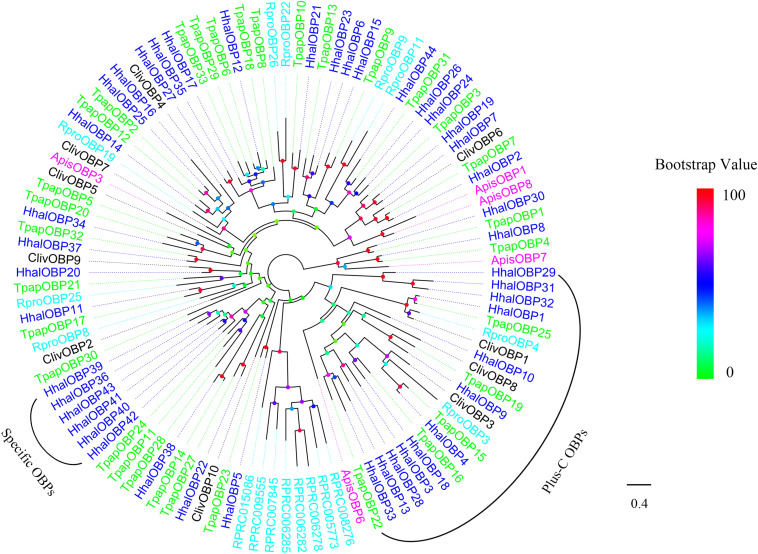
Phylogenetic tree of putative HhalOBPs with known Hemiptera odorant-binding proteins (OBPs). Hhal, *Halyomorpha halys* (blue); Tpap, *Tessaratoma papillosa* (green); Rpro, *Rhodnius prolixus* (cyan); Apis, *Acyrthosiphon pisum* (violet); Cliv, *Cyrtorhinus lividipennis* (black). The scale bar equals 0.4 substitutions per sequence position. The bootstrap values are shown in the circles of different colors.

The phylogenetic tree of HhalOBPs was constructed using the candidate HhalOBPs and OBPs from other Hemipteran species including *A. pisum*, *R. prolixus*, *T. papillosa*, and *C. lividipennis* (Figure 4). Based on the number of conserved cysteines of each OBP, 12 HhalOBPs (HhalOBP1, 3, 4, 9, 10, 13, 18, 28, 29, 31, 32, and 33) were classified as Plus-C OBPs (Supplementary Figure S1), and the remaining 32 OBPs are classified as classic OBPs (Supplementary Figure S1). Minus-C OBP was not found in *H. halys*. Moreover, six HhalOBPs (HhalOBP36, 39, 40, 41, 42, and 43) were clustered into the same clade, which may be related to their unique functions.

### Identification of Candidate Chemosensory Proteins

Seventeen putative unigenes encoding CSPs were identified in the antennal transcriptome of *H. halys*. Among these unigenes, 15 sequences were predicted to encode full-length putative CSP proteins because they had complete ORFs and four cysteines, a characteristic of typical insect CSPs. The remaining two sequences were incomplete owing to the lack of 5′ or 3′ terminus (Supplementary Table S7). All putative CSPs (HhalCSP1–HhalCSP17) were named as described above. The information including unigene reference, length, and best blastx hit of all putative CSPs is listed in Supplementary Table S7. All of the identified amino acid sequences have the highly conserved four-cysteine profiles (Supplementary Figure S2). The amino acid sequences of the CSPs identified in this study are provided in Supplementary File S1.

**FIGURE 5:**
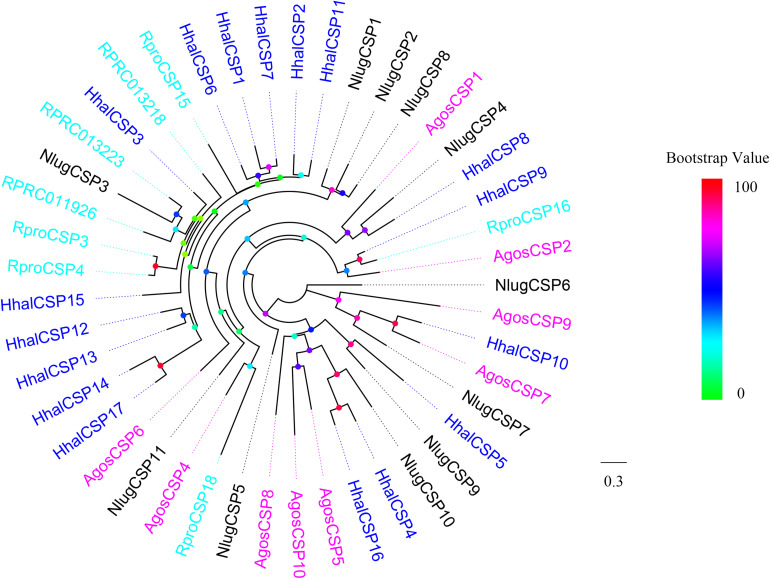
Phylogenetic tree of putative HhalCSPs with known Hemiptera chemosensory proteins (CSPs). Hhal, *Halyomorpha halys* (blue); Agos, *Aphis gossypii* (violet); Rpro, *Rhodnius prolixus* (cyan); Nlug, *Nilaparvata lugens* (black). The scale bar equals 0.3 substitutions per sequence position. The bootstrap values are shown in the circles of different colors.

A phylogenetic tree was built using all of the putative *H. halys* CSPs and those from *A. gossypii*, *R. prolixus*, and *N. lugens* (Figure 5). HhalCSP5 clustered with NlugCSP9, HhalCSP9 clustered with RproCSP16, and HhalCSP10 clustered with AgosCSP7. The remaining HhalCSPs did not cluster with CSPs from other Hemiptera species.

### Identification of Candidate Sensory Neuron Membrane Proteins

Three unigenes encoding SNMPs (HhalSNMP1.1, HhalSNMP1.2, and HhalSNMP2) were identified in the *H. halys* transcriptome. Two unigenes were predicted to encode full-length SNMP proteins as they contain complete ORFs that are > 2000 bp long. HhalSNMP1.2 was incomplete owing to a lack of the 5′ terminus. Two HhalSNMP1s (HhalSNMP1.1 and HhalSNMP1.2) were found in our transcriptome with the supporting of blastx results. Information including unigene reference, length, and best blastx hit of all putative SNMPs is listed in Supplementary Table S8. The amino acid sequences of the SNMPs identified in this study are provided in Supplementary File S1.

### Tissue- and Sex-Specific Expression of Candidate *OBP* and *CSP* Genes in *Halyomorpha halys*

**FIGURE 6:**
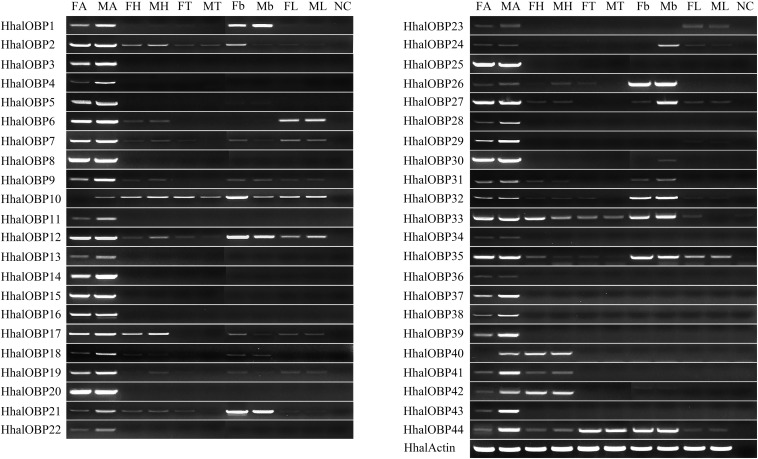
The expression levels of *HhalOBP* genes in different tissues of *Halyomorpha halys* as estimated by RT-PCR. FA, female antennae; MA, male antennae; FH, female head; MH, male head; FT, female thorax; MT, male thorax; Fb, female belly; Mb, male belly; FL, female legs; ML, male legs; NC, no template control. HhalActin was used as a reference gene.

In order to better understand the role of OBPs and CSPs in the olfactory system in *H. halys*, we studied the expression patterns of the 44 *HhalOBP* and 17 *HhalCSP* genes in antennae, head (without antennae), thorax, abdomen, and legs in adults of both sexes using the semi-quantitative RT-PCR. The results showed that 20 of the 44 *HhalOBP* genes (including OBP3–5, 8, 11, 13–16, 20, 22, 25, 28, 29, 34, 36–39, and 43) are specifically expressed in male and female antenna (Figure 6). We also found that 13 *HhalOBP* genes (OBP1, 2, 6, 7, 9, 12, 17, 19, 27, 30, 33, 35, and 40) are highly expressed in the antennae, but they are also weakly or highly expressed in other tissues (Figure 6). Four *HhalOBP* genes (OBP10, 21, 26, and 32) are poorly expressed in the antennae but were highly expressed in belly tissues of both sexes (Figure 6). Furthermore, *HhalOBP10* and *HhalOBP40* are only expressed in the male antennae, and no expression is found in the female antennae (Figure 6).

**FIGURE 7:**
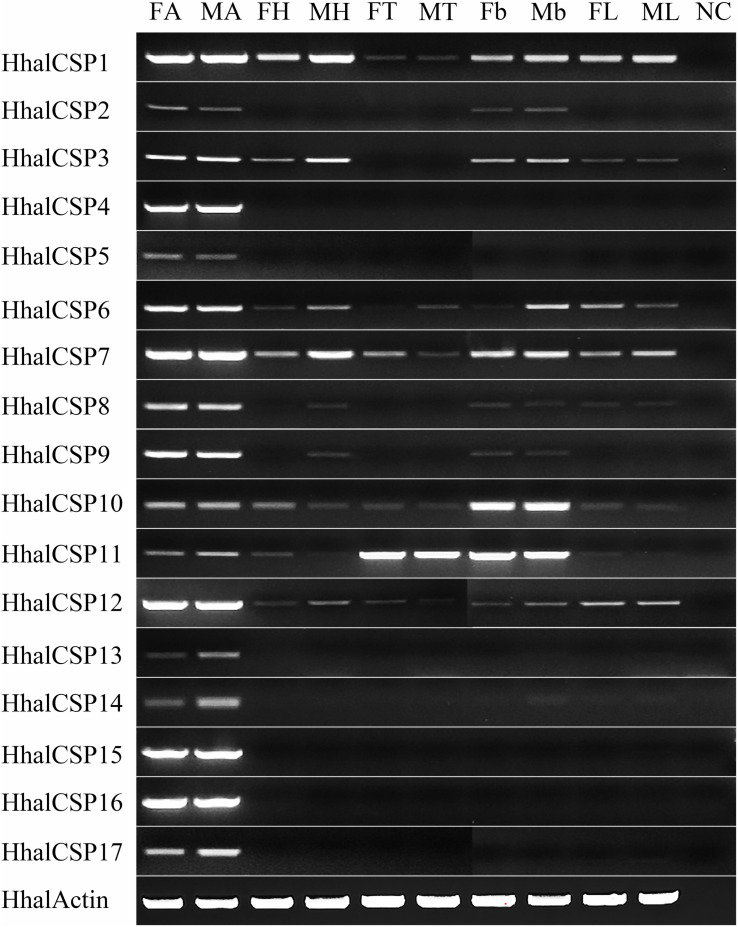
The expression levels of *HhalCSP* genes in different tissues of *Halyomorpha halys* as estimated by RT-PCR. FA, female antennae; MA, male antennae; FH, female head; MH, male head; FT, female thorax; MT, male thorax; Fb, female belly; Mb, male belly; FL, female legs; ML, male legs; NC, no template control. HhalActin was used as a reference gene.

As shown in Figure 7, all of the *HhalCSP* genes are expressed in the antennae of male and female *H. halys*; *HhalCSP1*, *HhalCSP6–7*, and *HhalCSP10–12* are expressed in all of these tested tissues; *HhalCSP4–5* and *HhalCSP13–17* are only expressed in the male and female antennae, not in any other tissues; *HhalCSP2* is expressed in the abdomen of both sexes in addition to the antennae; and *HhalCSP3* is expressed in all of the tested tissues of both sexes except the thorax. HhalCSP10 is highly expressed in the abdomen of both sexes, and HhalCSP11 is highly expressed in the thorax and abdomen of both sexes of *H. halys* (Figure 7).

## Discussion

*H. halys* is an important agricultural pest of global concern. This invasive pest is native to Asia, but it has been introduced into many countries in Europe, the Americas, and Oceania, where it causes enormous economic losses and is a nuisance to humans, especially during overwintering (Lee et al., 2013). Recent decades have witnessed the rapid development of insect antennal transcriptome studies, but only a few studies have focused on the Hemiptera, especially the Pentatomidae family (stink bugs). Investigations into the mechanisms of olfaction in *H. halys* will be useful for functional characterization of olfaction genes and could ultimately lead to the identification of new targets for olfactory disruption and development of environmentally friendly pest control strategies. Previously, Paula et al. (2016) identified 30 OBP from *H. halys* antennae, but they did not examine other olfactory genes. In this study, we reported the sequencing, assembly, and annotation of antennal transcriptomes in *H. halys*; and we identified 138 ORs, 24 IRs, 15 GRs, 44 OBPs, 17 CSPs, and three SNMPs. We also assayed the expression profiles of 44 *OBP* and 17 *CSP* genes in different tissues of *H. halys* using RT-PCR.

Odorant receptors are crucial to the insect olfactory system, as they determine the sensitivity and specificity of odorant reception (Wang B. et al., 2017). In this study, we identified a total of 138 OR genes in *H. halys* antennal transcriptomes, which was more than those in *O. fasciatus* (122) and in *T. papillosa* (59). These differences in the numbers of identified OR genes could be attributed to the differences in sequencing methods and depth or sample preparation between this study and the other studies. The number of olfactory genes identified here may not reflect all of the olfactory genes in *H. halys*, because some olfactory genes are expressed in tissues other than antenna. Orco is a highly conserved olfactory co-receptor that plays important roles in insect olfaction and does not function directly in odor recognition but rather forms the obligate co-receptor for all ORs (Larsson et al., 2004; Vosshall and Hansson, 2011). The results of the phylogenetic analysis showed that HhalORs are more closely related to TpapORs than they are to OfasORs, which is consistent with the evolutionary relationship among these three species: *H. halys*, *T. papillosa*, and *O. fasciatus* (Yuan et al., 2015). Although the sequences of insect ORs are highly diverse, we found some ORs from *H. halys* and *T. papillosa* that have high sequence similarities. HhalOR45/TpapOR58 and HhalOR89/TpapOR32 share 80.24 and 82.16% sequence similarity, respectively, suggesting that they have some common and possibly identical olfactory functions.

A total of 24 IRs were identified in the antennal transcriptomes of *H. halys*. This is considerably lower than those numbers found in *N. lugens* (39), *O. fasciatus* (37), and *R. prolixus* (32), but more than those in *T. papillosa* (12). It is possible that some IR genes are not expressed in antennae or, alternatively, that the number of IRs is species specific and is dependent on natural habitats. Similar to Orco, both IR8a and IR25a are predicted to act as co-receptors present in the IR group because they were co-expressed along with other IRs (Benton et al., 2009; Sheng et al., 2017; Du et al., 2018). TheiGluRs are a highly conserved family of ligand-gated ion channels that mediate chemical communication between neurons at synapses (Croset et al., 2010; Abuin et al., 2011). And in the phylogenetic tree, IR8a and IR25a formed two conserved IR clades.

Members of the GR family of insect chemoreceptors are diverse and demonstrate a broad ligand selectivity for several molecules including receptors for sugars, deterrents, salts, fructose, bitter compounds, CO_2_, and other compounds (Xu et al., 2012; Agnihotri et al., 2016; Fleischer et al., 2018). This type of receptor plays a critical role in chemo-sensation and influences the insect’s behavior. However, the functions of GRs are largely unknown. In the present study, we identified 15 GRs in the *H. halys* antennal transcriptome. The number of predicted GRs in *H. halys* is less than that in *N. lugens* (28) and *R. prolixus* (31) and far fewer than the number in *O. fasciatus* (122). A phylogenetic analysis indicated that HhalGR3, HhalGR5, and HhalGR10–13 may play a role in CO_2_ detection. HhalGR1 and HhalGR2 are predicted to be candidate sugar receptors that are related to host plant selection and oviposition (Lombarkia and Derridj, 2008; Wang J. et al., 2017).

Odorant-binding proteins are mainly present in insect sensillum lymph, and they can package odor molecules and finally transport them by the sensory lymph to the ORNs around the membrane, activating ORs or IRs (Brito et al., 2016). Among the 44 putative OBPs identified in the present study, 12 HhalOBPs (HhalOBP1, 3, 4, 9, 10, 13, 18, 28, 29, and 31–33) were clustered with the Plus-C OBPs from other insect species. The remaining 32 HhalOBPs were clustered with the classic OBPs, which are characterized by a conserved six-cysteine-residue pattern. In contrast, Paula et al. (2016) identified 30 HhalOBPs from the antennae of 3-day-old *H. halys* males and females, including 22 classic OBPs and eight plus-C ones. A possible explanation is that these two studies used different sequencing platforms; that is, HiSeq 4000 platform was used in our study, whereas Illumina HiSeq 2500 was used by Paula et al. (2016). The skills for *de novo* transcriptome assembly and annotation can also result in different numbers of OBPs. Of the 44 OBPs, 20 candidate *HhalOBP* genes seem to be expressed only in the male and female antennae, suggesting their roles in host plant location. Six HhalOBPs (HhalOBP36 and 39–43) were clustered in one branch, and these OBPs may have similar functions in *H. halys*. In addition, *HhalOBP10* and *HhalOBP40* were only found in male antenna, indicating their potential function in finding mates. Thirteen *HhalOBP* genes were highly expressed in the antennae and were also expressed in other tissues, indicating their involvement in the binding of non-odorant molecules in addition to the recognition process of odorants. HhalOBPs may have functions to bind *H. halys* pheromones. The expression of 21 HhalOBPs was enhanced in response to alarm pheromone and two HhalOBPs (HhalOBP4 and HhalOBP8) to aggregation pheromone (Paula et al., 2016). We found that HhalOBP8, 16, 25, and 30 were highly expressed in *H. halys* antennae. Coincidently, these four HhalOBPs had higher binding activities to the major component of the alarm pheromone, (*E*)-2-decenal, of *H. halys* (Zhong et al., 2018). The epoxides (3*S*,6*S*,7*R*,10*S*)-10,11-epoxy-1-bisabolen-3-ol and (3*R*,6*S*,7*R*,10*S*)-10,11-epoxy-1-bisabolen-3-ol were identified as the main components of the aggregation pheromone of *H. halys* (Khrimian et al., 2014; Weber et al., 2014). HhalOBP4 and HhalOBP8 may be the proteins that bind the aggregation pheromone of *H. halys*. However, these assumptions remain to be tested. Our findings, together with those of Paula et al. (2016) and Zhong et al. (2017, 2018), would provide a basis to understand the physiological functions of HhalOBPs and may facilitate to develop approaches for behavioral interference of the pest.

Chemosensory proteins are a class of low-molecular-weight proteins widely found in sensillum lymph, and all of them have a highly conserved four-cysteine profile (Jin et al., 2005). We identified 17 CSPs in the *H. halys* antennal transcriptome. The number of HhalCSPs is higher than in other Hemiptera species, such as *A. gossypii* (nine CSPs), *N. lugens* (11 CSPs), and *R. prolixus* (eight CSPs). The RT-PCR results showed that *HhalCSP4–5*, *HhalCSP8–9*, and *HhalCSP13–17* are enriched in the antennae and may be involved in the chemosensory process (Zhang et al., 2014).

There are three SNMPs (SNMP1, SNMP2, and SNMP3) subfamilies identified in insects (Vogt et al., 2009; Zhang et al., 2020). In our study, we found three SNMPs (SNMP1.1, SNMP1.2, and SNMP2) belonging to two subfamilies in the antennal transcriptome of *H. halys*. SNMP3 was recently identified in Lepidoptera, and it is highly expressed in midguts (Zhang et al., 2020). We failed to identify SNMP3 in our transcriptomic analysis in antenna of *H. halys*.

The expression of olfactory genes in non-olfactory tissues is very common among insect species (Sheng et al., 2017; Kang et al., 2018). These olfactory genes are assumed to be linked to host plant location or the synthesis of insect pheromone. In the study, we also found that many OBPs and CSPs are highly expressed in non-olfactory tissues. *HhalOBP1*, *HhalOBP24*, *HhalOBP32*, and *HhalCSP10* are highly expressed in the abdomen, whereas *HhalOBP40–42* are highly expressed in legs, suggesting some special functions of these HhalOBPs (Zhang et al., 2016; Bin et al., 2017; Wang G.Y. et al., 2017).

## Conclusion

In summary, we sequenced and annotated the chemosensory gene profiles in the antennal transcriptome of male and female adults of the brown marmorated stink bug, *H. halys*. A total of 241 chemosensory genes including 138 ORs, 24 IRs, 15 GRs, 44 OBPs, 17 CSPs, and three SNMPs were identified in the antennal transcriptome. We also found tissue-specific expression of *HhalOBP1*, *HhalOBP24*, *HhalOBP32*, *HhalOBP40–42*, and *HhalCSP2*, as well as sex-specific expression of *HhalOBP10* and *HhalOBP40*. The huge number of olfactory genes, as well as tissue- and sex-specific expression of some CSPs in *H. halys* antennal transcriptome, suggests a range of diverse functions of insect antennae, which, to a greater degree, may facilitate the survival of insects in environments full of infochemicals from hosts, mates, and enemies. Data from the present study may also provide a basis for additional insight into olfactory mechanisms at the molecular level and for the development of environmentally friendly management strategies for *H. halys* in the future.

## Data Availability Statement

The clean reads of the two transcriptomes produced in this study are stored in the NCBI SRA database under the accession numbers SRR11748354 (female antennae) and SRR11747758 (male antennae).

## Author Contributions

DS, YH, ZQ, and HZ conducted the experiment. DS, YH, ZQ, YL, and SY wrote the manuscript. JZ, YL, and SY conceived the experiment. YL and DS analyzed the data. YH revised the manuscript and interpreted the data. All authors read and approved the final manuscript.

## Conflict of Interest

The authors declare that the research was conducted in the absence of any commercial or financial relationships that could be construed as a potential conflict of interest.

## Footnotes

1 http://web.expasy.org/translate/

2 http://www.cbs.dtu.dk/services/SignalP/

3 http://www.cbs.dtu.dk/services/TMHMM/

4 https://www.ebi.ac.uk/Tools/msa/mafft/

## Supplementary Material

FIGURE S1 Sequence alignment of HhalOBPs. Conserved cysteines of HhalOBPs were marked with “C.” Plus-C OBPs: green; Classic OBPs: blue.

FIGURE S2 Sequences alignment of HhalCSPs. Conserved cysteines of HhalCSPs were shown by C1–C4 which marked with a black triangle, respectively.

TABLE S1 Primers for RT-PCR expression analyses of *HhalOBP* and *HhalCSP* genes.

TABLE S2 Assembly summary of *Halyomorpha halys* transcriptome.

TABLE S3 Unigenes of candidate olfactory receptors in *Halyomorpha halys.*

TABLE S4 Unigenes of candidate ionotropic receptors in *Halyomorpha halys.*

TABLE S5 Unigenes of candidate gustatory receptors in *Halyomorpha halys.*

TABLE S6 Unigenes of candidate odorant-binding proteins in *Halyomorpha halys.*

TABLE S7 Unigenes of candidate chemosensory proteins in *Halyomorpha halys.*

TABLE S8 Unigenes of candidate sensory neuron membrane proteins in *Halyomorpha halys.*

FILE S1 The amino acid sequences of the chemosensory proteins identified in this study.
