# Supplementary figures and images for: Identification of Candidate Olfactory Genes in the Antennal Transcriptome of the Stink Bug Halyomorpha halys
Source: Front Physiol. 2020 Jul 24;11:876. doi: 10.3389/fphys.2020.00876 (PMC7394822; doi:10.3389/fphys.2020.00876)

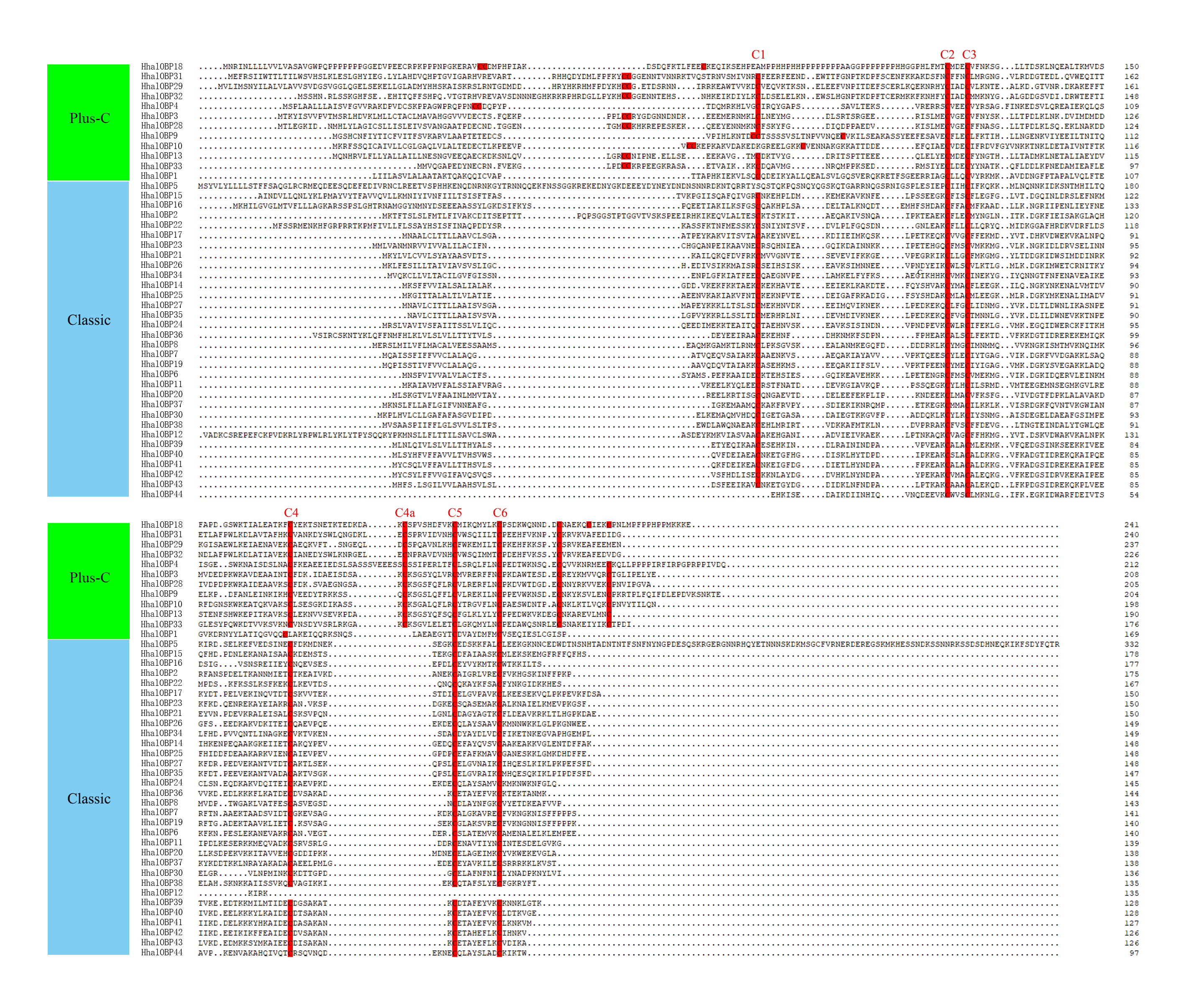

Supplement: FIGURE S1 — Sequence alignment of HhalOBPs. Conserved cysteines of HhalOBPs were marked with “C.” Plus-C OBPs: green; Classic OBPs: blue. [file Image_1.TIF]

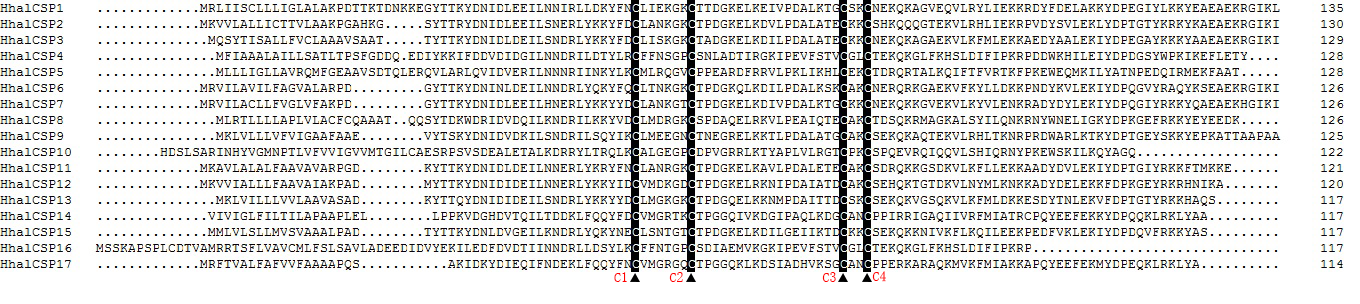

Supplement: FIGURE S2 — Sequences alignment of HhalCSPs. Conserved cysteines of HhalCSPs were shown by C1–C4 which marked with a black triangle, respectively. [file Image_2.TIF]
