# Supplementary material for: Identification of Candidate Olfactory Genes in the Antennal Transcriptome of the Stink Bug Halyomorpha halys
Source: Front Physiol. 2020 Jul 24;11:876. doi: 10.3389/fphys.2020.00876 (PMC7394822; doi:10.3389/fphys.2020.00876)
Supplement: TABLE S1 — Primers for RT-PCR expression analyses of HhalOBP and HhalCSP genes. [file Table_1.DOCX]

Table S1. Primers for RT-PCR expression analyses of HhalOBPs and HhalCSPs

| **Primer name** | **Forward Primer (5' to 3')** | **Reverse Primer (5' to 3')** |
| --- | --- | --- |
| HhalOBP1 | GGATGATAACGGTTTTCCTACAG | TCAATCTGCTCACTCACACACAT |
| HhalOBP2 | GTCTGAGCCAACAACTACACCAC | CATGCTTTACAAAGCATTCTCG |
| HhalOBP3 | TGCTTCTCTGCACTGCTTGTCTA | AGTCGCTTATCTCGGCATCTATT |
| HhalOBP4 | TATCTGTTTTCGGGGTAGTAAGG | GAAGAAGTTGTTTGCATTCCTCC |
| HhalOBP5 | TGAAGAGTCCCAAGATGAGTTTG | GCTCTTTCGATTATTGCTTGATT |
| HhalOBP6 | ATCGTTGTTGCACTTGTACTCG | TCTGTAGCTAAGGAGCACCGTT |
| HhalOBP7 | TATCTTCTTCGTAGTCTGCCTTGC | CTTACGGCCTTTCCTAGAGCAC |
| HhalOBP8 | GAGAGGAGCTTGATGATCTTGG | TACGAATGCCTCTTTGTCAGTTT |
| HhalOBP9 | TACCAATCCTGTTGTGAATCAGG | CCAAACTTCAGGAGGACAGTTCA |
| HhalOBP10 | ACCAGAAGAAGTTCCAGTTTGCT | TGTTTTGAGTTTATTACAGGCGG |
| HhalOBP11 | TATTGCATTCGTTAGAGCTGGC | AGCACTTATCAGCGACTTGTTCC |
| HhalOBP12 | TTCTCTCTGCTGTTTGCCTGTC | GAGAGCCTTCACTTTAGCCCAG |
| HhalOBP13 | TCACCGTGTTCTTTTCTTATTGT | GTAAGAGCCACTCTTGCATTTG |
| HhalOBP14 | AGAGGAAATTGAAAAGCTGAAGG | ATCAGTATTCTCCAAGCCCACC |
| HhalOBP15 | GTTTTACCTTTGCCAGTACGGTC | GTGAAACTGGAAGAACCTGAAGC |
| HhalOBP16 | TTTCTACTCTTAGCTGGGAAGGC | GGTCCAACATTTGGTCATTTTGT |
| HhalOBP17 | CACTCTGCCTTACCACTCTTCTG | TCAAACTTCACTTCAGGTTTTGG |
| HhalOBP18 | GCGACCAATTCAAAACACTCTTC | GAGGGGCATTTCAAATACATCTG |
| HhalOBP19 | CTCCACCATCGTCTTCGTAGTCT | TTGTTTCCATTCTTGACGAAGC |
| HhalOBP20 | TGGAGCTGAAGTTACTGATGATG | CCTCCTTCTCCCATTTTACATAG |
| HhalOBP21 | CTGTGTTGTTCTCTCCTACGCC | CGTCAACTTACGCTTTACTGCC |
| HhalOBP22 | TGCTTACCATTCCATCAGTTTTA | TTCCCTTATTGTAAAAGCATGC |
| HhalOBP23 | AGTCGCCAACATGAACAGAGTAG | GAGCACATTTTGCCATCTCTGA |
| HhalOBP24 | GGTCCTTGGTCGCTGTCATAGT | GCAAACCATCGCACTATATGCT |
| HhalOBP25 | TGAAGGGTATTACGACTGCCTT | GCTCCACATACAGCCATCTTAA |
| HhalOBP26 | CCATTGTCATCGCTGTTAGTGTC | CCAAGCTTCTTCCAGTTGTTCA |
| HhalOBP27 | TGAACGCTGTACTCTGTATCACC | TACATTTTATCGCATTTACTCCC |
| HhalOBP28 | TAGTGTCTGTGGCTAATGGTGCT | CATTCATCACCATCAGTCCACAC |
| HhalOBP29 | GCATTAGTCTTGGCTGTGGTGTC | CCATCTTTCAACGCTTCAGTGTT |
| HhalOBP30 | CACTACACGTCTTGTGCCTCCT | CAGTTCACAACCATCTGGACCA |
| HhalOBP31 | AGGGAAGTAGCAAGAACTAGGCA | GCCAAAGTTTCCGTTGTTATCTG |
| HhalOBP32 | TTTCTTCCAAAGGTCACTTCTCC | GCAATACATTTCTCAACAGCGAT |
| HhalOBP33 | CGCTCCAGAAGACTACAATGAAT | AGGTTTCTAGCTCCAATACCCC |
| HhalOBP34 | TATTGGTGTTGACTGCCTGTATC | GACCCCCTCCTTATTTGTTTCT |
| HhalOBP35 | AGTGTTTCGTTGGCTGTACCAT | CTGATGCATACATTTTATCGCC |
| HhalOBP36 | TGAAGAGATTAGGGCTGCATGT | GGCAGTCTTTTCTGTCTTGCAT |
| HhalOBP37 | GCTTTTGGAATTGGTAAAGAGAT | CCTAACATTGGTAATTCCTCTGC |
| HhalOBP38 | TCTCCCATCATCTTCTTCCTCG | ACTCATAGAGGGAAAATGCGGT |
| HhalOBP39 | CTACACATTATGCCCTGTCCGA | TTCTTGGTATCTTCTTCCTTCACTG |
| HhalOBP40 | TCTTACCATTTTGTTTTCTTTGCTG | TTCACATTTATTGGCCTTAGCAG |
| HhalOBP41 | TAAAGAAATTGGATTCGATGGAG | GCACTTCACAAACTCGTAAGCTG |
| HhalOBP42 | GCAGTGCAATCTGTTCAATCAGT | AGCTGATACATCGCATTCATCA |
| HhalOBP43 | GGAGACTGGATATGATGGCGAT | CCACGCATTTCAGGAACTCATA |
| HhalOBP44 | GAAGGTAAAATCGACTGGGCTC | TGCAGTCTGCCAAAGAGTAAGC |
|  |  |  |
| HhalCSP1 | TGACACCACCAAGACCGACAAC | TTCTCAGCCTCAGCTTCGTATTTC |
| HhalCSP2 | CGTCCTAGCTTTGCTCATCTGTA | TTCTCAGCCTCAGCTTTGTATTT |
| HhalCSP3 | ATTTCTGCCCTTCTTTTCGTCT | AGCGGCGTATTTCTTCTTGTAT |
| HhalCSP4 | ATCTTGCTATCGGCAACTTTGA | GGAGCCATCAGGATCGTAGATT |
| HhalCSP5 | GTTGCTCCTCATTGGGCTACTT | TTTGATCTTCAGGGTTTGTCGC |
| HhalCSP6 | CAGTCATCCTCTTCGCAGGAGT | CCTCTTTTCGGCTTCACTCTTG |
| HhalCSP7 | GTCTATTGTTCGTCGGATTGGT | TCGTAGTCAGCCCTCTTGTTCT |
| HhalCSP8 | ACTCCTCCTTGCTCCCCTCGTGT | CATTCCAGTAGTTTCTTTTGTTTTG |
| HhalCSP9 | CTGGTACTCCTCTTGGTCTTCG | CTGTAGTCGCCTTAGGCTCGTA |
| HhalCSP10 | GTTTTCGTTGTTATCGGCGTAG | TGGATCTGTCGGACTTCTTGTG |
| HhalCSP11 | AGTCCTCGCCCTAGCTCTGTTC | GTCGTAGTCGGCTGCCTTCTTT |
| HhalCSP12 | GCACTCTTATTATTCGCTGCAGT | GATGTTGTGCCTTTTCCTGTATT |
| HhalCSP13 | GCTTGTTATCTTGTTGTTGGTTG | CCAAATTGGTATAGTCGGACTCC |
| HhalCSP14 | ACGGTATTCCTGCACAGTTAAAG | ATTTCTGCTGGGGGTCATACTTC |
| HhalCSP15 | CTGGTCCTTTCACTGTTGATGGT | TGAACACTTGGTCTGGATCGTAA |
| HhalCSP16 | AGGCGGACTTCTTTCTTGGTAG | AGGGATGAAGATGTCCAAACTG |
| HhalCSP17 | TCCACAGTCAGCCAAGATTGAC | GTTCGGGGTCATACATTTTTTC |
|  |  |  |
| HhalActin | TGAACACGGTATCATCACCAACT | CATAGCACAGCTTTTCCTTGATG |
