# Supplementary material for: Identification of Candidate Olfactory Genes in the Antennal Transcriptome of the Stink Bug Halyomorpha halys
Source: Front Physiol. 2020 Jul 24;11:876. doi: 10.3389/fphys.2020.00876 (PMC7394822; doi:10.3389/fphys.2020.00876)
Supplement: TABLE S2 — Assembly summary of Halyomorpha halys transcriptome. [file Table_2.DOCX]

Table S2. Assembly summary of *Halyomorpha halys* transcriptome

|  | Sample | Total Number | Total Length (nt) | Mean Length (nt) | N50 (nt) |
| --- | --- | --- | --- | --- | --- |
| Contig | FA | 79,725 | 70,307,662 | 881 | 1,835 |
|  | MA | 97,219 | 77,356,409 | 795 | 1,634 |
| Unigene | FA | 48,875 | 53,345,551 | 1,091 | 2,205 |
|  | MA | 58,935 | 57,946,681 | 983 | 2,038 |
| Merge | All | 65,914 | 71,522,269 | 1,085 | 2,342 |

Note: FA: Female antennae; MA: Male antennae.
