# Supplementary material for: Identification of Candidate Olfactory Genes in the Antennal Transcriptome of the Stink Bug Halyomorpha halys
Source: Front Physiol. 2020 Jul 24;11:876. doi: 10.3389/fphys.2020.00876 (PMC7394822; doi:10.3389/fphys.2020.00876)
Supplement: TABLE S3 — Unigenes of candidate olfactory receptors in Halyomorpha halys. [file Table_3.DOCX]

Table S3. Unigenes of candidate olfactory receptors in *Halyomorpha halys*

| **Name** | **Unigene reference** | **length**  **(nt)** | **ORF**  **(aa)** | **Status** | **TMD**  **(No)** | **E_value** | **Best blastx hit** |
| --- | --- | --- | --- | --- | --- | --- | --- |
| HhalOrco | Unigene19235 | 4200 | 474 | Full | 7 | 0 | XP_014279419.1 PREDICTED: odorant receptor coreceptor isoform X1 [Halyomorpha halys] |
| HhalOR1 | CL3650.Contig2 | 1447 | 447 | 5' lost | 7 | 0 | XP_014287248.1 PREDICTED: uncharacterized protein LOC106687729 [Halyomorpha halys] |
| HhalOR2 | CL5483.Contig1 | 1453 | 442 | Full | 6 | 5.00E-171 | XP_014273407.1 PREDICTED: uncharacterized protein LOC106679017 isoform X1 [Halyomorpha halys] |
| HhalOR3 | Unigene1827 | 1456 | 438 | Full | 6 | 0 | XP_014286277.1 PREDICTED: uncharacterized protein LOC106687100 [Halyomorpha halys] |
| HhalOR4 | CL954.Contig3 | 1521 | 437 | Full | 6 | 0 | XP_014273269.1 PREDICTED: uncharacterized protein LOC106678921 isoform X1 [Halyomorpha halys] |
| HhalOR5 | CL954.Contig4 | 1527 | 437 | Full | 6 | 0 | XP_014273269.1 PREDICTED: uncharacterized protein LOC106678921 isoform X1 [Halyomorpha halys] |
| HhalOR6 | CL2031.Contig2 | 1409 | 435 | Full | 6 | 0 | XP_014276987.1 PREDICTED: uncharacterized protein LOC106681266 [Halyomorpha halys] |
| HhalOR7 | Unigene19132 | 1438 | 434 | Full | 8 | 2.00E-90 | XP_014272634.1 PREDICTED: odorant receptor 67c-like [Halyomorpha halys] |
| HhalOR8 | Unigene9739 | 1448 | 434 | Full | 6 | 0 | XP_014272658.1 PREDICTED: uncharacterized protein LOC106678586 [Halyomorpha halys] |
| HhalOR9 | CL6143.Contig1 | 1527 | 433 | Full | 7 | 0 | XP_014287615.1 PREDICTED: uncharacterized protein LOC106687938 [Halyomorpha halys] |
| HhalOR10 | CL1808.Contig2 | 1955 | 430 | Full | 6 | 0 | XP_014290637.1 PREDICTED: uncharacterized protein LOC106689927 isoform X1 [Halyomorpha halys] |
| HhalOR11 | CL3701.Contig4 | 1330 | 430 | Full | 6 | 0 | XP_014287936.1 PREDICTED: uncharacterized protein LOC106688133 isoform X1 [Halyomorpha halys] |
| HhalOR12 | CL929.Contig2 | 1405 | 430 | 5' lost | 5 | 0 | XP_014291338.1 PREDICTED: uncharacterized protein LOC106690411 [Halyomorpha halys] |
| HhalOR13 | CL4980.Contig2 | 1474 | 428 | 5' lost | 6 | 0 | XP_014289234.1 PREDICTED: odorant receptor 24a-like [Halyomorpha halys] |
| HhalOR14 | CL5060.Contig1 | 1386 | 428 | Full | 6 | 0 | XP_014294765.1 PREDICTED: odorant receptor 4-like [Halyomorpha halys] |
| HhalOR15 | CL126.Contig1 | 1408 | 427 | Full | 6 | 0 | XP_014273407.1 PREDICTED: uncharacterized protein LOC106679017 isoform X1 [Halyomorpha halys] |
| HhalOR16 | Unigene7368 | 1816 | 427 | Full | 6 | 0 | XP_014274444.1 PREDICTED: odorant receptor 47a-like [Halyomorpha halys] |
| HhalOR17 | Unigene11703 | 1493 | 426 | Full | 6 | 0 | XP_014276985.1 PREDICTED: odorant receptor 22c-like [Halyomorpha halys] |
| HhalOR18 | Unigene15249 | 3878 | 426 | Full | 6 | 0 | XP_014271872.1 PREDICTED: uncharacterized protein LOC106678083 [Halyomorpha halys] |
| HhalOR19 | Unigene19530 | 1493 | 423 | Full | 6 | 5.00E-69 | XP_014287487.1 PREDICTED: putative odorant receptor 71a [Halyomorpha halys] |
| HhalOR20 | Unigene14029 | 1329 | 421 | 5' lost | 5 | 0 | XP_014280612.1 PREDICTED: odorant receptor 24a-like [Halyomorpha halys] |
| HhalOR21 | CL1884.Contig2 | 1516 | 420 | Full | 7 | 0 | XP_014290805.1 PREDICTED: odorant receptor 22c-like [Halyomorpha halys] |
| HhalOR22 | CL2964.Contig1 | 1635 | 420 | Full | 7 | 0 | XP_014291335.1 PREDICTED: uncharacterized protein LOC106690407, partial [Halyomorpha halys] |
| HhalOR23 | Unigene5764 | 1376 | 420 | Full | 6 | 0 | XP_014284895.1 PREDICTED: uncharacterized protein LOC106686230 [Halyomorpha halys] |
| HhalOR24 | CL3239.Contig3 | 1378 | 419 | 5' lost | 5 | 0 | XP_014280634.1 PREDICTED: uncharacterized protein LOC106683586, partial [Halyomorpha halys] |
| HhalOR25 | CL3239.Contig5 | 1391 | 419 | 5' lost | 5 | 0 | XP_014294791.1 PREDICTED: odorant receptor 30a-like [Halyomorpha halys] |
| HhalOR26 | CL4540.Contig6 | 1461 | 419 | Full | 7 | 0 | XP_014290808.1 PREDICTED: uncharacterized protein LOC106690056 [Halyomorpha halys] |
| HhalOR27 | CL5085.Contig2 | 1427 | 419 | Full | 6 | 0 | XP_014270732.1 PREDICTED: uncharacterized protein LOC106677363 isoform X1 [Halyomorpha halys] |
| HhalOR28 | Unigene17173 | 1578 | 419 | 5' lost | 6 | 6.00E-179 | XP_014287758.1 PREDICTED: uncharacterized protein LOC106688013 [Halyomorpha halys] |
| HhalOR29 | Unigene19537 | 1413 | 419 | Full | 5 | 0 | XP_014280696.1 PREDICTED: odorant receptor 82a [Halyomorpha halys] |
| HhalOR30 | CL4540.Contig3 | 1449 | 418 | Full | 5 | 0 | XP_014290804.1 PREDICTED: odorant receptor 4-like [Halyomorpha halys] |
| HhalOR31 | Unigene407 | 1466 | 418 | 5' lost | 5 | 0 | XP_014276741.1 PREDICTED: odorant receptor 94a-like [Halyomorpha halys] |
| HhalOR32 | CL2995.Contig2 | 1430 | 417 | Full | 6 | 0 | XP_014278702.1 PREDICTED: odorant receptor 67c-like [Halyomorpha halys] |
| HhalOR33 | CL4146.Contig2 | 1833 | 417 | Full | 6 | 0 | XP_014293234.1 PREDICTED: odorant receptor 59b-like [Halyomorpha halys] |
| HhalOR34 | Unigene13246 | 1600 | 417 | Full | 6 | 3.00E-135 | XP_014287492.1 PREDICTED: odorant receptor 4-like [Halyomorpha halys] |
| HhalOR35 | Unigene3873 | 1459 | 417 | Full | 7 | 0 | XP_014289256.1 PREDICTED: uncharacterized protein LOC106689027, partial [Halyomorpha halys] |
| HhalOR36 | CL3239.Contig9 | 1372 | 416 | 5' lost | 4 | 0 | XP_014280756.1 PREDICTED: odorant receptor 7a-like [Halyomorpha halys] |
| HhalOR37 | Unigene3799 | 1370 | 416 | Full | 5 | 1.00E-180 | XP_014280696.1 PREDICTED: odorant receptor 82a [Halyomorpha halys] |
| HhalOR38 | CL4540.Contig5 | 1504 | 415 | Full | 5 | 0 | XP_014290804.1 PREDICTED: odorant receptor 4-like [Halyomorpha halys] |
| HhalOR39 | Unigene10277 | 1530 | 415 | Full | 5 | 0 | XP_014294789.1 PREDICTED: uncharacterized protein LOC106693004 [Halyomorpha halys] |
| HhalOR40 | Unigene11932 | 1348 | 415 | Full | 5 | 0 | XP_014280623.1 PREDICTED: odorant receptor 24a-like [Halyomorpha halys] |
| HhalOR41 | Unigene17161 | 1390 | 415 | 5' lost | 6 | 0 | XP_014289020.1 PREDICTED: odorant receptor 43a-like [Halyomorpha halys] |
| HhalOR42 | Unigene314 | 1440 | 415 | Full | 7 | 0 | XP_014290195.1 PREDICTED: uncharacterized protein LOC106689626, partial [Halyomorpha halys] |
| HhalOR43 | Unigene3403 | 1394 | 415 | Full | 6 | 0 | XP_014294793.1 PREDICTED: odorant receptor 24a-like [Halyomorpha halys] |
| HhalOR44 | Unigene3477 | 2862 | 415 | Full | 6 | 0 | XP_014288483.1 PREDICTED: uncharacterized protein LOC106688504 [Halyomorpha halys] |
| HhalOR45 | CL5493.Contig1 | 1335 | 414 | Full | 5 | 0 | XP_014280768.1 PREDICTED: odorant receptor 24a-like isoform X1 [Halyomorpha halys] |
| HhalOR46 | Unigene11693 | 1494 | 414 | Full | 6 | 0 | XP_014288391.1 PREDICTED: odorant receptor 24a [Halyomorpha halys] |
| HhalOR47 | Unigene2463 | 1287 | 414 | 5' lost | 6 | 0 | XP_014280623.1 PREDICTED: odorant receptor 24a-like [Halyomorpha halys] |
| HhalOR48 | Unigene6269 | 2449 | 414 | Full | 5 | 0 | XP_014270184.1 PREDICTED: odorant receptor 24a-like [Halyomorpha halys] |
| HhalOR49 | Unigene7364 | 1670 | 414 | Full | 6 | 0 | XP_014287492.1 PREDICTED: odorant receptor 4-like [Halyomorpha halys] |
| HhalOR50 | CL5158.Contig2 | 1390 | 413 | Full | 6 | 0 | XP_014280245.1 PREDICTED: uncharacterized protein LOC106683353 isoform X1 [Halyomorpha halys] |
| HhalOR51 | CL5396.Contig1 | 1351 | 412 | Full | 7 | 0 | XP_014294790.1 PREDICTED: uncharacterized protein LOC106693006 [Halyomorpha halys] |
| HhalOR52 | Unigene17163 | 1637 | 412 | Full | 6 | 0 | XP_014275211.1 PREDICTED: odorant receptor 24a-like [Halyomorpha halys] |
| HhalOR53 | Unigene6422 | 1407 | 412 | 5' lost | 5 | 0 | XP_014282484.1 PREDICTED: putative odorant receptor 85d isoform X1 [Halyomorpha halys] |
| HhalOR54 | CL3239.Contig4 | 1248 | 411 | 3' lost | 7 | 0 | XP_014294797.1 PREDICTED: uncharacterized protein LOC106693012 [Halyomorpha halys] |
| HhalOR55 | Unigene30267 | 1363 | 411 | Full | 6 | 0 | XP_014280723.1 PREDICTED: odorant receptor 82a-like [Halyomorpha halys] |
| HhalOR56 | Unigene3791 | 1485 | 411 | Full | 6 | 0 | XP_014292012.1 PREDICTED: odorant receptor 22c-like [Halyomorpha halys] |
| HhalOR57 | CL65.Contig2 | 1388 | 410 | Full | 6 | 0 | XP_014280732.1 PREDICTED: uncharacterized protein LOC106683640 [Halyomorpha halys] |
| HhalOR58 | Unigene10105 | 1786 | 409 | Full | 6 | 6.00E-123 | XP_014292012.1 PREDICTED: odorant receptor 22c-like [Halyomorpha halys] |
| HhalOR59 | CL4084.Contig1 | 1382 | 408 | 5' lost | 5 | 0 | XP_014272447.1 PREDICTED: odorant receptor 24a-like [Halyomorpha halys] |
| HhalOR60 | CL466.Contig3 | 1419 | 408 | Full | 6 | 0 | XP_014270188.1 PREDICTED: odorant receptor 4-like isoform X1 [Halyomorpha halys] |
| HhalOR61 | Unigene11245 | 1455 | 408 | 5' lost | 6 | 2.00E-145 | XP_014290613.1 PREDICTED: odorant receptor 4-like [Halyomorpha halys] |
| HhalOR62 | Unigene123 | 1531 | 408 | Full | 6 | 0 | XP_014270188.1 PREDICTED: odorant receptor 4-like isoform X1 [Halyomorpha halys] |
| HhalOR63 | Unigene16298 | 2471 | 408 | Full | 6 | 0 | XP_014291074.1 PREDICTED: odorant receptor 46a, isoform B-like [Halyomorpha halys] |
| HhalOR64 | CL2501.Contig1 | 1350 | 407 | Full | 7 | 0 | XP_014289383.1 PREDICTED: uncharacterized protein LOC106689110 [Halyomorpha halys] |
| HhalOR65 | CL6007.Contig1 | 1864 | 407 | Full | 5 | 0 | XP_014278793.1 PREDICTED: uncharacterized protein LOC106682449 isoform X1 [Halyomorpha halys] |
| HhalOR66 | Unigene18183 | 1413 | 407 | 5' lost | 5 | 0 | XP_014276746.1 PREDICTED: odorant receptor 4-like [Halyomorpha halys] |
| HhalOR67 | Unigene17578 | 1449 | 406 | Full | 6 | 0 | XP_014281821.1 PREDICTED: odorant receptor 33a-like [Halyomorpha halys] |
| HhalOR68 | CL1241.Contig1 | 1559 | 404 | Full | 6 | 0 | XP_014270725.1 PREDICTED: uncharacterized protein LOC106677357 isoform X1 [Halyomorpha halys] |
| HhalOR69 | CL1700.Contig1 | 1356 | 404 | Full | 7 | 0 | XP_014272140.1 PREDICTED: uncharacterized protein LOC106678240 [Halyomorpha halys] |
| HhalOR70 | Unigene5787 | 1275 | 404 | Full | 7 | 0 | XP_014277766.1 PREDICTED: uncharacterized protein LOC106681777 [Halyomorpha halys] |
| HhalOR71 | Unigene13621 | 1353 | 403 | 5' lost | 5 | 0 | XP_014289200.1 PREDICTED: odorant receptor 82a-like [Halyomorpha halys] |
| HhalOR72 | Unigene15628 | 1257 | 403 | 5' lost | 5 | 0 | XP_014277890.1 PREDICTED: uncharacterized protein LOC106681860 [Halyomorpha halys] |
| HhalOR73 | CL3811.Contig1 | 1378 | 402 | Full | 6 | 0 | XP_014293678.1 PREDICTED: uncharacterized protein LOC106692293, partial [Halyomorpha halys] |
| HhalOR74 | Unigene16125 | 1396 | 402 | Full | 5 | 0 | XP_014289011.1 PREDICTED: uncharacterized protein LOC106688852 [Halyomorpha halys] |
| HhalOR75 | Unigene9690 | 1377 | 402 | Full | 6 | 0 | XP_014289021.1 PREDICTED: uncharacterized protein LOC106688860 [Halyomorpha halys] |
| HhalOR76 | CL5097.Contig1 | 1279 | 401 | 5' lost | 6 | 0 | XP_014277878.1 PREDICTED: uncharacterized protein LOC106681850 [Halyomorpha halys] |
| HhalOR77 | Unigene14237 | 1328 | 401 | Full | 6 | 0 | XP_014289023.1 PREDICTED: uncharacterized protein LOC106688861 [Halyomorpha halys] |
| HhalOR78 | Unigene15607 | 1329 | 401 | Full | 6 | 0 | XP_014289019.1 PREDICTED: uncharacterized protein LOC106688858 [Halyomorpha halys] |
| HhalOR79 | Unigene17160 | 1380 | 400 | Full | 6 | 0 | XP_014281345.1 PREDICTED: uncharacterized protein LOC106684029 isoform X1 [Halyomorpha halys] |
| HhalOR80 | Unigene18111 | 1283 | 400 | Full | 5 | 0 | XP_014275988.1 PREDICTED: odorant receptor 22c-like [Halyomorpha halys] |
| HhalOR81 | Unigene19555 | 1266 | 400 | Full | 6 | 0 | XP_014276740.1 PREDICTED: uncharacterized protein LOC106681099 [Halyomorpha halys] |
| HhalOR82 | CL4438.Contig1 | 1344 | 399 | 5' lost | 7 | 5.00E-169 | XP_014275003.1 PREDICTED: odorant receptor 49b-like [Halyomorpha halys] |
| HhalOR83 | Unigene13640 | 1325 | 399 | Full | 6 | 0 | XP_014292083.1 PREDICTED: odorant receptor 85b-like [Halyomorpha halys] |
| HhalOR84 | Unigene15599 | 1349 | 399 | Full | 6 | 0 | XP_014288559.1 PREDICTED: uncharacterized protein LOC106688565 [Halyomorpha halys] |
| HhalOR85 | Unigene17543 | 1310 | 399 | Full | 5 | 8.00E-149 | XP_014275988.1 PREDICTED: odorant receptor 22c-like [Halyomorpha halys] |
| HhalOR86 | Unigene19521 | 1319 | 399 | Full | 7 | 0 | XP_014272535.1 PREDICTED: odorant receptor 85b-like [Halyomorpha halys] |
| HhalOR87 | CL3811.Contig3 | 1363 | 398 | 5' lost | 6 | 0 | XP_014289013.1 PREDICTED: uncharacterized protein LOC106688854 [Halyomorpha halys] |
| HhalOR88 | CL5519.Contig1 | 2860 | 398 | 5' lost | 6 | 8.00E-158 | XP_014287265.1 PREDICTED: uncharacterized protein LOC106687743 [Halyomorpha halys] |
| HhalOR89 | Unigene13238 | 1403 | 398 | Full | 7 | 0 | XP_014274899.1 PREDICTED: uncharacterized protein LOC106679982 [Halyomorpha halys] |
| HhalOR90 | Unigene17539 | 1332 | 398 | Full | 3 | 0 | XP_014270724.1 PREDICTED: uncharacterized protein LOC106677356 [Halyomorpha halys] |
| HhalOR91 | CL3632.Contig1 | 1779 | 397 | Full | 6 | 0 | XP_014281005.1 PREDICTED: odorant receptor 4-like [Halyomorpha halys] |
| HhalOR92 | Unigene15632 | 1460 | 397 | 5' lost | 6 | 4.00E-166 | XP_014287043.1 PREDICTED: uncharacterized protein LOC106687583 [Halyomorpha halys] |
| HhalOR93 | CL2734.Contig2 | 1315 | 395 | 5' lost | 6 | 0 | XP_014292085.1 PREDICTED: uncharacterized protein LOC106690974 [Halyomorpha halys] |
| HhalOR94 | CL2777.Contig1 | 1320 | 393 | Full | 7 | 0 | XP_014281510.1 PREDICTED: odorant receptor 49b-like [Halyomorpha halys] |
| HhalOR95 | CL4084.Contig2 | 1181 | 393 | 5', 3' lost | 5 | 0 | XP_014272447.1 PREDICTED: odorant receptor 24a-like [Halyomorpha halys] |
| HhalOR96 | Unigene13234 | 1437 | 393 | Full | 6 | 0 | XP_014275003.1 PREDICTED: odorant receptor 49b-like [Halyomorpha halys] |
| HhalOR97 | Unigene2475 | 2709 | 392 | Full | 6 | 0 | XP_014281239.1 PREDICTED: uncharacterized protein LOC106683969 [Halyomorpha halys] |
| HhalOR98 | CL2947.Contig1 | 1470 | 391 | Full | 7 | 0 | XP_014289015.1 PREDICTED: uncharacterized protein LOC106688856 isoform X1 [Halyomorpha halys] |
| HhalOR99 | CL4689.Contig1 | 1250 | 390 | 5' lost | 3 | 0 | XP_014293574.1 PREDICTED: uncharacterized protein LOC106692148 [Halyomorpha halys] |
| HhalOR100 | Unigene15603 | 1429 | 390 | Full | 6 | 4.00E-136 | XP_014287042.1 PREDICTED: odorant receptor 43b-like [Halyomorpha halys] |
| HhalOR101 | Unigene17174 | 1524 | 390 | 5' lost | 6 | 2.00E-46 | XP_014293519.1 PREDICTED: uncharacterized protein LOC106692125, partial [Halyomorpha halys] |
| HhalOR102 | Unigene19524 | 1322 | 390 | Full | 4 | 0 | XP_014287044.1 PREDICTED: uncharacterized protein LOC106687584 [Halyomorpha halys] |
| HhalOR103 | CL3222.Contig1 | 2010 | 389 | Full | 7 | 0 | XP_014287042.1 PREDICTED: odorant receptor 43b-like [Halyomorpha halys] |
| HhalOR104 | Unigene19127 | 1459 | 389 | Full | 6 | 0 | XP_014274900.1 PREDICTED: odorant receptor 30a-like [Halyomorpha halys] |
| HhalOR105 | Unigene7348 | 1365 | 389 | Full | 6 | 0 | XP_014287040.1 PREDICTED: odorant receptor 4-like [Halyomorpha halys] |
| HhalOR106 | Unigene7753 | 1298 | 389 | Full | 6 | 0 | XP_014293859.1 PREDICTED: odorant receptor 85b-like isoform X1 [Halyomorpha halys] |
| HhalOR107 | Unigene19519 | 1360 | 388 | Full | 6 | 4.00E-176 | XP_014278714.1 PREDICTED: uncharacterized protein LOC106682407, partial [Halyomorpha halys] |
| HhalOR108 | Unigene15591 | 1315 | 385 | Full | 6 | 2.00E-62 | XP_014293519.1 PREDICTED: uncharacterized protein LOC106692125, partial [Halyomorpha halys] |
| HhalOR109 | Unigene5766 | 2286 | 384 | Full | 6 | 0 | XP_014294439.1 PREDICTED: odorant receptor Or1-like isoform X1 [Halyomorpha halys] |
| HhalOR110 | Unigene3793 | 1321 | 383 | 5' lost | 5 | 4.00E-172 | XP_014291481.1 PREDICTED: putative odorant receptor 71a, partial [Halyomorpha halys] |
| HhalOR111 | CL3769.Contig2 | 1351 | 382 | Full | 7 | 0 | XP_014294442.1 PREDICTED: odorant receptor 9a-like [Halyomorpha halys] |
| HhalOR112 | CL2734.Contig3 | 1494 | 381 | Full | 6 | 0 | XP_014292085.1 PREDICTED: uncharacterized protein LOC106690974 [Halyomorpha halys] |
| HhalOR113 | Unigene3862 | 1362 | 381 | Full | 5 | 0 | XP_014293070.1 PREDICTED: uncharacterized protein LOC106691728, partial [Halyomorpha halys] |
| HhalOR114 | Unigene19531 | 1418 | 379 | Full | 4 | 2.00E-180 | XP_014278712.1 PREDICTED: odorant receptor 33a-like [Halyomorpha halys] |
| HhalOR115 | Unigene3821 | 1301 | 378 | Full | 5 | 0 | XP_014282544.1 PREDICTED: odorant receptor 4-like [Halyomorpha halys] |
| HhalOR116 | Unigene17611 | 1433 | 376 | 5' lost | 3 | 0 | XP_014278976.1 PREDICTED: uncharacterized protein LOC106682571 [Halyomorpha halys] |
| HhalOR117 | CL2528.Contig1 | 2224 | 374 | Full | 6 | 0 | XP_014287367.1 PREDICTED: odorant receptor 85b-like [Halyomorpha halys] |
| HhalOR118 | CL6198.Contig2 | 1346 | 373 | Full | 6 | 0 | XP_014286385.1 PREDICTED: putative odorant receptor 92a [Halyomorpha halys] |
| HhalOR119 | CL5771.Contig1 | 1380 | 372 | 5' lost | 6 | 0 | XP_014280744.1 PREDICTED: odorant receptor 4-like [Halyomorpha halys] |
| HhalOR120 | Unigene19594 | 1410 | 370 | 5' lost | 6 | 0 | XP_014292080.1 PREDICTED: uncharacterized protein LOC106690969 [Halyomorpha halys] |
| HhalOR121 | Unigene13607 | 1280 | 362 | 5' lost | 6 | 0 | XP_014288704.1 PREDICTED: odorant receptor 67c-like isoform X1 [Halyomorpha halys] |
| HhalOR122 | CL3650.Contig1 | 1356 | 355 | 5' lost | 7 | 0 | XP_014287248.1 PREDICTED: uncharacterized protein LOC106687729 [Halyomorpha halys] |
| HhalOR123 | Unigene6384 | 1121 | 353 | 5' lost | 6 | 2.00E-144 | XP_014281731.1 PREDICTED: uncharacterized protein LOC106684269 [Halyomorpha halys] |
| HhalOR124 | Unigene13231 | 1422 | 350 | Full | 6 | 0 | XP_014283226.1 PREDICTED: uncharacterized protein LOC106685185 [Halyomorpha halys] |
| HhalOR125 | Unigene7792 | 1423 | 338 | 5' lost | 3 | 0 | XP_014271039.1 PREDICTED: odorant receptor 83a-like [Halyomorpha halys] |
| HhalOR126 | CL4540.Contig2 | 1162 | 336 | 5' lost | 5 | 0 | XP_014290805.1 PREDICTED: odorant receptor 22c-like [Halyomorpha halys] |
| HhalOR127 | Unigene18074 | 1380 | 335 | 5' lost | 5 | 0 | XP_014280612.1 PREDICTED: odorant receptor 24a-like [Halyomorpha halys] |
| HhalOR128 | CL2777.Contig2 | 1926 | 333 | 5' lost | 6 | 2.00E-109 | XP_014281345.1 PREDICTED: uncharacterized protein LOC106684029 isoform X1 [Halyomorpha halys] |
| HhalOR129 | CL2501.Contig2 | 1510 | 322 | 5' lost | 6 | 1.00E-120 | XP_014289383.1 PREDICTED: uncharacterized protein LOC106689110 [Halyomorpha halys] |
| HhalOR130 | Unigene3798 | 1479 | 306 | Full | 4 | 4.00E-134 | XP_014276483.1 PREDICTED: uncharacterized protein LOC106680951 [Halyomorpha halys] |
| HhalOR131 | Unigene7755 | 1319 | 306 | Full | 4 | 0 | XP_014273330.1 PREDICTED: odorant receptor 85b-like [Halyomorpha halys] |
| HhalOR132 | CL65.Contig1 | 888 | 296 | 5', 3' lost | 4 | 0 | XP_014280732.1 PREDICTED: uncharacterized protein LOC106683640 [Halyomorpha halys] |
| HhalOR133 | Unigene17937 | 878 | 292 | 5', 3' lost | 4 | 5.00E-42 | XP_014287040.1 PREDICTED: odorant receptor 4-like [Halyomorpha halys] |
| HhalOR134 | CL4540.Contig4 | 801 | 266 | 5', 3' lost | 4 | 1.00E-147 | XP_014290808.1 PREDICTED: uncharacterized protein LOC106690056 [Halyomorpha halys] |
| HhalOR135 | Unigene13439 | 891 | 259 | 5' lost | 3 | 1.00E-174 | XP_014278702.1 PREDICTED: odorant receptor 67c-like [Halyomorpha halys] |
| HhalOR136 | Unigene9947 | 847 | 179 | 5' lost | 1 | 3.00E-88 | XP_014280612.1 PREDICTED: odorant receptor 24a-like [Halyomorpha halys] |
| HhalOR137 | CL3632.Contig2 | 979 | 168 | 5' lost | 1 | 3.00E-90 | XP_014281005.1 PREDICTED: odorant receptor 4-like [Halyomorpha halys] |
