# Supplementary material for: Identification of Candidate Olfactory Genes in the Antennal Transcriptome of the Stink Bug Halyomorpha halys
Source: Front Physiol. 2020 Jul 24;11:876. doi: 10.3389/fphys.2020.00876 (PMC7394822; doi:10.3389/fphys.2020.00876)
Supplement: TABLE S4 — Unigenes of candidate ionotropic receptors in Halyomorpha halys. [file Table_4.DOCX]

Table S4. Unigenes of candidate ionotropic receptors in *Halyomorpha halys*

| **Name** | **Unigene reference** | **length**  **(nt)** | **ORF**  **(aa)** | **Status** | **TMD**  **(No)** | **E_value** | **Best blastx hit** |
| --- | --- | --- | --- | --- | --- | --- | --- |
| HhalIR25a | Unigene19303 | 2959 | 931 | Full | 3 | 0 | XP_014277865.1 PREDICTED: glutamate receptor 4 [Halyomorpha halys] |
| HhalIR8a | Unigene9428 | 2647 | 864 | 5' lost | 3 | 0 | XP_014274775.1 PREDICTED: glutamate receptor ionotropic, kainate 3 [Halyomorpha halys] |
| HhalIR41c.1 | CL1110.Contig1 | 2126 | 640 | Full | 4 | 0 | XP_014289206.1 PREDICTED: glutamate receptor ionotropic, NMDA 2C-like [Halyomorpha halys] |
| HhalIR92a | Unigene4061 | 1957 | 632 | 5' lost | 3 | 0 | XP_014283840.1 PREDICTED: glutamate receptor 2-like [Halyomorpha halys] |
| HhalIR75b.2 | Unigene5412 | 1910 | 620 | 5' lost | 4 | 0 | XP_014277787.1 PREDICTED: uncharacterized protein LOC106681790 [Halyomorpha halys] |
| HhalIR1 | CL2713.Contig2 | 2029 | 620 | Full | 3 | 0 | XP_014277380.1 PREDICTED: glutamate [NMDA] receptor subunit 1 isoform X1 [Halyomorpha halys] |
| HhalIR41c.2 | Unigene1332 | 2264 | 618 | Full | 5 | 0 | XP_014289210.1 PREDICTED: glutamate receptor ionotropic, kainate 5-like [Halyomorpha halys] |
| HhalIR84a | CL1603.Contig1 | 3030 | 603 | Full | 3 | 0 | XP_014275356.1 PREDICTED: glutamate receptor 2-like isoform X1 [Halyomorpha halys] |
| HhalIR75h.1 | Unigene7469 | 2842 | 595 | Full | 3 | 0 | XP_014292011.1 PREDICTED: uncharacterized protein LOC106690927 [Halyomorpha halys] |
| HhalIR75h.2 | CL433.Contig2 | 1909 | 587 | 5' lost | 3 | 0 | XP_014292011.1 PREDICTED: uncharacterized protein LOC106690927 [Halyomorpha halys] |
| HhalIR75d.2 | CL5188.Contig1 | 1864 | 551 | 5' lost | 3 | 0 | XP_014271321.1 PREDICTED: uncharacterized protein LOC106677745 [Halyomorpha halys] |
| HhalIR76b.1 | Unigene6065 | 2386 | 540 | 5' lost | 4 | 0 | XP_014278031.1 PREDICTED: glutamate receptor ionotropic, delta-2 [Halyomorpha halys] |
| HhalIR75d.1 | CL4544.Contig4 | 1838 | 510 | Full | 3 | 0 | XP_014273860.1 PREDICTED: glutamate receptor ionotropic, kainate 4-like [Halyomorpha halys] |
| HhalIR93a | Unigene8291 | 1592 | 482 | 5' lost | 4 | 0 | XP_014280554.1 PREDICTED: glutamate receptor ionotropic, delta-1 [Halyomorpha halys] |
| HhalIR2 | Unigene8045 | 1925 | 438 | 5' lost | 3 | 0 | XP_014293095.1 PREDICTED: uncharacterized protein LOC106691745 isoform X1 [Halyomorpha halys] |
| HhalIR68a | CL4036.Contig2 | 2837 | 403 | Full | 1 | 0 | XP_014283158.1 PREDICTED: glutamate receptor ionotropic, delta-2 [Halyomorpha halys] |
| HhalIR3 | CL5808.Contig1 | 2147 | 398 | 5' lost | 1 | 0 | XP_014279734.1 PREDICTED: uncharacterized protein LOC106683043 [Halyomorpha halys] |
| HhalIR75f | Unigene8336 | 1162 | 361 | 3' lost | 2 | 3.00E-167 | XP_014281565.1 PREDICTED: uncharacterized protein LOC106684160 [Halyomorpha halys] |
| HhalIR4 | CL2713.Contig1 | 1198 | 347 | 5' lost | 1 | 0 | XP_014277380.1 PREDICTED: glutamate [NMDA] receptor subunit 1 isoform X1 [Halyomorpha halys] |
| HhalIR75b.1 | Unigene2454 | 1033 | 344 | 5',3' lost | 2 | 0 | XP_014277125.1 PREDICTED: uncharacterized protein LOC106681363 [Halyomorpha halys] |
| HhalIR21a.1 | Unigene16182 | 1088 | 316 | 5' lost | 2 | 0 | XP_014291746.1 PREDICTED: uncharacterized protein LOC106690730 [Halyomorpha halys] |
| HhalIR21a.2 | Unigene25628 | 546 | 182 | 5',3' lost | 3 | 4.00E-128 | XP_014291746.1 PREDICTED: uncharacterized protein LOC106690730 [Halyomorpha halys] |
| HhalIR60f | Unigene12868 | 469 | 156 | 5',3' lost | 3 | 2.00E-08 | XP_003738808.1 PREDICTED: glutamate receptor ionotropic, kainate 2-like [Galendromus occidentalis] |
| HhalIR5 | Unigene20250 | 375 | 124 | 5',3' lost | 2 | 1.00E-68 | XP_014279731.1 PREDICTED: probable glutamate receptor [Halyomorpha halys] |
