# Supplementary material for: Identification of Candidate Olfactory Genes in the Antennal Transcriptome of the Stink Bug Halyomorpha halys
Source: Front Physiol. 2020 Jul 24;11:876. doi: 10.3389/fphys.2020.00876 (PMC7394822; doi:10.3389/fphys.2020.00876)
Supplement: TABLE S5 — Unigenes of candidate gustatory receptors in Halyomorpha halys. [file Table_5.DOCX]

Table S5. Unigenes of candidate gustatory receptors in *Halyomorpha halys*

| **Name** | **Unigene reference** | **length**  **(nt)** | **ORF**  **(aa)** | **Status** | **TMD**  **(No)** | **E_value** | **Best blastx hit** |
| --- | --- | --- | --- | --- | --- | --- | --- |
| HhalGR1 | CL296.Contig1 | 1656 | 445 | Full | 6 | 0 | XP_014277282.1 PREDICTED: gustatory receptor for sugar taste 64f-like isoform X1 [Halyomorpha halys] |
| HhalGR2 | CL296.Contig2 | 1638 | 439 | Full | 6 | 0 | XP_014277283.1 PREDICTED: gustatory receptor for sugar taste 64f-like isoform X2 [Halyomorpha halys] |
| HhalGR3 | CL5408.Contig1 | 1264 | 411 | 3' lost | 6 | 8.00E-120 | XP_014281823.1 PREDICTED: uncharacterized protein LOC106684328 isoform X1 [Halyomorpha halys] |
| HhalGR4 | Unigene14260 | 1264 | 408 | 5' lost | 7 | 0 | XP_014282852.1 PREDICTED: gustatory receptor 68a-like [Halyomorpha halys] |
| HhalGR5 | CL4278.Contig2 | 1610 | 395 | Full | 7 | 0 | APX56326.1 gustatory and odorant receptor 24-like protein isoform X1 [Halyomorpha halys] |
| HhalGR6 | Unigene16078 | 1588 | 382 | Full | 7 | 0 | XP_014279286.1 PREDICTED: putative gustatory receptor 22f [Halyomorpha halys] |
| HhalGR7 | CL1071.Contig3 | 1644 | 360 | 5' lost | 8 | 2.00E-120 | XP_014285045.1 PREDICTED: gustatory receptor for bitter taste 66a-like isoform X4 [Halyomorpha halys] |
| HhalGR8 | CL1071.Contig2 | 1505 | 349 | 5' lost | 7 | 0 | XP_014285044.1 PREDICTED: gustatory receptor for bitter taste 66a-like isoform X3 [Halyomorpha halys] |
| HhalGR9 | CL1071.Contig4 | 1604 | 346 | 5' lost | 7 | 0 | XP_014285043.1 PREDICTED: uncharacterized protein LOC106686328 isoform X2 [Halyomorpha halys] |
| HhalGR10 | CL4278.Contig3 | 1178 | 318 | 5' lost | 5 | 0 | XP_014271842.1 PREDICTED: gustatory and odorant receptor 22-like isoform X2 [Halyomorpha halys] |
| HhalGR11 | CL2302.Contig2 | 1045 | 318 | 5' lost | 5 | 0 | XP_014271840.1 PREDICTED: gustatory and odorant receptor 24-like isoform X4 [Halyomorpha halys] |
| HhalGR12 | CL5408.Contig2 | 874 | 267 | 5' lost | 4 | 5E-133 | XP_014281823.1 PREDICTED: uncharacterized protein LOC106684328 isoform X1 [Halyomorpha halys] |
| HhalGR13 | Unigene16855 | 493 | 163 | 5',3' lost | 3 | 7.00E-93 | XP_014281153.1 PREDICTED: gustatory and odorant receptor 63a-like [Halyomorpha halys] |
| HhalGR14 | CL1071.Contig1 | 982 | 139 | 5' lost | 3 | 2.00E-88 | XP_014285041.1 PREDICTED: uncharacterized protein LOC106686328 isoform X1 [Halyomorpha halys] |
| HhalGR15 | CL5247.Contig1 | 372 | 111 | 5' lost | 1 | 1.00E-23 | XP_014287605.1 PREDICTED: putative gustatory receptor 28a isoform X1 [Halyomorpha halys] |
