# Supplementary material for: Identification of Candidate Olfactory Genes in the Antennal Transcriptome of the Stink Bug Halyomorpha halys
Source: Front Physiol. 2020 Jul 24;11:876. doi: 10.3389/fphys.2020.00876 (PMC7394822; doi:10.3389/fphys.2020.00876)
Supplement: TABLE S6 — Unigenes of candidate odorant-binding proteins in Halyomorpha halys. [file Table_6.DOCX]

Table S6. Unigenes of candidate odorant binding proteins in *Halyomorpha halys*

| **Name** | **Unigene reference** | **length**  **(nt)** | **ORF**  **(aa)** | **Status** | **Singal peptide** | **E_value** | **Best blastx hit** |
| --- | --- | --- | --- | --- | --- | --- | --- |
| HhalOBP1 | Unigene9567 | 756 | 169 | 5' lost | Y | 7.00E-104 | AOV87018.1 odorant-binding protein 1 [Halyomorpha halys] |
| HhalOBP2 | Unigene17658 | 883 | 175 | Full | Y | 1.00E-101 | AOV87019.1 odorant-binding protein 2 [Halyomorpha halys] |
| HhalOBP3 | CL4954.Contig2 | 1930 | 208 | Full | Y | 2.00E-124 | AOV87020.1 odorant-binding protein 3 [Halyomorpha halys] |
| HhalOBP4 | CL5177.Contig1 | 1635 | 212 | Full | Y | 3.00E-108 | AOV87021.1 odorant-binding protein 4 [Halyomorpha halys] |
| HhalOBP5 | Unigene13248 | 1595 | 333 | Full | Y | 0.00E+00 | AOV87022.1 odorant-binding protein 5 [Halyomorpha halys] |
| HhalOBP6 | Unigene15136 | 581 | 140 | Full | Y | 3.00E-88 | AOV87023.1 odorant-binding protein 6 [Halyomorpha halys] |
| HhalOBP7 | Unigene9642 | 616 | 141 | Full | Y | 2.00E-94 | AOV87024.1 odorant-binding protein 7 [Halyomorpha halys] |
| HhalOBP8 | Unigene8391 | 978 | 143 | Full | Y | 7.00E-97 | AOV87025.1 odorant-binding protein 8 [Halyomorpha halys] |
| HhalOBP9 | Unigene9531 | 874 | 204 | Full | Y | 1.00E-143 | AOV87026.1 odorant-binding protein 9 [Halyomorpha halys] |
| HhalOBP10 | Unigene7592 | 753 | 198 | Full | Y | 1.00E-139 | AOV87027.1 odorant-binding protein 10 [Halyomorpha halys] |
| HhalOBP11 | Unigene3757 | 602 | 139 | Full | Y | 1.00E-96 | AOV87028.1 odorant-binding protein 11 [Halyomorpha halys] |
| HhalOBP12 | Unigene27447 | 648 | 135 | 5' lost | N | 3.00E-73 | AOV87029.1 odorant-binding protein 12 [Halyomorpha halys] |
| HhalOBP13 | Unigene5910 | 722 | 190 | Full | Y | 5.00E-125 | AOV87030.1 odorant-binding protein 13 [Halyomorpha halys] |
| HhalOBP14 | Unigene15555 | 589 | 148 | Full | Y | 4.00E-80 | AOV87031.1 odorant-binding protein 14 [Halyomorpha halys] |
| HhalOBP15 | Unigene19648 | 950 | 178 | 5' lost | N | 2.00E-115 | AOV87032.1 odorant-binding protein 15 [Halyomorpha halys] |
| HhalOBP16 | Unigene1787 | 1350 | 177 | Full | Y | 8.00E-113 | AOV87033.1 odorant-binding protein 16 [Halyomorpha halys] |
| HhalOBP17 | Unigene5571 | 675 | 150 | Full | Y | 3.00E-89 | AOV87034.1 odorant-binding protein 17 [Halyomorpha halys] |
| HhalOBP18 | Unigene1602 | 882 | 241 | Full | Y | 2.00E-104 | AOV87035.1 odorant-binding protein 18 [Halyomorpha halys] |
| HhalOBP19 | Unigene13425 | 667 | 140 | Full | Y | 2.00E-95 | AOV87036.1 odorant-binding protein 19 [Halyomorpha halys] |
| HhalOBP20 | Unigene13949 | 2135 | 138 | Full | Y | 2.00E-77 | AOV87037.1 odorant-binding protein 20 [Halyomorpha halys] |
| HhalOBP21 | Unigene17607 | 1059 | 150 | Full | Y | 4.00E-103 | AOV87038.1 odorant-binding protein 21 [Halyomorpha halys] |
| HhalOBP22 | Unigene13484 | 778 | 167 | Full | N | 9.00E-84 | AOV87039.1 odorant-binding protein 22 [Halyomorpha halys] |
| HhalOBP23 | Unigene16127 | 1196 | 150 | Full | Y | 2.00E-87 | AOV87040.1 odorant-binding protein 23 [Halyomorpha halys] |
| HhalOBP24 | Unigene9512 | 741 | 145 | Full | Y | 4.00E-99 | AOV87041.1 odorant-binding protein 24 [Halyomorpha halys] |
| HhalOBP25 | Unigene9508 | 616 | 148 | Full | Y | 1.00E-86 | AOV87042.1 odorant-binding protein 25 [Halyomorpha halys] |
| HhalOBP26 | Unigene11601 | 586 | 149 | Full | Y | 7.00E-102 | AOV87043.1 odorant-binding protein 26 [Halyomorpha halys] |
| HhalOBP27 | CL4774.Contig2 | 564 | 148 | Full | Y | 9.00E-103 | AOV87044.1 odorant-binding protein 27 [Halyomorpha halys] |
| HhalOBP28 | Unigene5632 | 864 | 205 | Full | Y | 2.00E-127 | AOV87045.1 odorant-binding protein 28 [Halyomorpha halys] |
| HhalOBP29 | Unigene13774 | 1087 | 237 | Full | Y | 4.00E-175 | AOV87046.1 odorant-binding protein 29 [Halyomorpha halys] |
| HhalOBP30 | Unigene19507 | 572 | 136 | Full | Y | 1.00E-94 | APX56329.1 odorant-binding protein 30 [Halyomorpha halys] |
| HhalOBP31 | Unigene19375 | 815 | 240 | Full | Y | 1.00E-126 | XP_014278067.1 PREDICTED: uncharacterized protein LOC106681971 [Halyomorpha halys] |
| HhalOBP32 | Unigene19651 | 939 | 226 | Full | N | 1.00E-168 | XP_014275899.1 PREDICTED: uncharacterized protein LOC106680593 isoform X3 [Halyomorpha halys] |
| HhalOBP33 | Unigene3771 | 617 | 176 | Full | Y | 2.00E-136 | XP_014277009.1 PREDICTED: uncharacterized protein LOC106681282 isoform X1 [Halyomorpha halys] |
| HhalOBP34 | Unigene9573 | 506 | 149 | Full | Y | 9.00E-105 | XP_014281517.1 PREDICTED: general odorant-binding protein 2-like [Halyomorpha halys] |
| HhalOBP35 | CL4774.Contig1 | 455 | 147 | 5' lost | Y | 3.00E-103 | XP_014282204.1 PREDICTED: uncharacterized protein LOC106684563 isoform X1 [Halyomorpha halys] |
| HhalOBP36 | Unigene1703 | 535 | 144 | 5' lost | N | 4.00E-68 | XP_014288229.1 PREDICTED: uncharacterized protein LOC106688327 [Halyomorpha halys] |
| HhalOBP37 | Unigene15236 | 743 | 138 | Full | Y | 4.00E-93 | XP_014281486.1 PREDICTED: general odorant-binding protein 19d-like [Halyomorpha halys] |
| HhalOBP38 | Unigene7622 | 791 | 135 | Full | Y | 7.00E-94 | XP_014281385.1 PREDICTED: general odorant-binding protein 56a-like [Halyomorpha halys] |
| HhalOBP39 | Unigene17455 | 582 | 128 | Full | Y | 3.00E-24 | XP_014288230.1 PREDICTED: uncharacterized protein LOC106688328 isoform X1 [Halyomorpha halys] |
| HhalOBP40 | CL1078.Contig1 | 485 | 128 | Full | Y | 4.00E-88 | XP_014288235.1 PREDICTED: uncharacterized protein LOC106688335 [Halyomorpha halys] |
| HhalOBP41 | CL1078.Contig2 | 524 | 127 | Full | Y | 9.00E-58 | XP_014288234.1 PREDICTED: uncharacterized protein LOC106688334 [Halyomorpha halys] |
| HhalOBP42 | Unigene46449 | 482 | 126 | Full | Y | 1.00E-63 | XP_014288236.1 PREDICTED: uncharacterized protein LOC106688336 [Halyomorpha halys] |
| HhalOBP43 | CL4317.Contig1 | 385 | 126 | 3' lost | Y | 9.00E-61 | XP_014288237.1 PREDICTED: uncharacterized protein LOC106688337 [Halyomorpha halys] |
| HhalOBP44 | Unigene23097 | 294 | 97 | 5',3' lost | N | 1.00E-64 | XP_014277885.1 PREDICTED: uncharacterized protein LOC106681859 [Halyomorpha halys] |
