# Supplementary material for: Identification of Candidate Olfactory Genes in the Antennal Transcriptome of the Stink Bug Halyomorpha halys
Source: Front Physiol. 2020 Jul 24;11:876. doi: 10.3389/fphys.2020.00876 (PMC7394822; doi:10.3389/fphys.2020.00876)
Supplement: TABLE S7 — Unigenes of candidate chemosensory proteins in Halyomorpha halys. [file Table_7.DOCX]

Table S7. Unigenes of candidate chemosensory proteins in *Halyomorpha halys*

| **Name** | **Unigene reference** | **length**  **(nt)** | **ORF**  **(aa)** | **Status** | **Singal peptide** | **E_value** | **Best blastx hit** |
| --- | --- | --- | --- | --- | --- | --- | --- |
| HhalCSP1 | Unigene11630 | 532 | 135 | Full | Y | 2.00E-46 | AEI71725.1 chemosensory protein 1, partial [Euschistus heros] |
| HhalCSP2 | Unigene5731 | 674 | 131 | Full | Y | 2.00E-54 | AEP95755.1 chemosensory protein 1 [Apolygus lucorum] |
| HhalCSP3 | Unigene1675 | 564 | 130 | Full | Y | 2.00E-38 | SAJ59004.1 putative chemosensory protein [Triatoma brasiliensis] |
| HhalCSP4 | Unigene6325 | 3228 | 128 | Full | Y | 5.00E-33 | SAJ59011.1 putative chemosensory protein [Triatoma brasiliensis] |
| HhalCSP5 | Unigene17537 | 1331 | 128 | Full | Y | 5.00E-25 | SAJ59008.1 putative chemosensory protein [Triatoma brasiliensis] |
| HhalCSP6 | Unigene20305 | 1995 | 128 | Full | Y | 2.00E-33 | SAJ59011.1 putative chemosensory protein [Triatoma brasiliensis] |
| HhalCSP7 | Unigene1672 | 581 | 126 | Full | Y | 2.00E-41 | ACZ58019.1 chemosensory protein 1 [Adelphocoris lineolatus] |
| HhalCSP8 | Unigene13515 | 1588 | 126 | Full | Y | 4.00E-51 | SAJ59003.1 putative chemosensory protein [Triatoma brasiliensis] |
| HhalCSP9 | Unigene15319 | 2601 | 126 | Full | Y | 3.00E-41 | SAJ59006.1 putative chemosensory protein [Triatoma brasiliensis] |
| HhalCSP10 | Unigene17438 | 501 | 126 | Full | Y | 1.00E-67 | AGD80083.1 chemosensory protein 3 [Apolygus lucorum] |
| HhalCSP11 | Unigene3812 | 1366 | 122 | 5' lost | N | 2.00E-49 | APB88037.1 putative chemosensory protein 1 [Lygus hesperus] |
| HhalCSP12 | Unigene17428 | 812 | 121 | Full | Y | 3.00E-45 | AQS80468.1 chemosensory protein 8, partial [Bemisia tabaci] |
| HhalCSP13 | Unigene4888 | 425 | 120 | Full | Y | 2.00E-40 | SAJ59003.1 putative chemosensory protein [Triatoma brasiliensis] |
| HhalCSP14 | Unigene5589 | 781 | 117 | Full | Y | 2.00E-40 | AQS80468.1 chemosensory protein 8, partial [Bemisia tabaci] |
| HhalCSP15 | Unigene12883 | 528 | 117 | 5' lost | Y | 2.00E-20 | SAJ59003.1 putative chemosensory protein [Triatoma brasiliensis] |
| HhalCSP16 | Unigene15756 | 5953 | 117 | Full | Y | 4.00E-46 | AEI71726.1 chemosensory protein 2, partial [Euschistus heros] |
| HhalCSP17 | Unigene20639 | 797 | 117 | 3' lost | Y | 5.00E-24 | APB88044.1 putative chemosensory protein 8 [Lygus hesperus] |
| HhalCSP18 | Unigene26594 | 904 | 114 | Full | Y | 7.00E-20 | ACZ58026.1 chemosensory protein 8 [Adelphocoris lineolatus] |
