# Supplementary material for: Identification of Candidate Olfactory Genes in the Antennal Transcriptome of the Stink Bug Halyomorpha halys
Source: Front Physiol. 2020 Jul 24;11:876. doi: 10.3389/fphys.2020.00876 (PMC7394822; doi:10.3389/fphys.2020.00876)
Supplement: TABLE S8 — Unigenes of candidate sensory neuron membrane proteins in Halyomorpha halys. [file Table_8.DOCX]

Table S9. Unigenes of candidate sensory neuron membrane proteins in *Halyomorpha halys*

| **Name** | **Unigene reference** | **length**  **(nt)** | **ORF**  **(aa)** | **Status** | **E_value** | **Best blastx hit** |
| --- | --- | --- | --- | --- | --- | --- |
| HhalSNMP1.1 | CL3063.Contig1 | 3510 | 499 | Full | 0 | XP_014284496.1 PREDICTED: sensory neuron membrane protein 1-like [Halyomorpha halys] |
| HhalSNMP1.2 | CL3159.Contig1 | 2994 | 634 | 5' lost | 0 | XP_014291654.1 PREDICTED: sensory neuron membrane protein 1-like, partial [Halyomorpha halys] |
| HhalSNMP2 | Unigene13159 | 2111 | 554 | Full | 0 | XP_014285935.1 PREDICTED: sensory neuron membrane protein 2-like [Halyomorpha halys] |
