# Supplementary material for: Identification of Candidate Olfactory Genes in the Antennal Transcriptome of the Stink Bug Halyomorpha halys
Source: Front Physiol. 2020 Jul 24;11:876. doi: 10.3389/fphys.2020.00876 (PMC7394822; doi:10.3389/fphys.2020.00876)
Supplement: FILE S1 — The amino acid sequences of the chemosensory proteins identified in this study. [file Data_Sheet_1.PDF]

Supplementary material 1

HhalActin

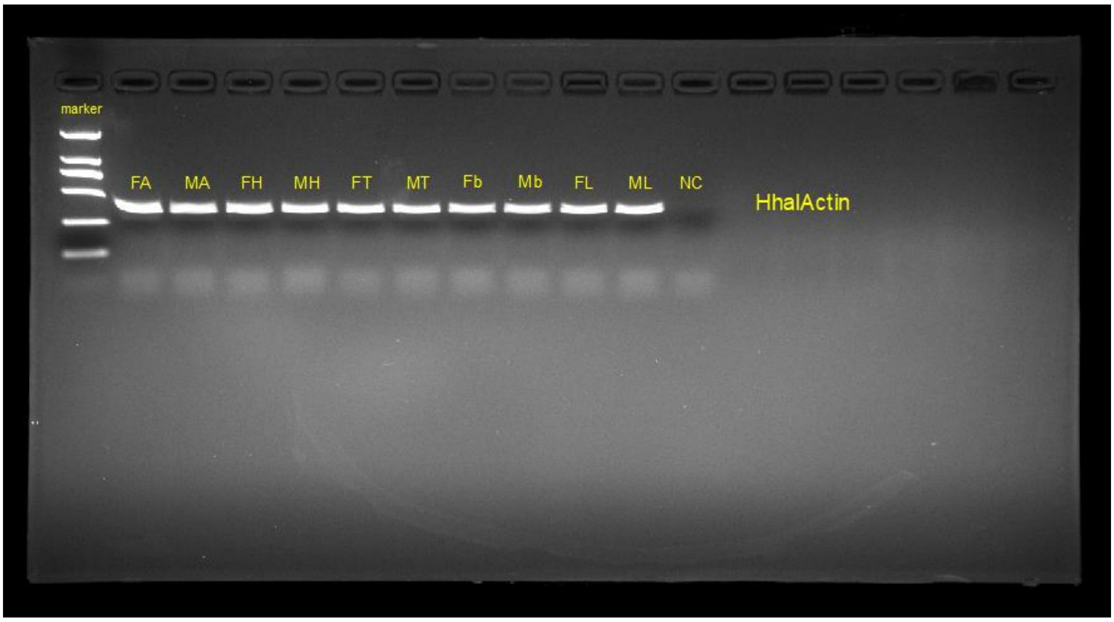

HhalOBP1, 26, 40, 42, 44

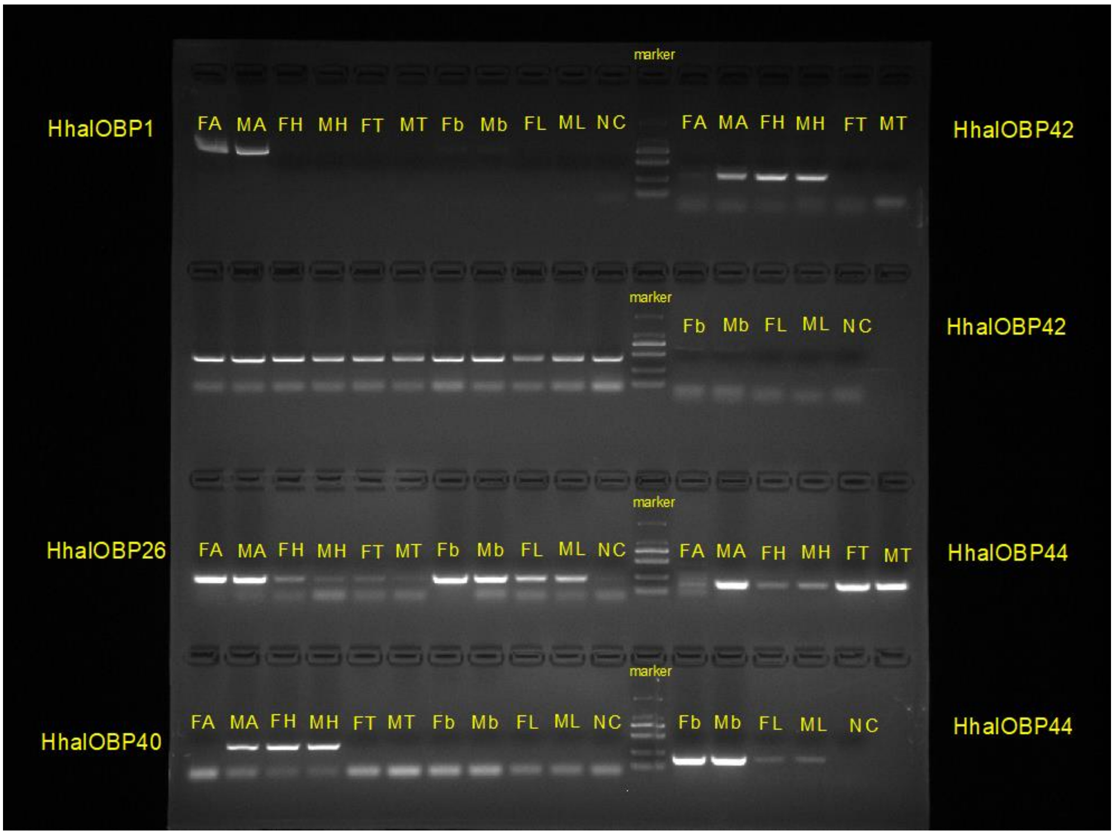

HhalOBP2, 4, 6

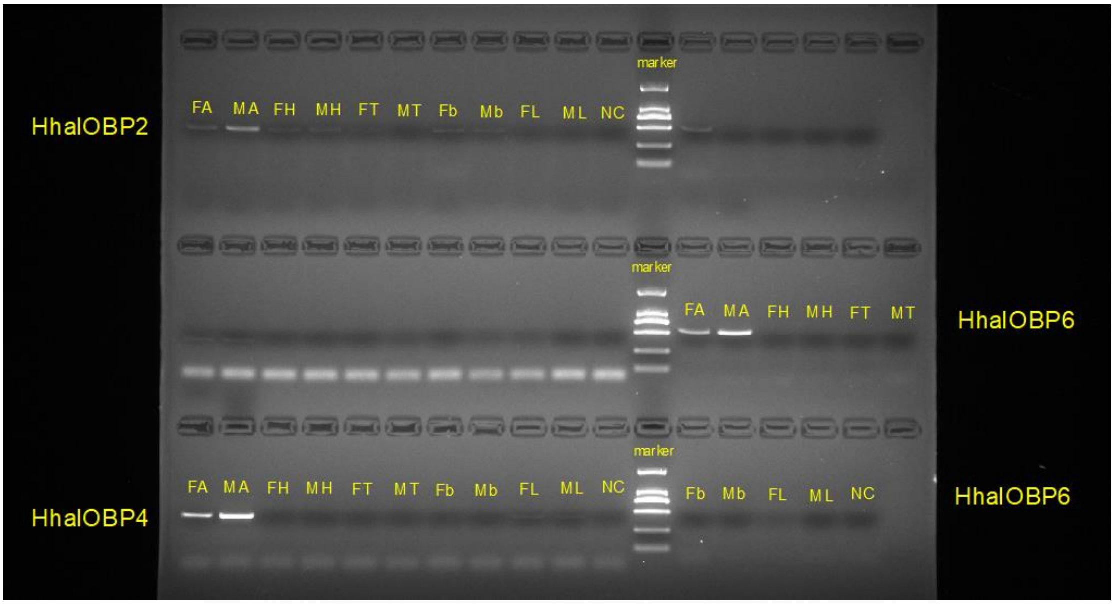

HhalOBP3

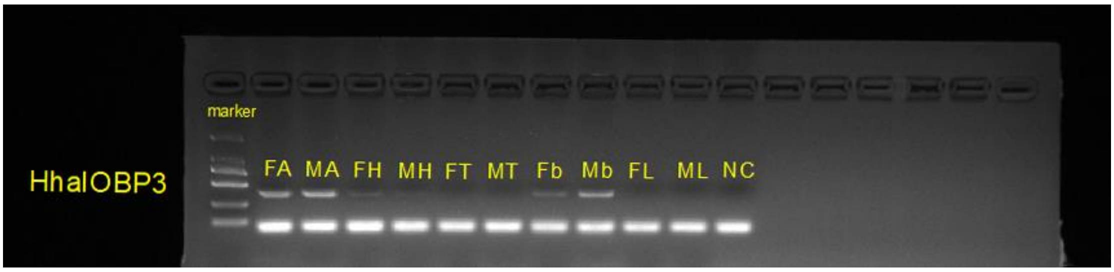

HhalOBP5, 10, 16

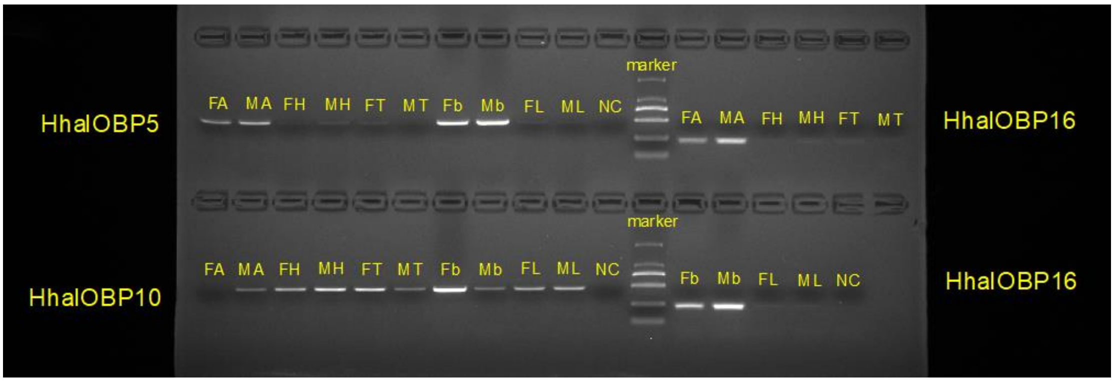

HhalOBP7, 8, 9, 11, 12

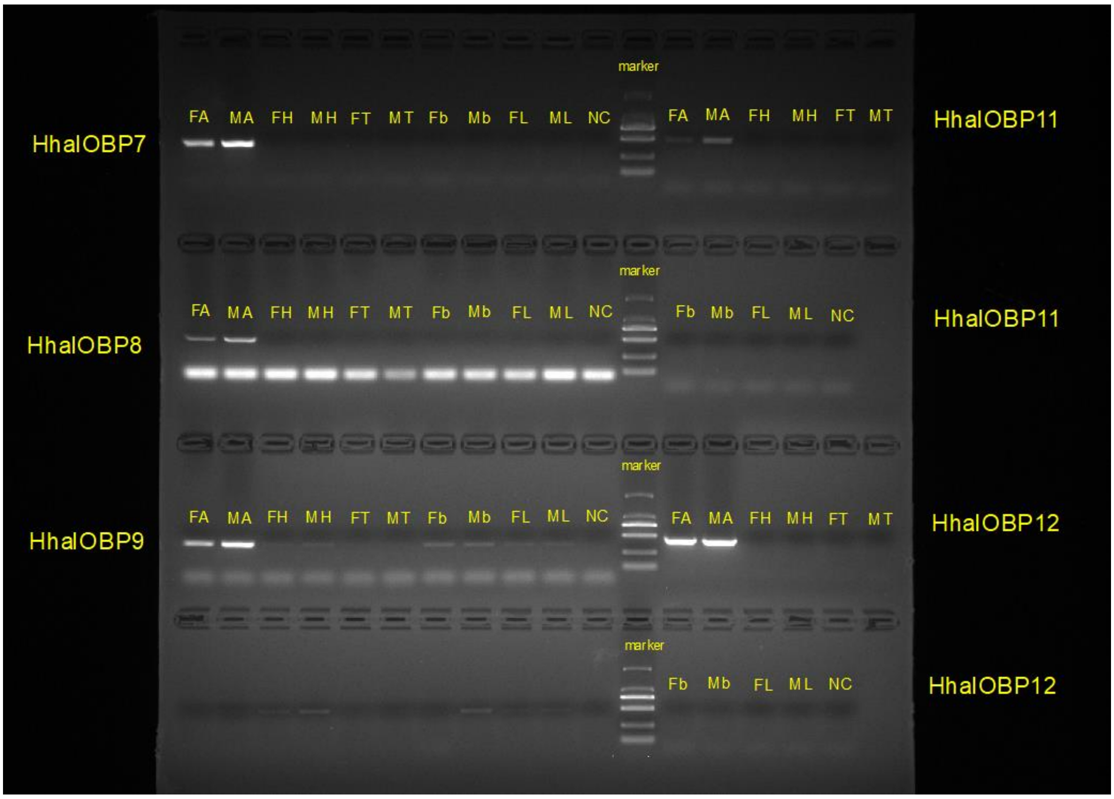

HhalOBP13,

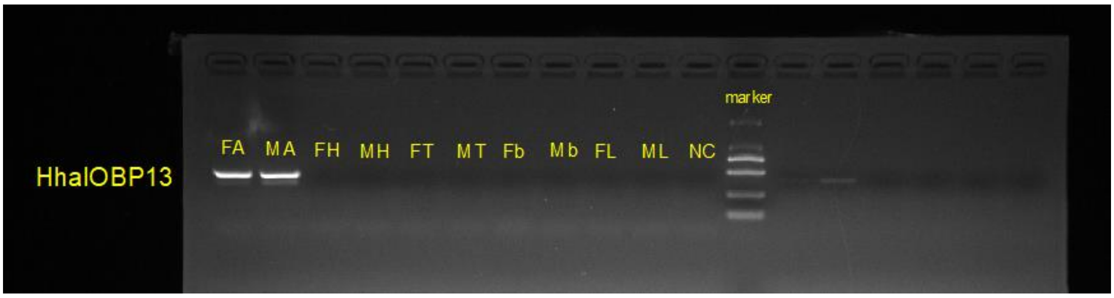

HhalOBP14

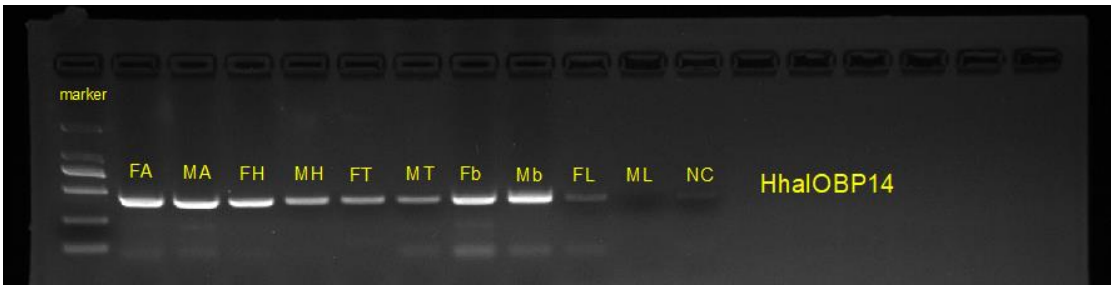

HhalOBP15

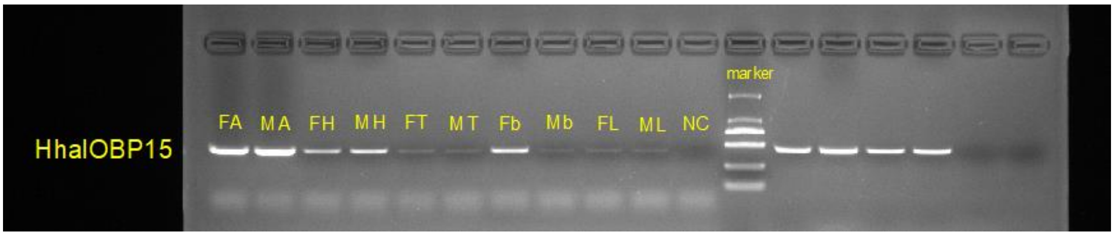

HhalOBP17, 22

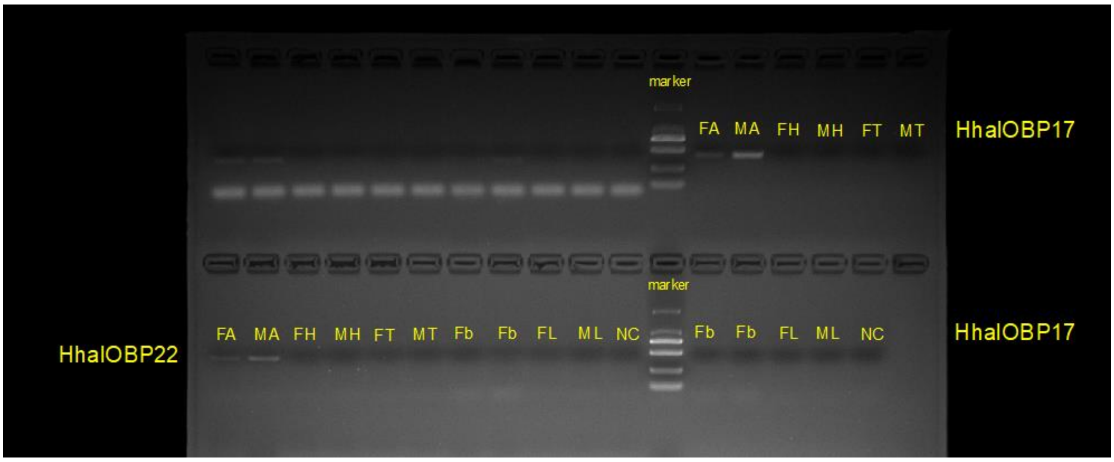

HhalOBP18

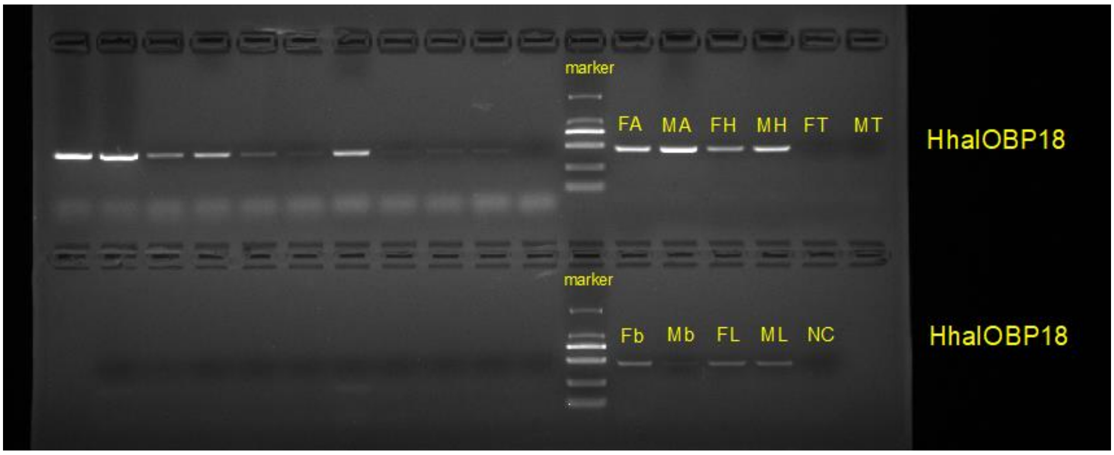

HhalOBP19

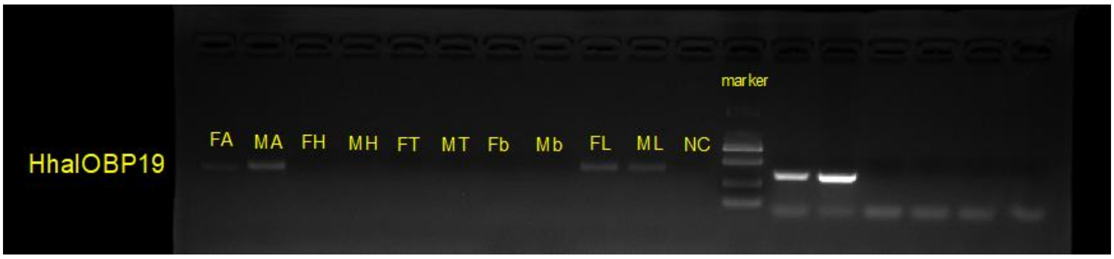

HhalOBP20, 35, 37

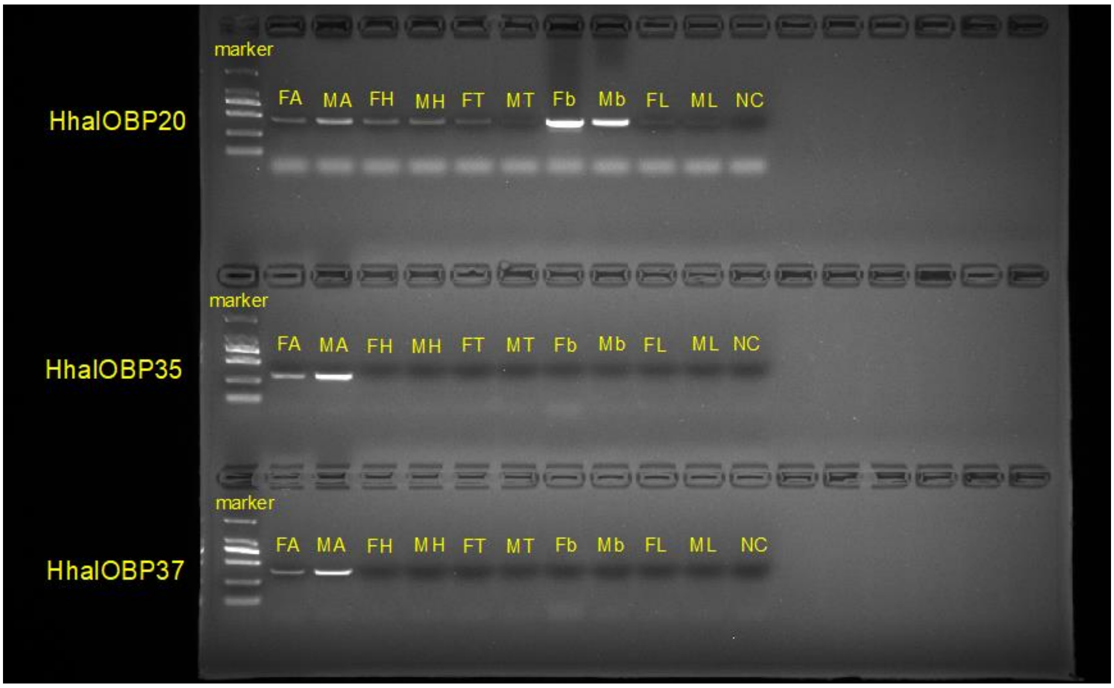

HhalOBP21

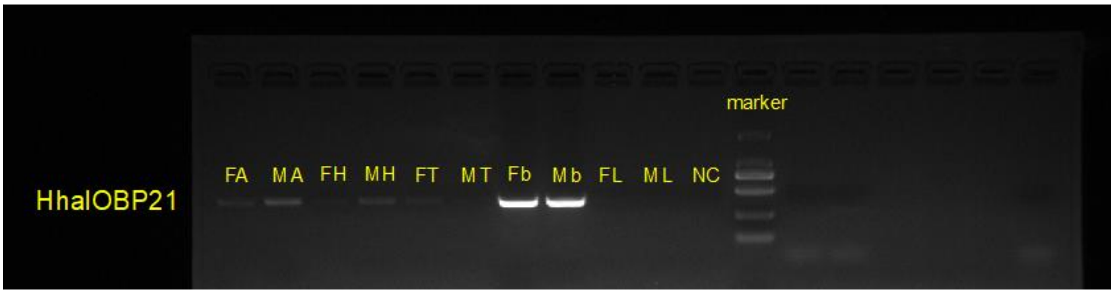

HhalOBP23, 24

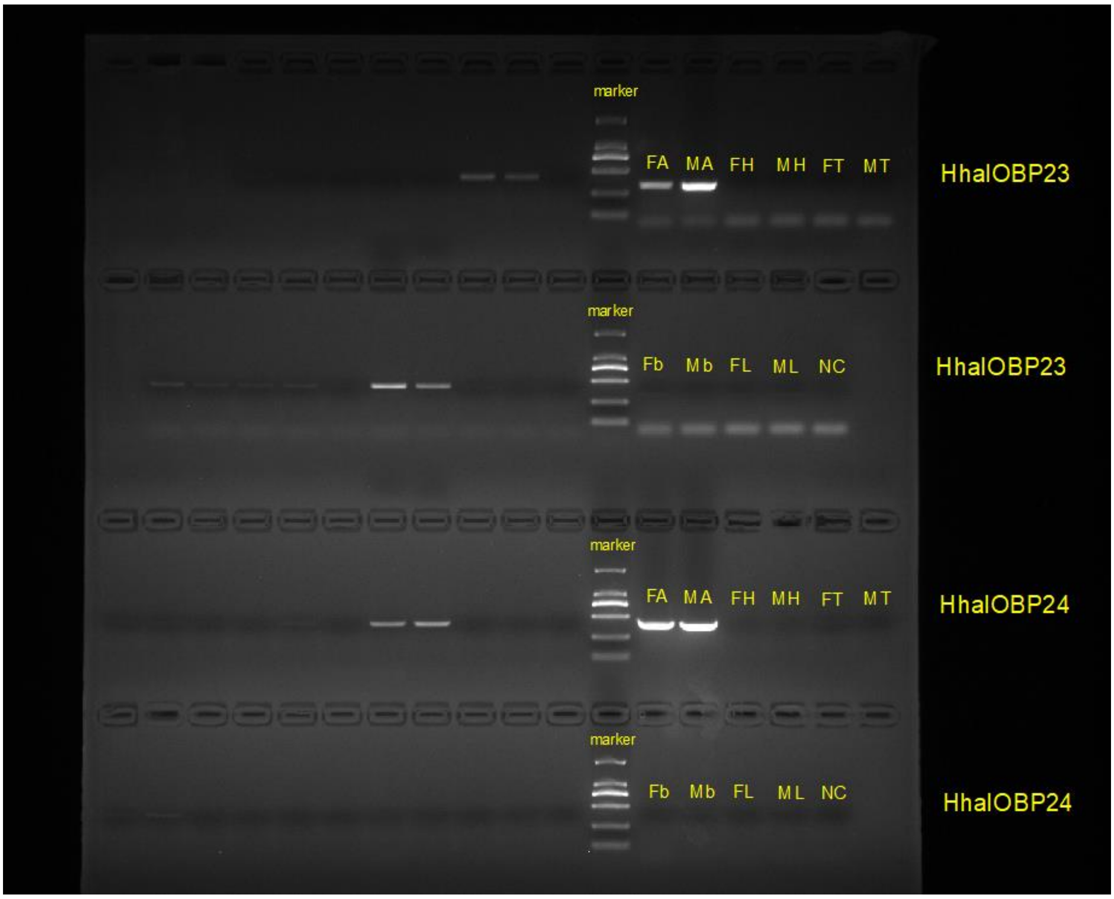

HhalOBP25, 27, 28, 29, 30

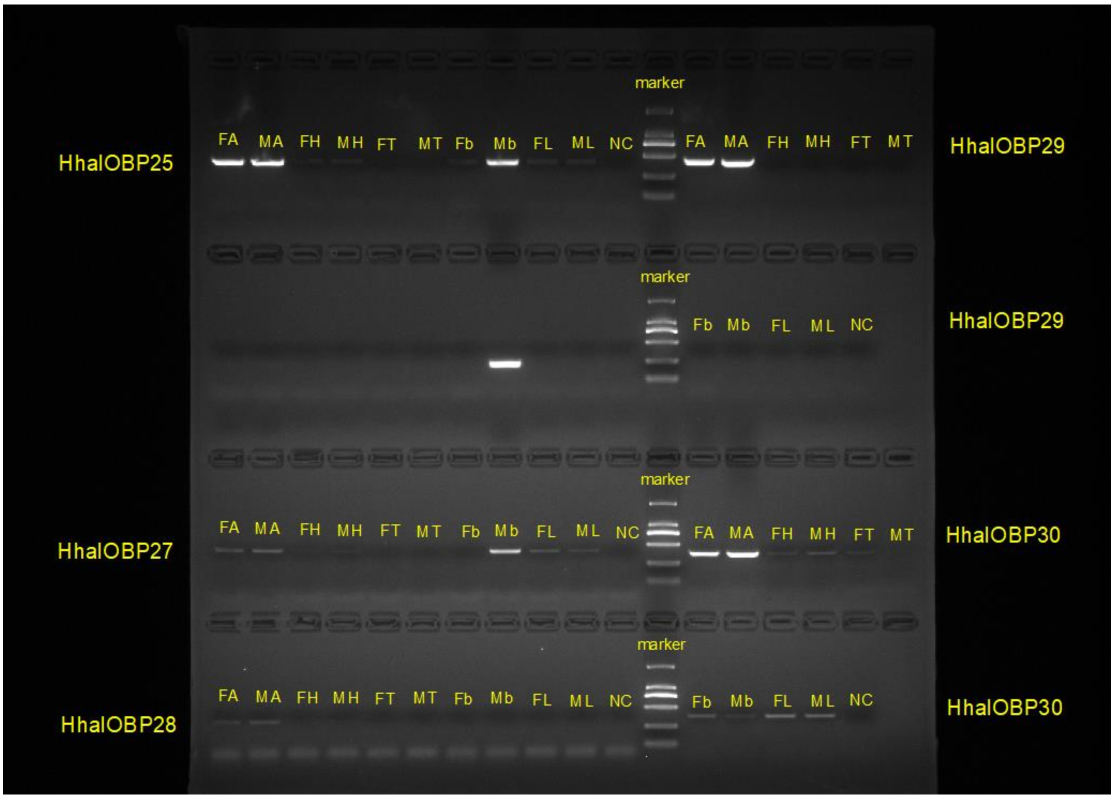

HhalOBP31

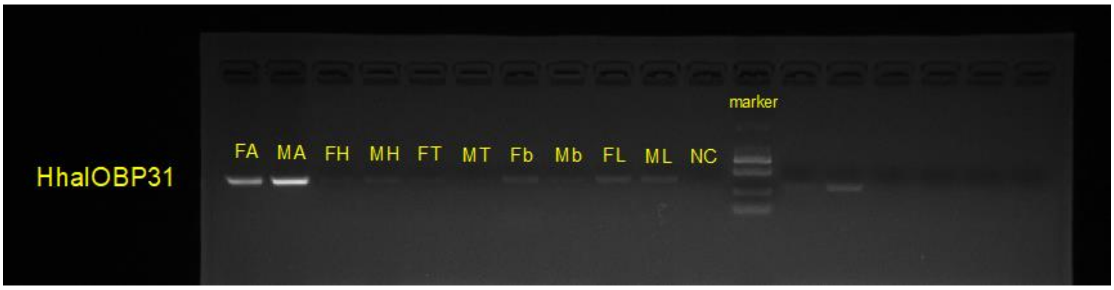

HhalOBP32, 33, 34, 36

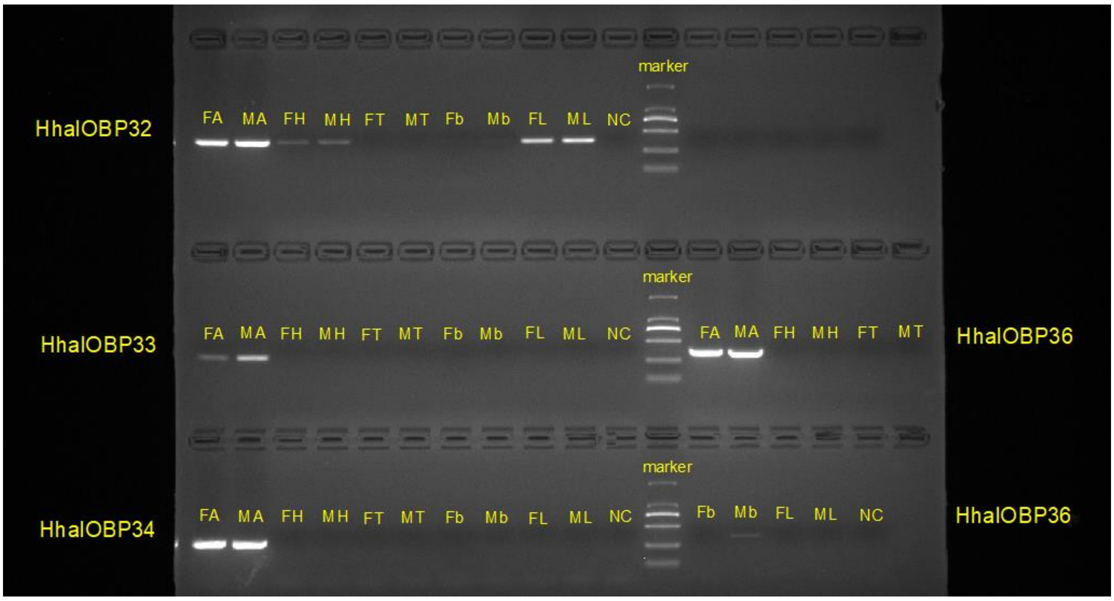

HhalOBP38

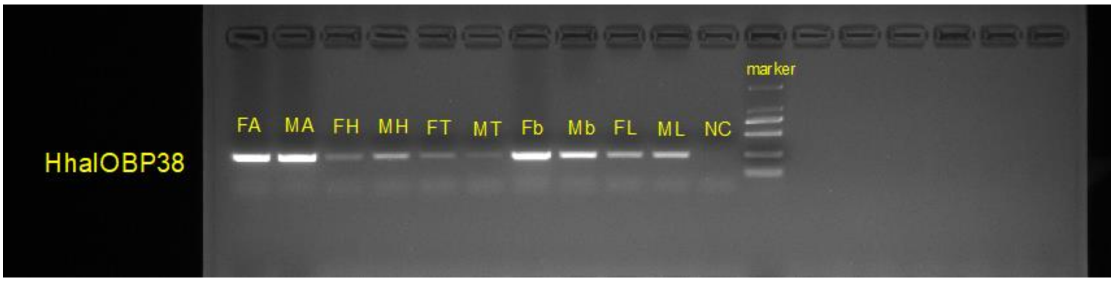

HhalOBP39

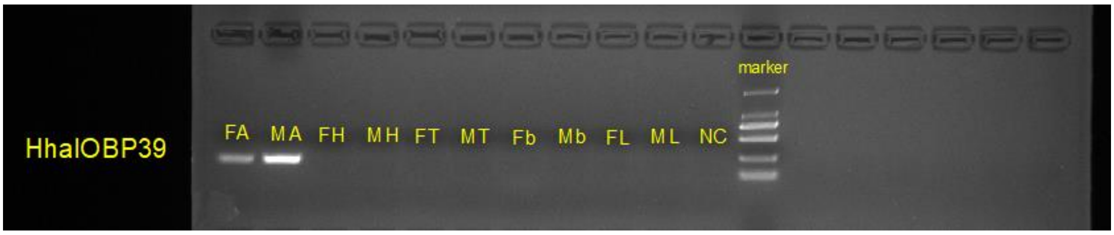

HhalOBP41

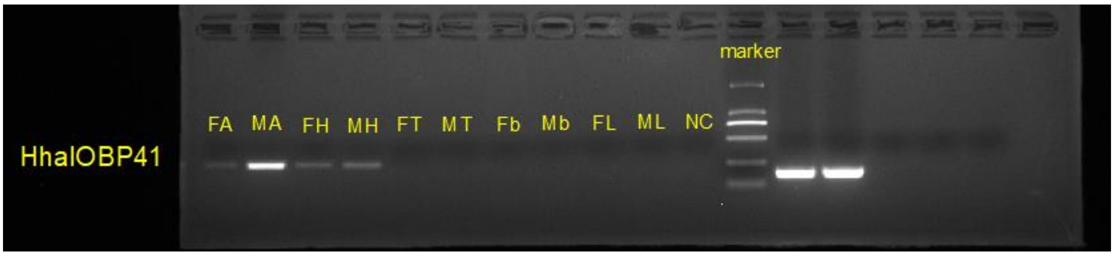

HhalOBP43

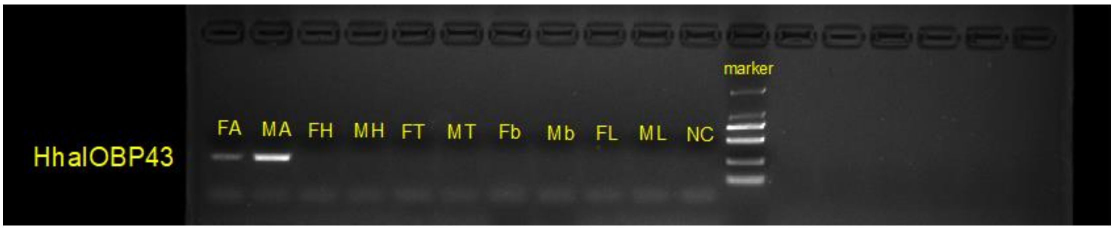

Supplementary material 1. The original gels of the cropped images of blots used in the figure 6. FA: female antennae, MA: male antennae, FH: female head, MH: male head, FT: female thoraces, MT: male thoraces, Fb: female belly, Mb: male belly, FL: female legs, ML: male legs, NC: no template control. HhalActin was used as a reference gene for each cDNA template.
