# Supplementary material for: Identification of Candidate Olfactory Genes in the Antennal Transcriptome of the Stink Bug Halyomorpha halys
Source: Front Physiol. 2020 Jul 24;11:876. doi: 10.3389/fphys.2020.00876 (PMC7394822; doi:10.3389/fphys.2020.00876)
Supplement: Supplementary file 12 [file Data_Sheet_2.PDF]

Supplementary material 2

HhalActin

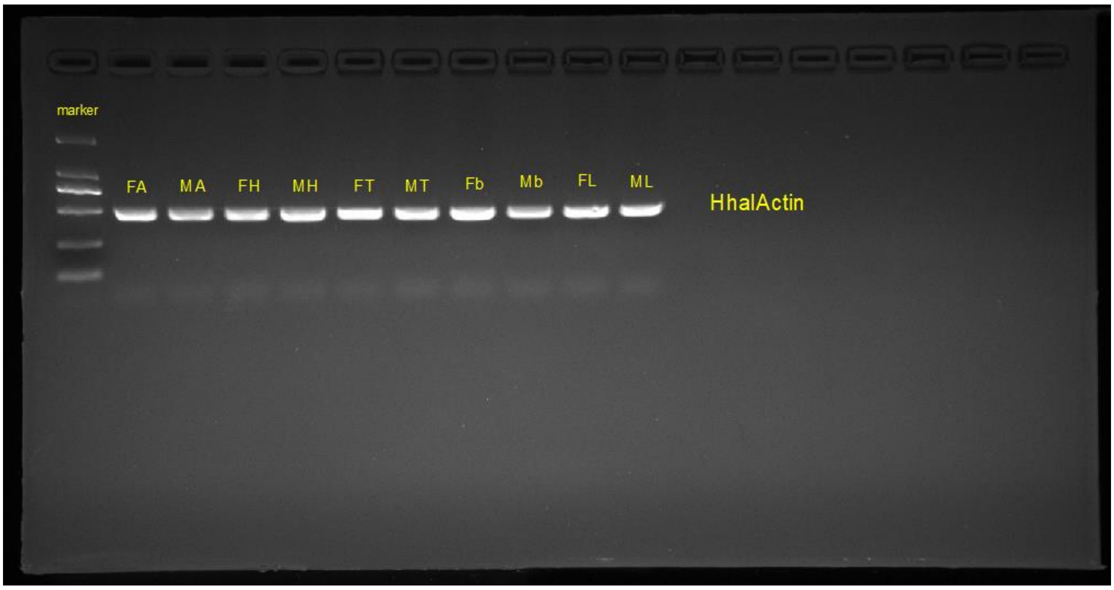

HhalCSP1, 2, 3

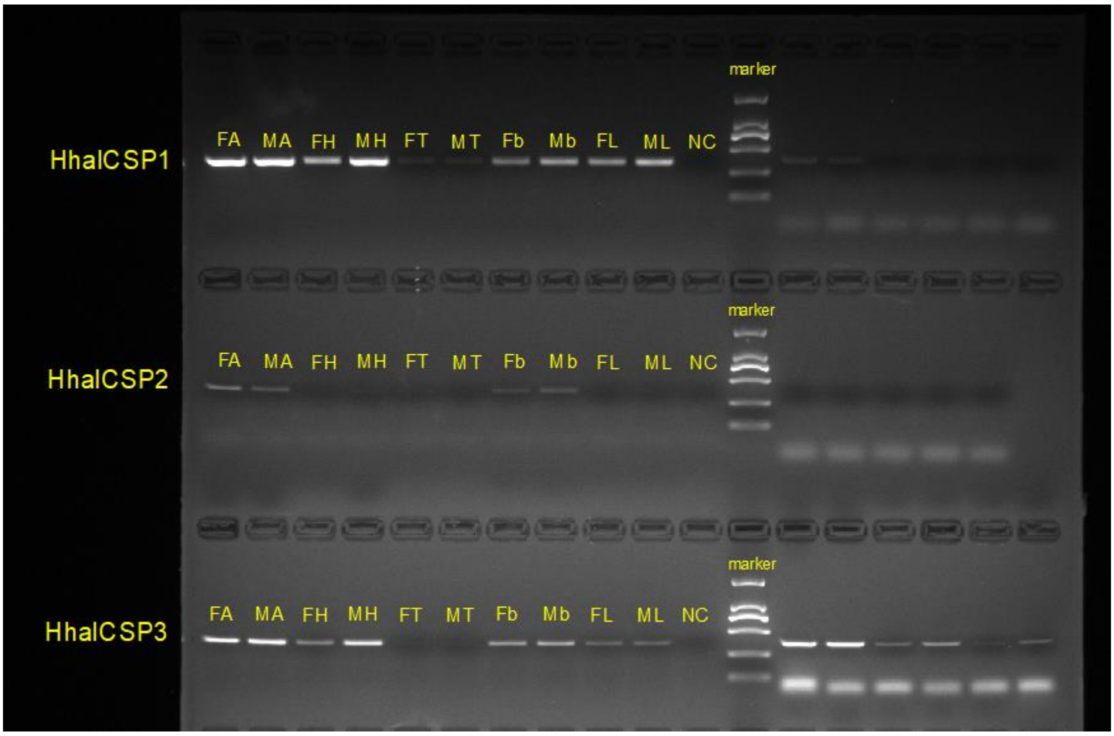

HhalCSP4

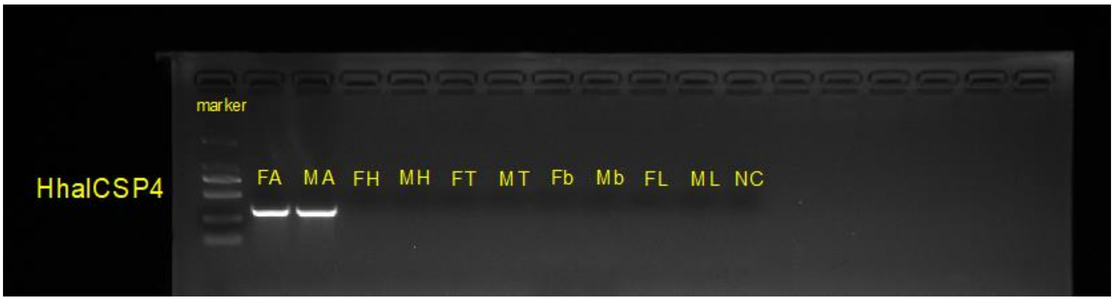

HhalCSP5

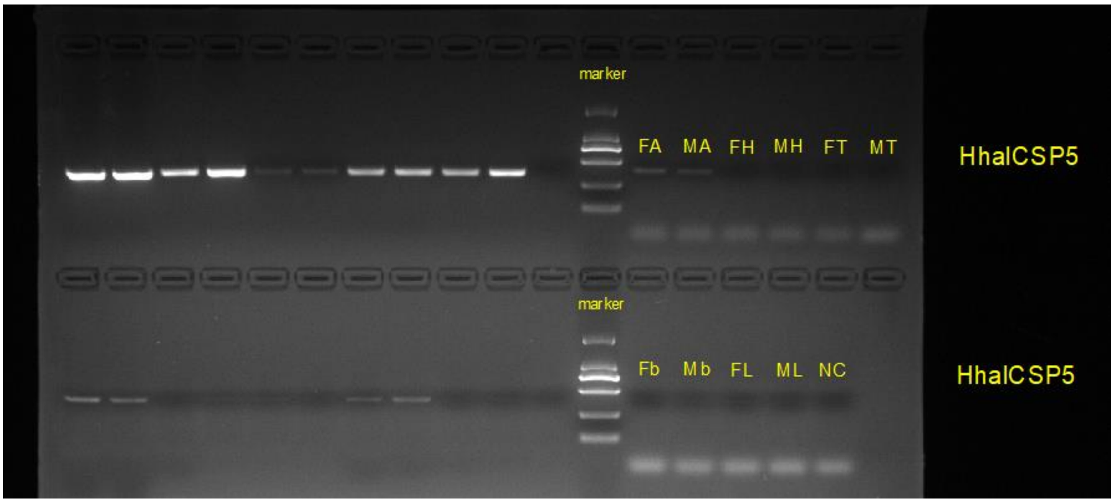

HhalCSP6

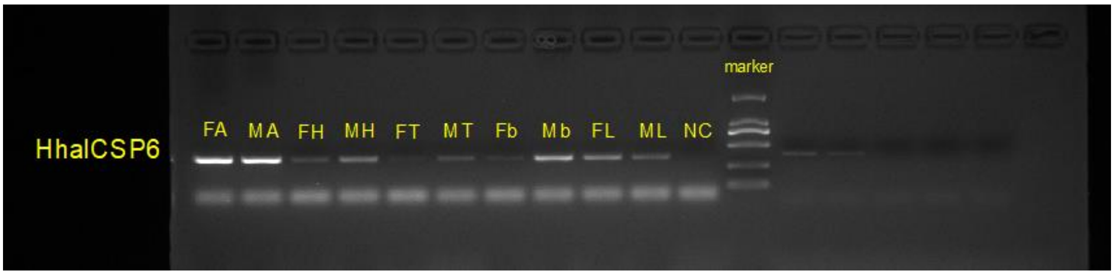

HhalCSP7, 8

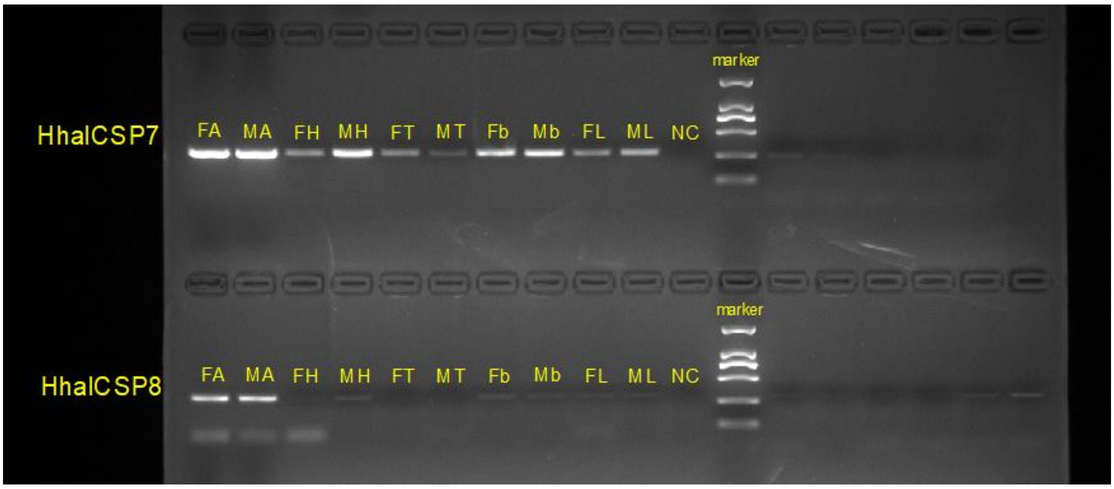

HhalCSP9, 12

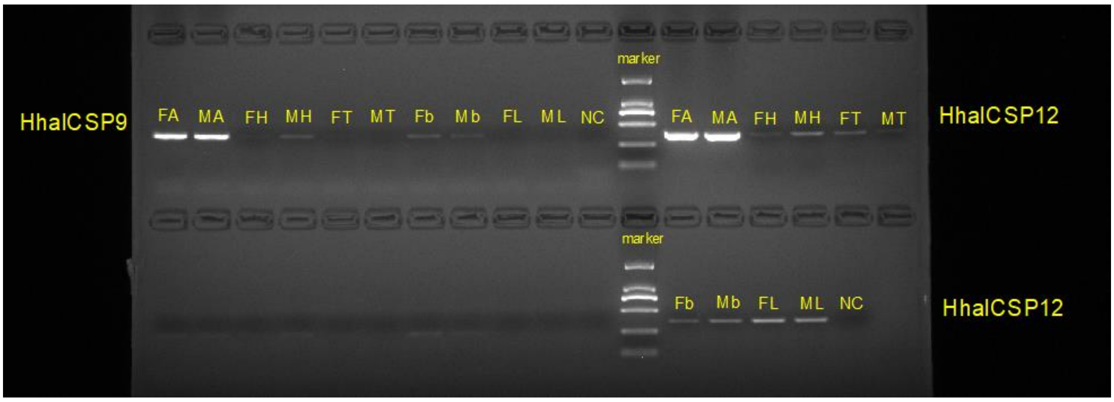

HhalCSP10, 11

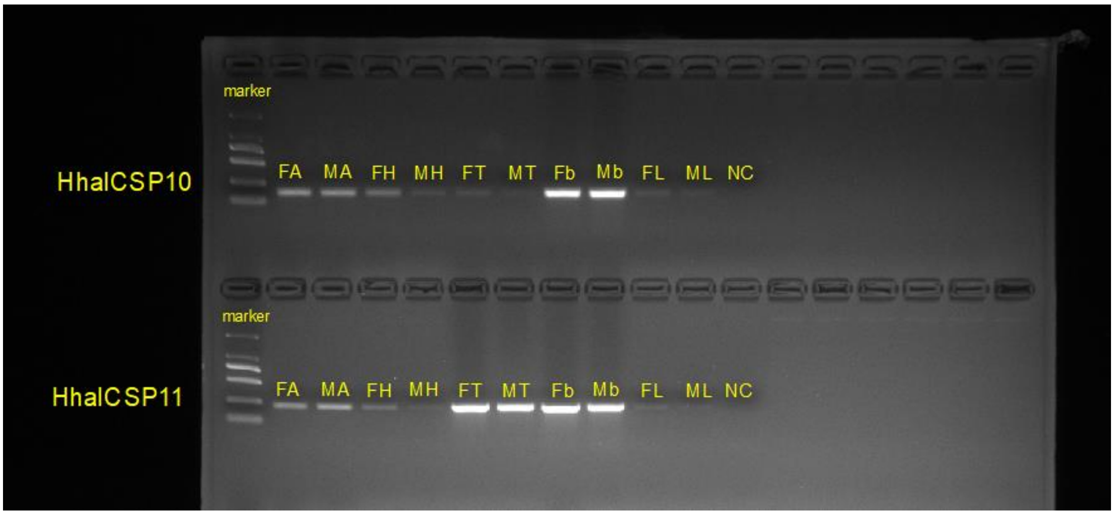

HhalCSP14

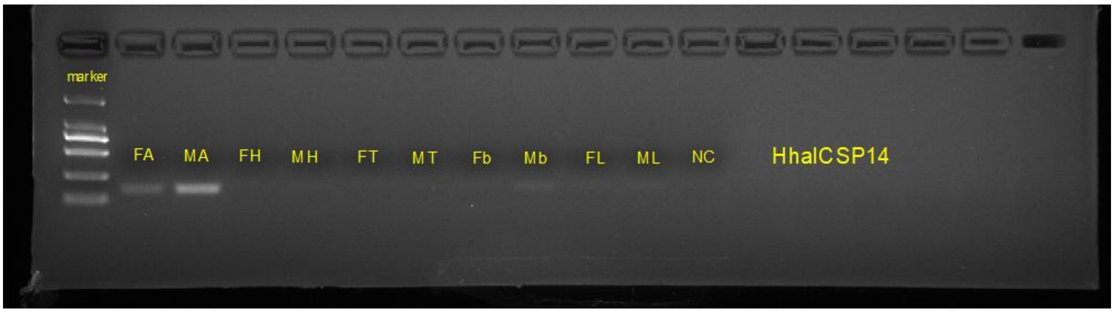

HhalCSP15, 16, 17

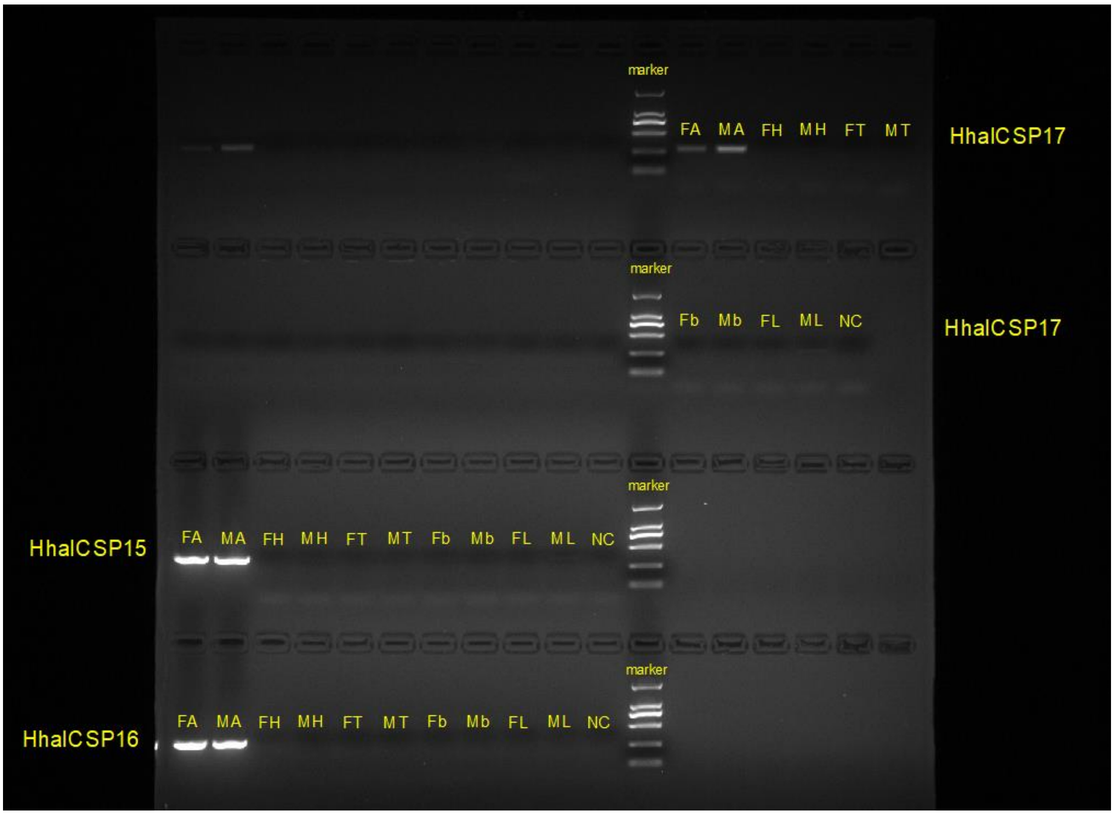

Supplementary material 2. The original gels of the cropped images of blots used in the figure 7. FA: female antennae, MA: male antennae, FH: female head, MH: male head, FT: female thoraces, MT: male thoraces, Fb: female belly, Mb: male belly, FL: female legs, ML: male legs, NC: no template control. HhalActin was used as a reference gene for each cDNA template.
