# Supplementary material for: Identification of Candidate Olfactory Genes in the Antennal Transcriptome of the Stink Bug Halyomorpha halys
Source: Front Physiol. 2020 Jul 24;11:876. doi: 10.3389/fphys.2020.00876 (PMC7394822; doi:10.3389/fphys.2020.00876)
Supplement: Supplementary file 13 [file Data_Sheet_3.DOCX]

>HhalOrco

MQKIKMHGLVGDLWPNIRLMQLTGHWLLEYHEETGGMVRLIRLAYCWLTTFLVVMQFAFLACFLILDTYDADQMAAATITTLFFLHSITKFAYFAIRSKYFYRTFGAWNQVNSHPLFAESNARHRATALSRMRKLLMIIGVITIMSVMAWTTVTFLGDPHREITDPEDVNSTITVEMPQLMVDAWYPWDAKTGFCFFATFIYQLYWLFISLSHANLLDILFCSFVIFACEQLKHLKEILQPLMELSATLDSVVPNSGELFRGGSGGSNMPLVENDGNDFDIRGIYSNRGDFSGFGQTAVSTIQTNGNGIGPNGLTKKQELLVRSAIKYWVERHKHVVKFVSSIGDAYGSALLLHMLTSTVTLTLLAYQATKIEGVDVYASTVIGYLLYTLGQVFVFCIHGNELIEESSSVMEAAYSCHWYDGSEEAKTFVQIVCQQCQKSLTVSGAKFFTVSLDLFASVLGAVVTYFMVLVQLK

>HhalOR1

YPPLQDSNIIDGLRVGYLKFFGFWKIINDYRTTGKKNTFMIFKFYTTFIISTPYIVPQLFSYFAINMDIEKATIINLHCLPAIMMCGRILVFWFRMDSQCKLYNLIQKDFLHIPEYKMAKASIIYRKITKNANLLCILAFVMDFSVAFAIASIPGVPVDYILYHKGSMFDVKTGRKKILSGWYPLPMAESPYYEIIFAYEMVCVLTGGLFQPIYICLFYQVLVALIAQFEVLGYHVSTFNISPNSDISQINYNPSAECSIIDSDSYNQNNINSNSNISHRKKITSEKSDSDISEDLYRILQDHQTLLSYADELRSVYNPLVTMNLGLAICFLIVSVFQYQSGETRDIVFVIKALLFMAAQMIELFMFCFGSSSLQAASSDLQLAVYSSDWYKADVKLRKTAQMLMVGAKKGVTLTAIKMYPVNKETLMSIFQFAYSTSALMSGMLEK

>HhalOR2

MVFGISPNKWLHDKVKKLNESVDNEIDVYAFIDKEYFRLLQISCIYVKLDKRNRLMSLIQWSFYVTVLFYHYILLTRSTIFMMDINMVLFSQSTHFTLLVQLTTILIVWLQTRHRQITLFHKLLSVDFYDYQEPPAKGADALRNGMVRERRLLSAIPVGAALAAGGVLIIAPIVDRNAGLFDFEEMSKEFSTHMPYPYGKYPYQTIEGISYYLTLGGQLIIGLLLTIIIGSGGFIFLNLAQNVSLQLKLLENSMDQIETRAEFMYLRKCGKTRRDTPSLYNDHQFIYCYNKCLRKNYEHHQVIMRAFHYMEETVSVPMFLAYMTGTIVIALSLISAGSADELPGTTLASMILCGTEIGYMFLFSVFGQRLGDLNMDLRFKIYNIKWYLCNRKVKSNMMIFQERTLKPLTMMAGGIVPANMETFATVMNSAYSYYNLVNAFGN

>HhalOR3

MSPKDKAGYLVEKSNIESDKLTEKFIDSNYYYLTQLSLLYMKMDKKHRIFSIAQFLTFYTIMGYHFILLIRSSVALFGINFIIFVHNSHIGLFSILVPTVVLDFQRHRSAFFHLHRLMFTGFYDYEEPELITVANVREDMTRQRRRLAVIPVSACLATGAVLVLAKFLDRLGTFDFNKTDGLVSDDLPYAFGVYPYNTKSGPGFYISYLLQVMHAAVLAAPIGLSGWTYIVLSQNIMLQLKILIASLESIEERSKQMCERTFPGRFDASSQKTFDSKAFMYCYNICLNKNFEHHQIIMSAFHSLKELFSVPVFLSYMAGTIVIALSLLSSGSGIELPGTTIASALLCFAEVGFMYLFSVFSQHITDLSLDLRFTMYSTKWYYCNKSTAQSIAIFQTMTLKPMTLTAGGIVPANMDTFGKVMNSAYSYYNVVNAFNITV

>HhalOR4

MWPVDKITHMFDAMSAESNDEVDDYVSAQYLHLIQYSAMYTRLTRKHRLFTLLEIIIYEFILIYHLIHLGKAALMLIDVSLVTFGETIHFCLLIQLDIILIIFIQTKHNKIALFHRAMAKNFYDYSESYQNNMEELQQDIRKERMLLAMIPITVVMAVPAVLVLTPQVDQYGTFDFSKISTEFNQQLPFPYIIYPYHTEEGIGYYTAALLQVVLATILGGSIGLGGFAYIVMSQNLWIQMEILYDSVNHIEERAEILRSDLFGAKNKLSGDKLYQQVQFEYCYNICLKKNFMHHQIIMRNFHLWADIFSVPTFLAYLTGTLVIALSLLSLGSWVELPGTTISSLILCAGEVGFMYIFSVFSQRLTDLNEKLRFEIYCIKWYLCNKEVKTSLKIFQEVTLRPMTLIAGGLIPANMDTFSKVMNSSYSYYNLVTAFNVA

>HhalOR5

MWLVDKVTQIVDAMSAESNDEVDDYVSAQYLHLIQFSAMYTKLNRRRRLFTLLQIIIYEFILIYHLILMVKAALMLIDVSLVTFGETTHFCLLIQLDIILVIFIQTKHNKVALFHRYMMKNFYDYSEFYELNKEGLQQNIRKERMLLAMIPVTVAISVPAVLVLTPQVDQYGTFDFSKISGDFNQHLPFPYIKYPYQTTEGMGYYVASLFQVIGACILSGSIGVGGFAYIVMSQNLWMQMEILYDSIKHIEERTEILQFKLFGARNKLSGDKLYYQTQFAHCYNICLNKNFRHHQIIMRNFHLWADIFSVPTFLAYLTGTLVIALSLLSLGSWVELPGTTISSLILCAGEVGFMYIFSVFSQRLTDLNEKLRFEIYCIKWYLCNKEVKTSLKIFQEVTLRPMTLIAGGLIPANMDTFSKVMNSSYSYYNLVTAFNVA

>HhalOR6

MAGMFSQFIDFLNTGGDEKTDLFIEKTFMKLTTVSAIYPILDKQRPLVCLGNFALFLFHSASLTYSWILTAKTSVILFDINFVPFVHEVHLTLLLILSVIILWHSWLKRPNVAKLFRFIDKDLFDYEEELEDKLKLLRSDRIKERRRYILCVIMIGVAASVAVILVPTVNKLGTFNFNATLYEVNFELAVPVTYPFTSMEPVPFFFENAYIMLGATAIALMNCTKSFMAIESNLTLQMHLKLLIYQIENIQVRSAKLYRQLYGVKPKLGGLGSYTPKFLKCYNICLKKTIQHHHVISEVVVEFNEVFGFYVFFYYLTGTLDIAMSMLATSSTDEFPGTTIGAMIICLVEVCFVALFAHMGQRLTDLSVDLREVIYNTPWYRCDQGVANNIKILQIATLKPISFSFYSLLNINYDSFATVMHSAYSYYNLVNAYNK

>HhalOR7

MGVLERLRNFLQDWNRWSDKYSDIMIQKEYRGSMHIIGLYPNVNKVGLFWGSFRALLVGVLLLFHTFGLVVSAVLLYDINFIYFSTALHYVLIVGIAFLYVLYFNTNRHRFARFHTKMYFNFYDYHEEITDEIVDLIKKGEKQKMRFTILPMFAAFIAVCILLVAPILDKYGTFDFDIETTINFNLPLPMVYVFDSSTGAGYFVALTHQLSSAMLCGLVLGGTGYSFIATVSTITIQMRILINRLDTIESRALSLYEANYGLKPNMNDYDLYRDEKFSNCMDTCLRRCVEHHKVILSNISELKDFFSLPLFSIFFVAALIIALAMVGSSNWKVLPGNTIVSLMLCLVEIAFLAIVSSLGQSVTDLNEDIRNSVYGIKWYHCSKGFCLNIRILLSKTLIPVTFTALGLIKICHATYANIINSAYSYYNLASVTSS

>HhalOR8

MGLTTSVEGLLRKWNDASDEYSDIVMQREYRNYVHIIGLYPNINKVGYFWSCFKIMIIGASLLFHSIGLVVSTVLLYDINFIYFSSALHYVFVVGISFFYNFHFNKNRKFLARYHSRMYFNFYDYKEETTDEIRELVKSTEVQKKRFMILPIFSACAAVCILLLAPILDKYGTFDFDIETSINFNLPLPMVYVFDTFTVAGYLVALTHQLLSAMLCGLIFGGCGYMYIVMVSSLSLQMRILIHRLDTIEARALSIYEANFGPKPKLNDYDLYRDRRFSKCMDYCLKRCVEHHKVILSNIAELNDFSSMPLFAIFFVAALIIALAMLGSSFWKVLPGNTIASLVLCGTEIAFIGTLSSLGQSITDLNEDIRTAIYGIKWYHCNKRFCMNVRILLVKTLEPVTFTACGLIKICYDTYANIINSAYSYYNIVSATGS

>HhalOR9

MNIIDKFTDYFESIDTSDDETNRFMEEKYKGWLQITLIYTKLDRKNIHKTCIQYFFYVLVVFYHWIFVVVGLIDTMGINFILYSKGAYYLGFIQMACCGIITTTVYRDKLVIFHRSMSKNFYDYKEKDTEFIERFKKECDRDVKLFSIVPVSTVIGAVIGFVLSPVLDKYGTHTPEEISSLNINLPAPIIAYQFSTHNNMGFFFAYFLQVCTGCVVIFIIAGGGYLYIIMVQNVCCQLKILIYSVEKLEERADECYYQMSGLKMKHTEKEKFSDQLYDYCYNQCLKKNFEHHQIILRLFDCVRHFFSVPVFMAYLIASVVIALSLLSNNIRELPGTSITSSVLGCIEVSYVAFISFLGQEIIDLNDELRVKMYETRWYYASNQVKNSIKIFQQMTLKPLVIMSASIVPINLDTFAMVMNSAYSYYNLINAFSD

>HhalOR10

MWFLDGSKEAEDSGPVEAFIDKQYYRIMQVSALYLRMDPKNRVLSFLQLITYVVILAYHWSLLVNGTLVLFDINMVLFSQSVHFSLLVQLAMILPICFQIKHQKMYQFHKMLSYEFFDYEDPPIIGEDHLRSAMAQENRKMFMIPVGVVAAAGAVLIMAPIVDMNAGSFDFNKTANIFIDNLPYRYAPYPFPVEDGFGYYFALLGQMLCGLLLTLIIAGGGFLFLNLSQNVVLQLKILHSSLDNIEGRTEHLIIKLFGNFDKKKDSYGDSRYAYCYSICLRKNFEHHQIVVRAFKILEELASVPVFLAYTTGTIVIALSLISAGSMKELPGTTLASMILCGVEVGYMFLFSVFGQRLSDLSSDLRFKLYSIKWYLCNNKVKSNMLIFQEMTLKPLTMTVGGVVPANMETFSTVMNSAYSYYNLVNAFDGS

>HhalOR11

MWQYHEDSEANRVVDTYIFEHYYYLPQISGLYPKMDGRNRIFTTLQLIISVLTLLHQMAILIKSTIALYGFDMVLLCQNVHFCLLIQIALITVGNCLWKHFSLYDIHRLLSYDFYDYQEPPGIGEELLRSTMETERRRLNCIPIGVAIAAGVVLALSPIIDFNAGSFDFNRTASVFSHHLPMPFTQYPYHVKDGFGFGFALFGQLGTGFLLTAIIGGAGFLFINLTQNISLQLKILNNSLENIDSRIEHLYSKLFGKMDRNIMKSLRHDSRYAYCYTKCLRKNFEHHQVILKSFHLLEDICSLPMGISYLTGTIVIALSLISTGSAKELPGTTMAAMILCVVEVSYMFLFSAVGQRFTDLSNELRCKMYNTRWYMCSKEIKSSLMIFEEMTLKPMTMTGARIVPANMETFTTVMNAAYSYYNLVVAFDIK

>HhalOR12

QGIEASPSELPTMFGQEKVRSILKNYFTTEGKREDSLAFEAVRRFIHGMVFYKTYVIPLPFFIVLNSIMVVLLLTENIIQIAHINWFGKLVEYNLSYPAVFYSLLVIKNRKDLIYISESIDVWWNYPFLEDKTKMIKKKAEAWLKRFTSAYYTLMILGFLCIFFQPLGKFIWNGRTDLKDLLLFKCWSPVPLDKTWGLVTIMVFEIIAQFSPFFMYSAIGSYMITMTILFYHQTNLIGVAFTTIEKRVWMMVRARTETSNGAYQIYSKNLQQEIANCARNYQNLYEYLKKMCEIFNVTTNILYYTGMCLLVSSGLQIVTKKTNALLLFQAVMLITSVIVNQYLNSFITETLSEAQSSLRISVYNCDWYNMPKKCQININLMLILVSYSPKLTTILGARCDKEFLSKVINASYCYFNALHSMRADRTGSLE

>HhalOR13

LRVHQFSGVMRHLAAITDSDVIDGLNIGFLKIFGVWGIINDYRTTGKKNFVSKFQVFITLLLAVPYIFCQNLSYFMIKVDIQKATFLNLHALPALQMCCKVMVFWFRLDSQSRLYSLVKKDFIHVPEEKKDEAAKIFKKITTKANLLCLAAFLVDLSITAFSIADPSISVDYLLHGTGSMTAVVGGKQKIMGGWYPLPMADWPYYEAIFIFETVLMIWSGVLLALYVSLFYQVLICLYAQFVVLNLHVSTLKNHFSIDCDRKNINNREVHSSLNKEEIYAVAKDHQRLLSYANELRSVYNPLVTMILGMGVFVLIIGVFQFLFGKSTKPMFIFKFMLLLAYQGVEVSMFCFGSSALETASSDLQFAIYSSDWYKKDIQFRKAVQMVMVGARKGVTLTAVRMYPVNVETIMAMLQFTYSVATLMSRMTE

>HhalOR14

MTVKKDELDEEFFYDFRETRKWYKIFAGSSMSKQMKPTIFLEIYRIFAFLFLIMMTFMFLFTALLTTEYLAEFSEAFHYFCILLTMAIYIPVERLAKNDIDKSFLKLKKCFYLYNGELNNTQKKIKRQTIEYIKFTDRVFFWMLIFVCVLYSILTPLKDYFYPHLRLERSEIIDRKLPIPFYIPFEDSYGITFIIVFLMECCCNMMVHSLITSIHEAYISLTGQIYGEMKLINYSLSHIEERAIKLYLSKQQKRVHRLKCIYRLPAFQKCFKKCLKEDMIHHQTLLSSIKLVRPFTGRIIISVFTLCSIVWAVDVYVISKMIEQASAMDKVLEFLLMLFTEAVYVFDMCHFNEKIRSENDELFMQIYSTPWYNYDRKLGQNVHLMLMNTIKPTKLNSNLFNVSASFETLMPIISATFSYFNVLRKLKN

>HhalOR15

MGKMFDVTEDIEIDKFIDKQYFYLMQISCIYIKLDKRYRVFSIIQWIFSFGNLVYHYLLLARSTMLMMTFNMVLFSQSSHFALLLQLTMILTFYFQTKHRELMLFHKMLSHGFFDYEEPPPPGDKELRSAMLRERRILIMIPTAVALAGGVILVGSPIIDRRLGSFDLEVLDREFSTHLPYPYGKYPYQTKEGFSYYAALAGQIFNGSLLTVFIGTAGFLFLTLAQNVSLQLQLLQNSLQHVEVRAEEMYFRLFGKTKNDGTSLYEDPQFSYCYSTCLRKNFKHHQIILRAFEYTEGIMSAPMFLAYMTGTIVIALSLISTGSVQELPGTTISAMILCGVEVGYIFLLSVFGQRIGDLNMELRFKIYSIKWYLCNRALKSNLLIFQEKTMKPLTIMAGGIVPANMETFATVMNSAYSYYNLANAFGS

>HhalOR16

MQLSEKDYLLKGYQGSQCFLLRIGAMYVLQDENEVYNWIFVGIFHMHYYVFLFWILPYDVSHTIPMGHMTATLQALHYYCTFLGYSIVNYLYHFNRKSVNFCLTLIGKSFFEYEEGGTEIEIKAAQESDKNQKEYIKKLTRYVMVIVFATAFAVLMLPPFAELLTRDGPIPDDEIMNPYLPIPLVLPWDSFSIGGYLLIYVFLFLVSYNICTELLALSMGYTSFLIIYINQFKILNNSVLKIEERATKRYKKVAVKQLKGMEKFDDPIFQECMILCLKQNIKHHQILLKWMDEACHFLGWGVLCTIFTTSFLLAASGFLITMESDSSLLKSLILVQVQILELLHAYLFCWWGENLATESAKLYHSLYKTPWFYCGKRFNKLMQIMMNRASKPVIPRDPLFKINASLEVYMSILSTGYSYFNLLRSMN

>HhalOR17

MKLLISDYDVEIDGVIEKTYSGLNTISAIYPVMDMERPRLCLINLFLFLFHSVILTVMTSSLVQASLELFYINFVPFVHELHLAFLGLLSIAILWHSWLKRSNVTRLHRIITQGFFDYKEDLEDKMKVLGEDRIKERKRHILYVILIGFAASVVVILIPAVNQFGTFRYNSTAYKVNFDLPVPLPYPFMGTEPLDLLPGYTMIVVTASSIALMNCTKSFMAIDCNLHIQMQLKLLLHQIEIIKSRAGRLYIKLYGTEPKYNGLKLYDKKFMKCYRICLRRSIQHHQIIVRALNEFNEVFGFYVFFYYLTGTLDIAMSMLATSSTKEFPGTTIGALIICIVEVGFVFVFAHMGQNITDLSVELREVIYDMPWYRCDQETKTTMRVLQIATLKPLSFSFYSLLHINYDSFATVLHSAYSYYNLVNAKN

>HhalOR18

MDTIKLSAEDIPIDRQITHDYETDRKNLMYMSGFKVNNDSNLKYILSKIYVYAIIFFYSFLVSMGSVAAILIHDKKLKLEAVHWTFLNLVGLIFMVIRYTYVLDEPLKTIRTGFFTYENEFIDDRYLERKKYHVRRIRIVSNMFLGLAISNGILLSFIKQVKKSSEVGNYGKSINIGLPIPIFLPFDTTTTNGFVLGYLINVVAFFYLVVITSSAQQIFLSFMEQGIVQFEILNISISNIEKRAYYIYSKGKACAEGISIELYRTRKFQNCISQCLRQNVRHHQTLLRFRNDIKTYVEAVFVLLILASIVVFAAIMFVILELQDLSEASTLFWLLSTEIIYVFVVCIYGELFSQESAKMFYALWDIPWWNFDKRNKVILHIMLSNSMNPVVINAPFFSSNMSLESFGDIMARAYQFMSVVRNVDRK

>HhalOR19

MLKDIKAILKEKIEQAVGKALMENQGIYMVISGVYPVNIYYSLTAILCQLFTLSFFMYTCIYFYYNEYNLSMFSEAAHFVVVVFCSTSFYIIFWFRKDKVHNITKLLEQEIFKYKGTLDLETRSEIDEMTLKMKNRKNKISNIMTIVYIICGLFGVILLPIIFKKSAITVKEERILLTVPVATYYPVRGDFGWYLCSAQQYLTTFFIISALNGSDVAFSCYCEEGCNQLRILCHAIRHSLRRARYLHTHIFGKEPLTLRDETFAICLKMCLDESVIHHYRLIRYFKEIQSLFSWCVMIVMMAGASMLCLSGIYFFFDVVGFGTKISFAFYLSGELMHTFIYAWYGQQISEMSAEIREVLYEIDWEDCSKTVKPYILIMQAYTNNPIKLKGGDFMEYNLNTFGNVCSTAYSYFNLMSAAVPSSS

>HhalOR20

RLRAARAMKYQAISDKDIIDGLNVKYLKMFGLWKVINDYRATGKRNAILKFEVGLITLLVIIYSICQFMSFFNIQFDVQKFTFLNLYTLPGFQAVFKILVFWFRIEDQCKMYNLVRKDFLKIPSHKEAHVKRIYENISAKSNMFCNAAILINTSTVILWILYPGLPVDYILYNIGSTDVVRTGRNKILGGWYPMPMGETPYYEIIFVFEAVMIIIGGLNLAVYICQFFQVLMCLYAQFAVLGYHLSTLKFNAVDGDDRIEDSENNAMYKELNEILEDHQKLLSYANELKSVYNPLVTVILGMGLLVLIFSVFQFLFGSLGNMMFLFTSLQTLTFQAVEVGMFCFGSQFLETASSELQLAAYSSDWYKADIKFKRAVQMMMVRAKKGETLSAVRMYPVNVETLMAMIQFTYSMITLLSRMTE

>HhalOR21

MGYEAVIKDTDVIEGLNIRFLKLFRMWNAVNDYRSTGKRNIIIKIYIFGSFLLTVPAAIFQIESVFTIEFDIQKATFMYMHTLPAISLCCRIIVFWFRMDSQCKLYDLMRNDFFDIPERLRGKVKEVYKETNKLSNICCLIPFIWNTGIEIWFIAFPGVSVEYIQHRTGSMSAVTTGSKKILSGWYPFPVEEYPNYQLVFAYETLCLLWAANVMAMYFSLFYQILMCLYAQFVVLGFRLSNLKVEFRDDDIATQKTSGQEKKNPRINEELNAILRDHQKLLRYTEELRSIYNPLVTMTLGLGILIITIGAAQLLLGKTSDPSFLFQLFQIFSFQFIEMSLFCYGSSLIETASTDLHFAVYCSDWYRADTKFRKSAQMMMIRARKSMNLTAIVMYPINMETLGSICQFTYSTAALMSGMVE

>HhalOR22

MVVTLWSFLKSYFLTQEKPEKAGYVFNTAHYFLYGLTVYKKKTRLAIPMEFILLLSSVVMFSSILQSLLDFWKTKWIELLCIYITLVTPILCSLTVILHRERIRAVSEQVDRWWNYPLLDKETKKMKTLAAVTMDRFNRYYFSAMYVAAAICGVLPFQQNNLSAEKMKPMDKLIFPMWTPFSIDGSWGFFAASLLQVLAIWFAYFSFGRLFSYFMSLSFTISNQMKLIGRACQTRLRRVEIMMKDLDITDNRSLDQLYIEMMMKDINCCIIHYQILYGHLKEIRRAFSLITNLTYHAGMWAMCLFGVSFVKEKENPYTLIRLASAIVSLFFHQYLISYVSEVLTDELENLRLAVYSSQWYNVPQKLKKNIHLFLTLLLYIPTLRTIMGTKTNMENLSQILNTSYCYFNIILTLKSRFSVE

>HhalOR23

MELLAWPRSLLSKYVAAVIKDEPRGYVYTSSVTIRFGIAIMEIRKNEYLVVRYPLLMLFSITTILWILIDHILAFSDARWFSKILDYNYSGGSLLFTYTLKVKRAEISVFTQVLDQWWSYSFLDQKQEELKERLQRKLKKFNNFYNRFMVTMGIGFGMMPLGRYFYETEKSSTNLLLERCWSPFPLDTWWGFSITYWVELTSMLSLFYSWAYIVCFLISVMETIGKQLQLIGISLVTVEERVKKAMSLFKVKDKEEWYELYVRMMQKELKNSVKHYQKMYRLSEEACSVFSTPINVCFHSGIIALVASCVKLATERENKFVIFQAIVMVVNVLVNQFIVSCINQILTDQLDSLRDTIYESSWYKLPIPCRKTIHMTQVMLLRPPSIRTVMGTKTDMEFFATVVNTSYFYLGALLSMNVKL

>HhalOR24

RTMGDKAAITDSDVIDGLSIRYLKFFGLWGVINDYRTTEKRNSVLKFQILITLLLMLPSIICQYVGFFVIEMDIQKATLLNFHSLPPLQLCCKHLLFCFRLDSICRLNNLMMKDFLKESIPEYQREKVISIYRKISKQTNIFCLVAFVVITIGVSLICIPEVPVDYILYHTGNMFAITTGRCKISTGWYPLPMDKSPWYEIVLVYECIVVLWASSFVTAFMFLYFQILMSLYAQFVVLGSHISDLNIDSDYQKNSIEYRILNLQNNRELHRILQDHQKLLSYAAELRSVYNPLVTMILGVGMSVLVIAVFQFLFGQTGDLMFIARAFFYVLYQSVEVSMFCFGSLFVQTASSDLQFAIYSSDWYKADVKFRKAAQMMMIRAKKGETLTAIRMYPVNAETIMAILQFTYTVATLMSRLVE

>HhalOR25

LSGAMEAMRDSDVIDGLSIRYLKIFGIWKIINDYRTTGNKNMILKIQVIITVMVTLPSILSQFLALLVIEVDIQKATILNFHALPPLQALSKQLVFWFNIDSICRLYNLMRKDFLEKTVNDIEREKVEFIYRKFSRETNTTCLVAFIVINIAGSFVLLFPGISVDYILYHTGKLSAVTTGRRKISTGWYPLPMDTSPCYEIVVLYEGILVMWCGYAILVFMCLYYQLLKCLHAQFALIVSHVSTLKIDYEYEKNSIEYRFTNQQVYSRMYRILQDHQKLLSYAAELRSVYNPLVTMILGVGMSVLVIAVFQFLFGQTGDLLFMVRAFFYVLYQCIEVSMFCLGSLFVQTASSDLHSAIYSSDWYKADVKFRKAVQMMMIRAKKGETLTAIRMYPVNAETIMAILQFTYTVATLMSRLVE

>HhalOR26

MGNEDAIKDSDVINGLSIRFLKLFGMWNAINEYRSNGKRTAVINVHTFGTLFITVPYVVCQLQSFFMIEFDIQKLTFLYLHPLPASSLCYRILVFWFRMGTLCRLYNLMRNDFFNIPEHVKTGVKELYKKTSKMSNTCCMYVFIWNAGIEILYLFFPGMSVEYIQHHTGSMAEVKTGKKKIFGGWYPVPMSEYPYYEIIFVYESICLLWAATLLAVYFCLFFQLLMCLYGQFVVLGYRFSNLKVDDADRKIRKNNPYNRNNSSRIYEELHQILLEHQKLLRYTDELRSVYNPLVTISLGIGVIVLIIGAVQILMGKTSDPGTIFQIIQVFSLEIMEVSLLCFGSSLIGTASSDLQFSIYCSDWYKADVKFRKAAQMLMVRSRKSSTLTAIVMYPVNLETLGAILQFTYSSTAVTVGMIE

>HhalOR27

MTYGKSIKGLFFAEEGNKEMGFISNIARFVLAGVTMWQSHSRFVIPSALVGFFGVLMLLSNLIHTIYNFDKLNWINKVVDYGWTSVYLTSSYIVVYHKEEIKSLSEELDRWWQYTYLERETAKLKKKSEDWMRTFNSYYVTCGCISWVFYVGLPAVKFFYKTPEQRNEALIFVAWTPFQLDTTWGYFITYGLEVVMMAFAQCIYIQIFQLLMTFAIVMGHQMRLIGTSLLTIFKRIKRMMKNMTFESRIEFYEVRDLLLEKELGNAIMHFQNLYRCSSRLSRIFASITNVTYHGGVWVMCSIAVKAATEMSFVAVLQAFMMIGAVTLSQYVYSFFNECLIEEVQKLRNEVYDSPWYEMPTKSRKTFQIFQSMLDSDRFPTLRTIMGIRTNMENLSKVINASYCYFSMLITIKSSSETTE

>HhalOR28

CLGGTKRLERFVTKRSYSSMGKIWLKNIEEGLKGPRMFLSLAGLGVVFKGRFAFASFVFANNVLALCLLAYSIYYYLDQIEKVSNIVHHAVLIMDICVALVLGYFRHDEFIAILSDPSSSYHYESQMMSKKIEEVKGIACKNYTGMFKYIVLSMVYLHVLMNVLAICQRYFNDEEILLLFPCYFPFPIDNYPVHIAVIIWQELVVSNIGILVFSGLLFIHCIYTHVKSEIDILKFAMVNIEERAYEMAMNQKESRDPTSHPETLSRCQVKCTRMCAEHHSIIISYFHNGGFLIETVYFLVILTGLVVCSCTGFALISENTSLKIKFVGIMIIQIIFLYIMCWLAEETAEQSQSVGDVVYGMEWYRLPKECQTIFLIMMIRAGKPLLMRMLTGQKVDLAAYMALIKASYSYFNMMLATMQ

>HhalOR29

MIDGGIEDSDILEGLNVRYLKIFGLWKVINDYRKTGKRNGIIKFEVFCTFILTLPYVICQYMSYFNIEVDIQKATFLNLYPLPALQMCCRIAVFWFRIDIQCRLYDLIKKNFLNIPEGSRAQIEEIYKRISKVSNMCCMATLIVNASIVSLYVVNPGISVDYILYHTGSMTEVTTGRKKILGGWYPVPMAESPYYEIIFVYEAICILWGGLFLAVYFSLYYHVLVCLYAHFTVLGFQIENLKMKSVKSGGSKSCINRDQNNNSIVYENLIDMIRDHQKLLRYFEELRTVYNPLVTLTLGIGVIVLIIGAIQFLLGKSINPGFIFQISEVFALQGLEVCMFCFGSSSIEAASSGLQFAIYSSDWYKADVKFRKAAQMLMVRANRGVSLTAIRMYPVNVETLMAILQFTYSTATLMMRMTE

>HhalOR30

MGYEIKDSDVIDGLSIRFLKFFGMWKAINDYRTRGKKNIIIKIHLLGSLMITIPYVVFQVQSFFTIQFDIQKITFISLHALPALQMCCRLLLFWFQLNRVCRLYDMIRNNFINIPEDMKDQMRDLYINTNRTLNLNCLVVFIWNIGIVLIFSISPRVPIDYIRYHTGSIAEVKTGRMKILSGWFPFPMDEYPYYEIIYVYEVFCLSWATTLVSVYFCLFYQVLMCLYVQFTVLGASLSNLKIDCPDFRQSKRHKNIIQNRNSNLYEELYECLQDHQKLLRYTDDLRKVYNPLVTVTFGIGILVLFMGALQILLGKTCDLSFLFQLIQMLSSQFLEVSLFCFGSSRIETVSTDLQFAIYSSDWYKADVKFRKAAQMLMVRTRKSSTLTAIVMYPVNLETLGAIAQFTYSAAALLSGMVN

>HhalOR31

WLPIMSLQFETTHSSMIHLLRRSGLKLPWVKHRSYWNQFCYFAYDILLFTIGLYQFICSACSIIFVPTFQDMCTLGIVTSVLATGGSITLFYFFYQNRLEKFTENWNALNDNILNSDLDKKDFFRQMFLQVAKNNESFTKTILFVVFWTPIIYCTPVPVVDAIKQSYRTNLPLPILYLYDDRQPVVYELTFFLHMMGLVISVMKKFGNDCFFLALFKIHIAYLRYLSVAIKCEGTKFSKCNNKIIKQNLISWIKIHQQIVKNAQDLIRLYTPIIIVYHVNLICIVVFGLFTQIKNDRDSSVQRIGTAMFCTVNIFQLYMQCSSAEELTNEAEKVSQEIYNTPWNEVDECNADIIRLVLKMASRPVEVTAFKAPTFLLNKQSFVAFVVNTIRAFMTFSKMSDLRTSISDNNLNLFTESI

>HhalOR32

MAPERVMEKDLMDGLSIFFVKYLGFWGTVNTYRTSGKVNLLFKIQWFLTFLCVPFPIFQFMSPAYIKFDLEKATIIILNTTSFFQMTFKQAVYSMNIKEHAILLEVMTKDILRSLPEYKKPHAKRIFEKISKRCNFWCFIAVVITFTAVSLWTMNPCISSEYIANHVGNMKDVTTGPKKILGGWYPVPFTRSPWEEIVYAYEFLWFFWIGYNVAIYELVITMEVLTLHAQMSVLNYHVSTLSKKEIVQHSGKKGLTQREVEDLFYQELLAIIRDHEMLLGYGNRLRNCFNAYITMLLATGGLLLIASIFQFLFGAKDAVVSVNYMLYLLYEVAEFIFLCTATTMLETSSTNIAFSIYSSEWYTSDKRSRDTIQMIMIRSRKPMSLIAVKMYPVNVETLMSVFQFAYSASALISRMVE

>HhalOR33

MSEPIRESDIFDGQTTFYMKYVGIWKIVNTYRTSGKMSLVFRLEWYLTLLLSVPFQVLQVISPSYIDVDLEKATILILNTVSFLHMVAKHGTFWWNIKGHAELFRLMTKDLLTSIPQYKAAEAKKIYQDATKRCNFYCRMIVTITYSVWSLWTFNPTVKSDYIQFHTGNMKDVTTGPKKILGGWYPLPFSQSPWTEIIYFYEAILLLWCAVIVSIFDTVVTQEVMGLYAHLSVLNFHISTLKKEEIIFHSKREIHTEQEAEDLMHKEFVAIIRDHQYLLRCGKIIKECYNTYITALLLAGGNLMIITVFQFFYGKKDIPTTINFVFYLSYGVMEISLLCWTTTLLETASTNIAFSIYSSDWYTCHKKLRNTGQMMMLRSEKPLSLAGFKVYHVNLETLMNIMQFTYSSSALMSRMVE

>HhalOR34

MALPTVDKKPIDEYYIGKCMKNNYGLEFWLKAGGVFSIGKNGRFIYLISGLYVIAFFMVFPLLVITAYKAAEGKELDIVCEAIHFAIFTFLEMIFLITCIVKNRVLAKIFTIMGKGFYDYENTLDEECLSVAAKTLNESKRRKKIIAKVFIVVVMCACLSITILRPIMKFLLGEHLLGKPDDGILRLIPVNMWTPFNKDSWYAMVIFYLSQDVIGYVTPGLVFGCTLFVVFACEDVGSQLIILGQSLKSVIRRAERLDMPTDEALKLCFSHSIRHHQTILMCVKTLEKLLYIPGLGLLFGSTILMCICGFIFVSKEVPFPSKFVFGMFLLSELMLIFLVCWCGENIQKTSTLIFDMVYSSEWPDNMSSMKNYVLIIQLRTIEPIKISFGGLMDASFETYSNICSSAFSYFNILIAVN

>HhalOR35

MIAVNEAKGSDKPKEWKPVSRSDLLEGLSVIHLEVFSLWTALNTYRKSSKKSYRLLAYIIFTVVVVSPYLISQILCAFYIKMDLQMAIYLILNTMPPYQAFTKMGVFWFRMEEMATLFDLLREDFLTCIPLHKKSKAKEIYRSITKRSNLFCFLAFFANTMTVVTWIAMPGFDTNLEGTGRKKIIAGWYPFPYSETPYYEVVVTYESVLMVWFGLSLCPYECFLVQLLSGLCAHFTVLNHHLATLTKEDVFGKIPENHGGVNAVMNEELKKIFDDYNKLLRYGDILKDVYNVFVTIILGMVMTDLITASLHLLFTPSDALFTVNLILFFLHSLVEIALICFTSSYVERVSFQIRFSAYSSDWYTADKKYRFTAQMMMLRAQRPLTLVAVKMYPVNLETLIAILQFIYTTCAVLSKMK

>HhalOR36

GYQVIEDSDIIDGLSVRYLKIFGIWKVINDYRTSEKRNYILKFQLIITLVLAIPSIIPQYFSFLVIQVDIQKATILNFHSLPSLQVLCKLLVFWFRIDSLCKLYNLMTKDFLDKTIPDCEIESVKHIYTKMSKRTNIIVLTACIVINCGVFLLVLFPSISVDYILYHTGNMYEVTTGRKKISTGWYPLPMDKSPWYEIILVYEGLVVLWAGSFIFVFMCLYYQLLMCLHAQFIVLGSHVSTLKIESLFEELEATDRIQNVEMYKKLYRILQDHQKLISFADELRTVYNPLVTMILGMGISVLIIAVFQFLLGKTGDPMFILRSFMFLLYQCIEVSMFCYSSSFIETASSDLHFAIYSSDWYKAGTKFRKAAQMMMIRTRKGVTLTAIRMYPINLETIMAILQFTYTVATLISRFTE

>HhalOR37

MIKEGIKDSDIIDGLSLRYLKFFGIWRIVNDYRTTGKKNAILKLHLLGTLLLGIPYVIFQFMSYFVIKVDIQKATILNFNAIPALQLCCRMFVFTFCMESQCRLYNVLRKDFLNIPKQNLEVKEIFMSISKTSNFCCTMSLAVNGSIVLFYIIYPGVSVDYILYHTGSMAAVRTGRKKILGGWYPVPIDQSPYYEIVFAYEAIVLLWGGFFLAVYFVLFYQVLMCLYAQFSALGLQMSSLKIEQYRSDINTSLNHNYISSTVYEELYKLLKDHQKLLRYTEELRNVYNPLVTMTLGMGILILIIGAFQFLFAQTGDPVYIFRFLQLLAYQGIEVSLFCIGSSSLQTASSDLQFAIYSSDWYKADTQFRKTAQMLMVRANKGVTLTAIRMYPVNVETLMAILQFTYSVSTLMSRMTE

>HhalOR38

MDYEAVIKDSDVIDGLSIRFLKFFGMWKAINDYRTRGKKNIIIKIHLLGSLMITIPYVVFQVQSFFTIQFDIQKITFISLHALPALQMCCRLLLFWFQLNRVCRLYDMIRNNFINIPEDMKDQIRDLYIRTNRAFNVTCFVVFICNGGIQLIYIMFPKVPIEYLQHHIGSVSAVKTGKKKILSGWYPCPMDEYPYYELIYAYETFGLSWSSILVSLYFCVFYQVLMCLYAQFAVFGARLSNLKIDFGQSKKHKNIIQNNLYEELYECLQDHQKLLRYTDELRKLYNPLVTITLGIGILGLSMGAVQIILGKRCEISFLFQLIQMLSSQFLEVSLFCFGSSCIETMSTDLQFAIYSSDWYKADVKFRKAAQMLMVRSRKSSTLTAIVMYPVNLETLGAIVQFTYSTAALMSGMATN

>HhalOR39

MSHEPFIKDSDVIDGLNIKYLKFFGLWKVINDYRTTGNRNLFIKFKVFLTLFLAVPYVLCQYICYFFIKADLQKATILNLHSLPALHICWKILIFWLRMDSQCRLLGLVRKDFINVPKEKKKAAKEIYEKITKKANLFCVAAFILDSSVIIIAILFPGVSVDYILYHTGNVFDVTTGRKKILAGWYPLPINESPYYEMVLVYEAVLVGWGGMMLAVYDSLVCQSLMSLYAQYKVLGYHVSTLKIDSHSRRTKNGENNDSEMLKELKVILQDHQRLLSYANEMRSVYNPLVTIGLGIGIGVLIIAAVQYLFGKTGDPMFVFKSLQFLASAGLEVSIFCFGSSYLETASSDLHFAIYSSDWYKAGTKFRKAAQMMMIRSKKGVTLTAVRMYPVNLETIMAILQFTYSVATVMSGVTE

>HhalOR40

MSYLPPITESDVIDGLYLRYLKFFGLWGVINDYRTTGKRNRIIKFQLLITLMFAVPYIFTQYLSFFVIKVDIQKATFLNLHTLPAIQICCKIMVIWFRLDSQCRLFNLVRKDFIYIPEYNREAANKIFKEISNKANILCIAAFIVNTAVIISSIAVPGISVDYILYHTGNMDAITSGRKKILGGWYPLQMDESPHYEIVFVYETTFILWAGILLAVYISLFYQVLMCLYAQFSVLCIHVSSLRSDPDAEKKYRNRKVDSEIYKELYIIIRNHQKLLSYANELRSVYNPLVTMILGIGIFVLIIAVFQFLFGSPGNPTFIFKSLLFLAYQGIEVCMFCFGSSYVETASSDLHFAIYSSDWYKADIKFQKAAQMMMIRTRKGVTLTAIRMYNINVETMMSIFQFTYTVSAFMSRMNE

>HhalOR41

LLQHLALDRSTTSMEPPRYVDQYGELFKWQRRCGFSTPWLEKPYFYFRFLDLAYDTITISMVLYILLDYSYTMLTTSLSFQDICLFGIGFGSSACSMFITTYQILYSYRLKEITDKMDSIGKKIMENDLGGKDFFKQEYVKNAKFLSIFTRCSMTSIFTTPFSYFLSVPVVEWFEGNYREHLPLPLANVFDDRQPVVYEIVVIVLSAGISIATAKKAALDSLFISFLSIQTTFLKYLSVAKDEMSKDMRFADDGRSRRKLLTWVKLHQEVIKNIEELVEYFSPIVVVYYIVVIEIVVCGAFVELKKDNDSIVQSISVGSYVMLTVIFYYLLSNKADELTTEVQKMVAAEYNLPWYAMKKSEVSIIKVVLMMCNKPIHITAYQAPVLRLNRETFSQFIVRAISALVTFFQMKDIFG

>HhalOR42

MDPTHFLPKLSTSMEPPRFVPHFRALLRWLTWCGMATPWIKKSFSIWRLLNLLYDLMLMALVTYIVFCYVYTIMTISVGFQDLCGLGVSASNYMCGFFATIHQCLLKDRLKVLTDKLDKIVLDIIRSGLGEEQAFLELYNNNSKTMAVLVNNSVILAAIGTLIYCLSVPAMDWYADQYRSHFPVLIESPFDERVPVAYEIVVFLVAACMIVSIAKKIVTDCLLISLFKIEIAFFKYLSLSLASMKKGFLKGDNAFIDRKLKLWIGIHQSVLRSVDELILISSPMVIMYYVTVISIVVCGTFVQIMKDNENIFQSLSITVFISITLLYYFLLANTADQLTAVAQNVAHAAFDVPWYQMEKKHSTMVRMVIAMANRPIRLTAYRAPIFVLNRENYAGFVVSAISAFVTFCQMKALYG

>HhalOR43

MIYQDAIKDEDIIDGLDVRYLKFFGLWKIINDYRTSGKRNVVVKLHLLLTILVSVPYVFLQYMSYFFIDVDMQKATYLNVNTLPAIQACCKVLVLWTRLGSICKLSGLMKKGFIEISEENKAAASEICKKITYKSNILYKTALCMNAGSAVVYLLSPGISVDYILYHTGNMAAVTTGRKKHLAAWYPLPFDNSPYFEIVFAFEALLISWDAIILVVYICMYYQILTCLYVQFTLLGLQMSSLKNENIKKKDNIIKQANRTTYDKLYRALESHKELLSYTNELKTVYNPLVTMILGIGIFVLIMSVFQFLFSKTGDIMVIIKSLQFLGFHGLEVSMFCFGSSAIESASSELDFAIYSSYWYEGDNRFKMAVQMVTMRAKKGMTLTAIRMLPINLETMVSIFKFTYSTAAFMSSVTE

>HhalOR44

MEPPRFVDYYSGLIKCLMYWGMPMPWWVQKPSKTVWLLLVTYDTLTLAVTCFALYLYVYTVTLDDVEFEEVNVLPPAICFKLCVLGISLLQFLDRGRIKQLSDDLDAVVRTIIETDLDQDESVKESFIQMYSKKSKFLVNYCRTMPFFAVSYYVIYFGSVPLIDWSEGIYRSHYPLPLLTPFDGRKPGIYEFLVFVVLISLTMVSGKQINNTCIFLAFFNVLRSFLHYLSLTMSEMQKISYKERNDPSIRRKIRVWIQIHQEVNRNLQVLLKIFSPVVIIYSIYLLLYLITAIFMQMQKKEENIYQTAAEGLAVIGMVRQIYVIFNTADQITTEAQKLANAAYELPWYQMDNSMRSTINMIMMMCNRPVHITGYRAKSFIVNRETIAGLMTSAVSGYLTLCQMTDAFGPKESSRQ

>HhalOR45

MDNQGAILDSDIIDGLDMKYLKFFGLWKIINDYRTTRKKNAILKFKVITTLLLTIPYIVSQYLSYWMIEVDIQKATFLNLHSLPALQICCKVLVLWFRIDSQSRLFDLVKKDFFGIPKSKEDEAKSIFSKMTSECNKLCSAAFLINTSVVILSIIDPGISVDYIMYHTGNMHAVTSGKKKILGGWYPLPIDKSPYYEAVFVYEILLIIWGGILLAVYVCLFYQVLMCLYAQFSVLALQVSTLKYSYIQDGKGRKSVNSKLYKELYEVIKEHQKILRYAEELRSVYNPLVTMILGVGIFVLIIAVFQFLFGSTGNPMFIFRSLQFLAYQGIEVSMFCFGSSYIQNASSDLHFAIYSSDWYKADVKFRKAAQMMMIRAKKGETLTAIGMYPVNRETLMTILQFTYTTSTVLSRITE

>HhalOR46

MQYQQPLRGPDVIDGLSIWYLKLFGFWKIINDFRTTGKRNLFFKFEFIMSILISFPYIACQFSSYLTIDVDIQKATLINFYCLPAVTMCSRILVFWFHADSQCRLFNLIKKDFLCIPENKKAETRKIYRRVSKSCNMMCMFAFVLDLSVVFTTVGIPGIPVDYILYHTGSMFDVTTGRKKILCAWYPLPMAEYPYYEIIFVYEMMCVLLGGIYLPIYASLFYQVAVALHAQFLVLGYHVSTLKINPNIKQKKKNMSSGITEDLYKILLDHQKLLSYADELRSVYNPLVTINLGGAIGILIVSVFQSHMGETRDIVFVLKSILYAASIMIELLMFCYSSSLIQAASSDLHFAIYSSDWYKADTKFRNTAQMMMVRAKKGVNLTAIRMYPVNLETLMSIFQFAYSTSALMSGMLEE

>HhalOR47

QQGIADSDVLDGLSIRYLKFFGLWSVINEYRTTGKRNGIIKLKLFITLLLSIPYIFSQYLSYFIIEVDLQKATFLNLHSLPALQICSKVLVFWFRIDNVCKLYNLIRKDFLSLPEHKRDGAKCIYMKITKTSNMLCKAAFIVNSSIVALYVMQPGISVDYILHHTGNMAAVKGGRQKIMHGWYPLPIDRSPYYEAIFVYETMLIIWDGILLAVYDSLFYQLLMCLYAQFTVLGFHLSTLKIVASQDPNSRLNDSNSPIYRELYKIIKDHKKLISYANELRSIYNPLVTIILGMGIFVLIIAVFQFLFGGTRSPLFIFRSLLFLVYQGIEVSMFCFGSSSIEKASSDLQFAIYSSDWYKADIKFRKTAQMMMMRARKGVTLTALRMYPVNVETIMSILQFTYSVTALMSRKAEIK

>HhalOR48

MSYPAEIRVSDVIQGLDIRFLIRSGMMRFINDYRTTGKMNPMIKIHLIGTFIISLPYMVFQCLSLFKVQYDIKKGTFVILHPMAAFQIYCRILVLWFNIERQGKLYNIIRKDFLNIPKEMSHDASELYKKQNRTSNLCCNATFLWNASIELVYIFFPGVSVDYIENREINKKVVNTGKNKIFSGWYPVPMSEYPYYEIIYIYEAMCLLWASTLLGLYFCMYFQLLMCLCTQYVALGYRVANLKIDPVIYKLDKKYKSSIYQELCQIVKDHQKLLSYTDELTSVYNPLVTMTLGIGIAVLIIGAIQFLLGKTSDPEIIFKLIQMFSFRTFFEVSMFCFGSSRIEEASSDLQDAIYSSDWYKADSNFKIGAQMMMIRAKKRVNLTALFLYPVNLATLGSIVQFTYSCSALMSGMAE

>HhalOR49

MFSWCSRKNASLSENCDEKSEKYHIKKAFEENYGFWMIFGGFYPCVGILPCIYIPSAVFLSIIFIMTTVNISNSEIAIKSESVHFIVFITIELTAMTVFIFKKKTIDEIYRAMGRRFYDYENTLDEECYDVIANAYKSGRSRKKIFHDMFVGCSMSTLLTASIVRPLLSYFKGDPDPNDGILRVLPVPLWTPFKTKTWYVNLLFFLAEDVIAYMTPGIVFGCILFVVCASEDVGAQLIILGHTLKSVVRRAEGLDMPREEALKLCFVHSIKHHQMLLKYIKSLEAIIYLPGFVLLFGSTILMCMSGFIFVSKEVAFISKVEFFLFLLSEMAVIFLICWCGEFIQTTSTQIFDMVYSSEWPDNMESMKNFVLIIQLRSIDSIKLNLGGFMVASLETFGNICSSAFSYFNLMLAVN

>HhalOR50

MSLSSVFGNMLQIFLNEPSDPGKGQVYEDSRSFRRGLAIWKSKTRIVIPSLLILFMEFIMFLSVIMDAVTRPNLDWVDKIAEFCFIINTLIFYSITYFLRDDMDRLSESVDFWWSHTFLKQTREDLKKSSIQWMETFNNYFMSLLALTCTGYAVLPLAIFAISESKSYSELNIFKMWAPFLQQHLWTILLLYAFQVACIFFQFFGYGVLTAYMMSVSVACKYQTRLVEISCLTIKDRVLALVRKEQDISERKTLFCTIFLKEIAESAKHYQHVYRNWKEMCRLLSRMADVVFYSGMCIVIMFGVRVATQQENSSVILNSFLFIVVVICNQYLFTIINGTFTNQVISIQNSVYNCPWYTLPVSCQKSINLFQIMVSYIPTLSTFMGVEASREFFGRTINATYCYMSALVSMNRK

>HhalOR51

MNQQAPITDSEAIEGLNIKYLKFFGLWKIINDYRITGKRNIMLKVQLVVGLMFTVPYVVFQYISFFFIDVDVEKMAFLTLHTLPGTQMCCEILLIYFRIDSECRLFNLIKKDFIYIPENKRDLAERILTKIGKNSNILCIGVFLVNLITLIFAVNFPVASVDYILYHTGNMDAVTTGRKKIFGGWYPLPMDKTPYYEIIYFFEAAFHLWAGMLLAAYISLFYQVLMCLYAQFSILSLRLSSLKVEKENSQDRNGDSKIYKELYMIIKEHKKLLSYSNELRSVYNPLVTMVLGIGIIILIASVFRSLFGTFGNPISMFLSVMFIAYQGIEVCMFCFGSSYVETASSDLQFAIYSSDWYKADIKFRKAAQMMMIRARKGVTLTAIRMYPINLETMMSIFQFTYTVSTLMSRMME

>HhalOR52

MSDHTPIKDSEIIDGLNIWYLKLFGLWKVINDYRTTGKKNSIINLQLTITLAYSIPYILCQLMSCYFIKMDLKKITFIFLLTLSKIQICIKVLVVWFFLRIQCRLSNLMKKDFIDLPEHKKSEAKQIFKKIALQTNLLSIAAFIINTSYYIVSIGFPDTPVDYILHHTGNTFRATTGRMKIVGGWYPIPFDKTPYFEIIFFYEASMMMWVGTFLAVYVSLFYQTLMCLYAQFAVLGIKLTTLDSGDGKEKIWKSDSERYNELLAIIKYHQKLLRYADELRLVYNPLVTMILGTGVFVLILAAFQFLFGTTTSTIFIVKSLIFLPYQAIEVCMFCFASSYLETASSDVLFAIYNSDWYKADIKFRKSAQMMMIRAKKGETLKSVSIYPINVETMMSIFQFTYTVSALMLKTTE

>HhalOR53

VEMELEEGMYRSGFNRIVSFVTCMRGPLENPGKGLWYGKLYIAYITYIEFAVIVNWVTIGTVFLKPGMTVEVRCFTGFPVVASAFCCIRRVDMFLNRQRYKKLMEDYLSLYDNSPELNKDVLHYAKIIGNIPRILFFITALPMIIMGVIPILVAIVGGVRVIPMLAVFPFDPTEYFFVFCATVLLQITGGYTCTLRALCFENMFNMFACRQLALIRQLSRELRRILKIPHVDDSGELKYQNDEGILYSPEEAKNVVIEELKQWVKNHQKSMRMAKELQDMYSISLCIQFAFTGLLLCTTAFVMANKVGGMMNLFFCGAYLIGLFIELLITCRIGDLILYESNMLERTVEGTHVYVLPSDVYKNWLRLILTRAKVPTRLSALGVFPLDMETYKSFIVLTYSFFTLLKELKHET

>HhalOR54

MEAMRDSDVIDGLSIRYLKIFGIWKIINDYRTTGNKNMILKIQVIVTVMLTVPSILSQCLALLVIEVDIQKATILNFHALPALHALARHLVFWFNIDSICRLYNLMRKDFLEKTVNDIEREKVDFIFRKFSKETNRTCFLVFMVINVAGSFILLCPGISIDYILYHTGSMSAVTTGRKKLSTGWYPLPMDTSPCYEIVVLYEGILVTWTASSIIVFMCLYYQLLMCLHAQFAMTVSHVSTLKIDYEYEKNNIESSFTNHQVYSRLYRILQDHQKLLSYAAELRSVYNPLVTMILGVGMSVLVIGVFQFLFGQTGDLVFVGRAFLYVLYQSVEVSMFCFGSLFVETASSDLQFAIYSSDWYKADIKFRKAAQMMMIRARKGVTLTAIRMYPINLETMMSIFQFTYTVSTLMS

>HhalOR55

MGCLGPIADSDIIDGLSIRYLKFFGLWKVINDYRTTGKKNSIMRFTVIISFILAVPYVLFQYLSYSSIKVDLQKATFLNLYPLPALQMICRILVFWFRMDRQCRLYNLLKKDFLHIPENKRVLVDKVYQKICKTSNICCTASMIVNFSIIGLYIFNPGISVDYILYHTGNMDAVTTGRKKILGGWYPLPMAQTPYYEIIFVYEATCVSWAGILLAVYFCLFFQVLISLYAQFTVLGVHISTLKFQSNKKDRKCDTKMFKELSQILRDHQKLLRYTDELKSVYNPLVTLTLGMGILILIIGAIQFLLGKSNSPGFIFKLLQVFIFQGVEVSMFCFGSSFIEMASSDLHFTIYSSDWYMAGTKFRKAAQMMMIRSKKGETLTAIGMYPVNRETLMTILQFTYTTSTVLSRITE

>HhalOR56

MFGPSQMNVFFEERPHNHEGLTSWKLRRAVSYFIAFVYFPTFCLIETTGILFGRNSELEEVIFAIAYISFMVQMVIKLSYFHWKIEDFRELCLQFEYFHTSRHRPDFAKGYLEEASDSLRRTAKVYNFVIYVNLILWNLNPLVVQPVRFVLNLTGFTDDKDATLIPDIFPVVYMFDETKTWYLRTLTGCLEWIVLNAGLCHAVALELFFMSLFLMLAAEVEVINKSAQSTEELGREIERDYSTLLGPRDDLDRIDITLLIEDHRIVLKKINKVSDIMNPLLGLAIGYCMVVLPTLGVVITKAIRDAKSTTEAFLNIITWIGATITELLVLFMYSWICAKLKDSEEGISEAVYSTNWYERDIKYKKTVLFIMMKSLRPKKMRMLYCGDMDRETFVVGLKGIYSFYNFMIGLA

>HhalOR57

MSYQDVIEDSDIIDGLSVRYLKLFGLWKVINDYRTTGKRNKIIKITVLVSFILIVPYITFQCISYFKIKVDIQKATFLNLNSMFALQLCCRLLVFWYRMGSQCRLVNLLKKDFLNIPTQKRKAVDEMYKKISKTSNICCTTYMISNVIIVVLLIAKPDTSVDYILYHTGNMDAITTGRKKILGGWYPFPMNESPYYELIFIYESICSIWTGLLLVVYFCLFFQVLFSLYAQFMVLGLHIGTLKIEDQDQVHKNDIQMYETFHGILKDHQNLLRYSEELISVYNPLVTMTLGMGVLVLIIGALQFLLEKASPTFIIKILQVLMFQGVEVCLFCFGSSFIETASSDLQFATYSSEWYEASKRLKTSVQMMMIAAVRGVRLTAIRMYPVNRETLMAILQFTYSTSTVMSRMTE

>HhalOR58

MFSYSHLNVFLDEHPKGDVYFWKLKRYVTYFFLFLYFPIWLVIEVSGMFLGRKGDLAQVAFDLSYVAHIVQLVIKMGYFLYYIEDIRCLCLRFERFHTSKHRPIFSRRLLGERGQFLRRLASTYYTVIYMNFLFWIITPLFIQPIIYGLTQAGFIAPAGPQNIIPKFFPVRYPFDETSTRNRIIIACMEFTVLSAGFTYFIPIDQFFVSVIVMVCSEIDVICKSVMTSKELTKELDRDYSLLLGSEDTRDRIDLKLFIEDHQRALRTTKKIGEVMNPILGLVVGNCMVLLCTLALVITTKMKTASSFSEVFREVFGFIVVMTTSLITLYLYSWMCGELKSSEEAVFGAVYSSDWYNRNKDYRDNVLIVMRQSYTSRPLRMMSMGDMDKETFIKGLKGIYTYYNFLTHFE

>HhalOR59

MSDQAIKDSDVLDGLNVKYLKFFGLWRVVNDFRTTGKRNKILRVKIFITLVLVLPYILCQYLSYFVIKVDIQKAIFLNLHLLPGTQICCKIVVFWFKIESQCKLFDLLKKDFLSVPEEMRPEAAEIFKKITRRTNKLCLAAFIVNISIIISSIADPAISVDYILYHTGDMAAVTTGKKKMLGGWYPVPMAETPYYELIYVYEAVAGTLGGFLLAMYVCLFYQVLMCLYAQFTILCLKTSALKIKSDNGRINSSIYKELNEILKEHQKLLSYAKELRSVYNPLVTLIIGIGLFILIIAIFQFLFGGKSDFMFIFKSLQLLVYQCVEVSMFCFGSTYIETASSDLQFAIYSSDWYMTGMKFRKEAQMMMIRATKGETLTALRVYPINVETIMSILHFTYSASAVVSRMAE

>HhalOR60

MLKLNDYADPKDEKYFKTGFKKNYGIWLLYGGMFLGNPLLPVAFILSGFILIYFMLGTITKFYKTDLLCAIENLHFLIFVTVELVAMMSFLQKRAVLVSMYITIGKGFFDYENTLDDECLELKRDAYDKTDSRKRLVHHSFVTVVMSACITISVFRPAVLILFPKENMGNPNDGMIRVALAPMWSPFDNTQWHGIVIVWLLEYIVAWTTPGIVFGATFFVLFSLEELGIQLQILKKSLTNVIQRAERLEQGMEENIKLCLKYSVRHHQLLFEFHDKLNEVISLPGLGLFVSFSIMLCMSGFIFTLKEVPLVSKSVFGLFLLSECAMLFALCYFGENIIELSEEIGDALYNSDWAIYSKVMQNYMLIIQMRSRRTMRLTLMDFMDVSRNTFSNICSTSYSYLNLMNEFN

>HhalOR61

ESNLFQHSGSDRVKLLTMVKIIKELLGDLPRILVKTFLISRDSKKRTGQTFVTISNAIMTLVCISSMYFLGLEKSLEGTASFSAYAFIIATKHVIYYFRQDEIKRMLNIFARLQKEYKEKWEREIFKRDYEDTWNMVYKFCLIMISYQVFYLMFTISVDYIIGNIFPNFPSVRVHLPCDGFIEFFEPRTLGRFIITIPVLMWTAEAITIHVGSETLVFVLMMYTKSELKMIRYRLIIIKKQLNRNKSNRSVNAEMLLWEVIQRHQRALDVLDVMKDTLGLPLAIHYTSVSVTLSVIFYCLMTFDERGTLTAKFNGITAIICIGSLLFALCYFGESLEEENNEINKRIYDLPWYNESIFVKRAIIIMLRQTQKPFVINYRLTAQLSLQTFMQVINTAYSYLMVLKSTVR

>HhalOR62

MAKLNGYTDPEDEKYFKIGFQKNYGNWLIYGGMFLGSPVLPITFLFCATTLIYFMFGTAIKFYKTDLVTTIENIHFLIFVAVEIGAMIVFLRKRALIVSIFITIGKGFFDYGNTLDDECLELKRDSYHKTDSMKRLIHHTFVTVVMSACIAITICRPVIIMMFPKENEGSPDDGMVKVALLPAWTPFDKSRWYTTVILWFIEYAVSWTTPGIVFGATFFVLFALEELGIQLLILKKGLNNVIQRAERLEQTMEENIRLCLKYSIRHHQVLFEFRDLLNEVVSLPGLGLFVSFSIMLCMSGFIFTLNEVPLVSKFVFGLFLLSECAMLFALCYFGEKIIELSEEIGDALYNSDWINYSQVMKNYMLIIQIRSRCTMRLSLMGFMDVSRNTFSNISSTSYSYLNLMNEFN

>HhalOR63

MEPRRPLQEASILEGLATMHMKFYGLWYCLNVTRTTGKVSRLFILVCALIVIIASTYVMFQIIYMFTIHLDLQKIAFVYLIVAPCMQDFYKVIFFLAKMQEICLIYDTLLVDFLESIPKHKMPVVKEIYRRTAKKCNQVCSFAFSGLIIAGSIWLFVPGYDTDDPTSDRKKVLNGWYPFHYSESPRYELVYAYECIMTLWCGGWYCIFECAILMPLICLCGHFDVLSYHIATLKKSDMVHVLGRSSASHLESNAFLNDQLKYILKDYEKLIRYGDTIRETYNLVITIILGVEIGNLTVIVLHLIFEDKDAMFLVKTGTYMSFQLIEVILICFSSDMMGEASSGIREALYCNEWYTTDRKLATSQQLMMVRASVPLTLTAVKMYPVNLETLLSIFQFIYSTAALLSKMK

>HhalOR64

MDPPRFTDYYRWMLKSFLYWGMPTPCLPKLSNPVWLLLVVYDFLTLAVILFALFVYGFTMARGNIGFQDVTLFLPGFVLYSFALYLSLYQFLIKGRLEKIIAEMDALARDIIESKLGDEEFLQVYSDNSKNIINHCQTLPFLYVSISFIFFCSVPIVDWYEGKYRTNFAIRIETPFDYRQPGIYELVVLLMSLALSISTSKQLNNALLFLAFFNTLRSYLKYLYISMGELKRKTIKNDNINNAFSRQDIRTWIKIHQEINRCLQVLLQLFSPVVIVYCLYMMFFLVSALFLQMQEKRNSIYQTFSAFIGVMVMLIQFYMVFNIADHVTFEAEDLANAVYGLPWYEMDKRTKYEVQMIITMCNRPINITGYRTKSLILNRETLAGILTSAMSAYLTLCQMKDAFGPKE

>HhalOR65

MADLDDIIVDERKTRKFLRLVCCLRYTQGKNSISTFNLLYIISVYTLLLIVVVQGVVLAYSTDDIVQKVESIHYVLTIIIVISYMSNELYNNSNLDQAWKLINLAYNEFSKDRDENRQIHVEINVSSVKTNKLFCFLMSSSCVGYLLFAPMRQLFTEEDSSARKLPVPLYMPFDTSDNFGFSLGMAWEAISLFYICGVSTSIHQSFRGIMGRLRGELKLLNNSIKGIHVRAEKYQGKVDDTEKLEAHFQFLVYKCLREDIVHHQMLLEYYSLTKIYLGTILLLFIFLSSIILGAVGFLITKPNSNTEDIIKFLAIVTAELFFVYQLCWEGERVAEESGQIFNNLFNIPWNAYDRNVKGCIRIMLCGTIKPIRLKTSIFNVEASLETYNWVITTAFQYFNLLRNIKSG

>HhalOR66

WLHIMSLQFETTHSFLIRVLRRSGIKMPWVHLDSNLSTFLYSVYDSFLVFIGFYQFIFAAGSIVWVGNFQDMCNLVAIAVTLLTGILISLFYLLYQERLALFVENWNKLNIKILKSRLDRGFKMMYLKIAQSNVSFSKKILFLVFWTPFIYCSPVPIIDVIRGLYRANLPLPILYPYDDHQPGLYELTFCLHVSGLVISVMKKIGNDCFFLSLFKIHTVYLRCLSTSINDSKDKFNMKSDLFIKKKQIVWIQLHTEIMRNANELVSIYTPIIVIYYFNLIMIVVFGLFTQIKNDRDNFIQGFGIGGFGIINIFQLYMMCSSAEVLATESERVALAIYDIRWYQMNRSNGEMLRLMLFMAKNPVQVTAFRARTFLLNKESFIGFITSSLTALMTFTKINDIRQSQSSS

>HhalOR67

MVYLQRLQKSDLFDGLNIGHLKFYGLWNGINDYRSTRKTSCIFKFNMTVSALYVFPFVVFQFICIFIISVDLKMATFVYMNGVSAAQVLFKIIVFWYRFKDQCDLVDLLRVDFLSSIPDSKTRHVNEIYKKNSLRCNIFTILAFTGNVLTIITWTILPGFNTEKTGTGRKKILSGWYPVTYSESPWYEIVFVYEVILICWHGSLVSLYESFLLMLLVGLYSHFVVLGYHLSTLKKNDKAVVKAGVDTKIDEAFNIELKKIMQDYNKLLRYSTLLRTTYNAITTVTLGLDIGVLILTIMFLMFGSSDGLSTFKMMMYFSFALIEITLLCVTSSIVGSASMSIHDSAYSSDWYVADKKFATTAQMIMIRSMIPVSLTALKMYPVNMETMIGIIRFIYSAVAIVSKMKE

>HhalOR68

MDTKNQAKRHNNGPGLVEETSQLFLNGLRRYKSESPLSIPIPLIVAVGNIMVCLMVTGGIVNYTKTDWVQLCINLIFIPVSVMVGTMILKYKRSFSWVNKEIDSWWSYKLLGKETDVMKKNAAEWMAWYGQYYYIGMYLAWVMNLVPFIKCHIWGKSEDPMNNLMYPCWTPLDLGTWWGFVLTYMFQFMSMYLSYFTFGNTCFYLTTAYISIGYQAQLIGLAILSVEQRCRKLSDENKDSSKREEIYESYVKEEIKSCIKHYQQLQRSAKELSNIFSTLAVLSYNIGIVVLSISGIRLTTETDKLMLVNSFVNTCVTLGNQFIISFICELLTEEVEKLRKIIYYSSWVSMPVSCQKYIYTFLMMVDYMPTPVTLTGLKTNYENFSKVVNSAYCYFSLILSMRNK

>HhalOR69

MDPPRFVDHYRALYIGMRTFGMATPWIQKPRSLWRRLPFLLWDTLLVVIVVYMLFCYAYSIMTISIPFQDLFGLGISTTNYICALSVTFYHALYGPRLKKITDNMDSIAAVICANNLGGAERFHKRYATNSKLMSVFTTLSIHFSAYLPFTYFFEVPVTDYFTGKYRSRFPVKINSPFYDREPGVYELVLGIMSLCVSSSIGKKTATDCLFMTLFMIQRNFLYFLSDSMKDLEKEFLTGDNKLFKKKLSIWIKLHQDIRRNAEELVLTFSPVIIIYYVSVIGNIVCAAFIQMKKDNDSMFQSICMSIYVMVALTYLFLLSDAAGRLTVEAEKLAFVVYSSPWYESNKTNTDTLRLVVTISNKPIHVTAYKAPVFLLNRETFLTFVASALSAFVTFSQMKDRFDQ

>HhalOR70

MDAPKFVDHYRSLLRGLRRVGLATPWIEKPTLLSRVPFLLYDGLLVAIVVYMLCCYGYSITTITIPFQELCGLGVSTSNFICALLVTFYQAHYSQDLKRITDNMDRIAERILTSDLRGADHFLQLYKRTSKLMAILTDYSIFFSFTLPIVYCFPVPVMDWMEGHYRSRHPVRIANPFNDKIPGVYELIAIVVACSIAFSTSKKAAMDCLFVTFFSIQSDFLKYLSVAMSELQKELRDEDSPLTRNKLITWFRLHQDIIRNTNDLIETISPVIITYYMTTIGIVVCGAFVQSMKENELFIQSISIGGYIMITLIYYFLLSNTADELTTEAQKLAFVVYSTPWYDMKKRNADMVQLVVTISTRPIEVTAYRAPTFLLNRETYAKFLVGAISAFITLCQMKFLYDDS

>HhalOR71

QLEGNHFSLHQRMKEVDIDSRYFRAIGLWQFVVDYKATYWVLFNFALASVFFVNISVQLMNTLTGGYEFSLLTEKLSVNLTVMESVIKIIYYCAKRRKLYSLSLCFRRDFLICRNHDREVADEVLNTGFSSVNTVTKGFVVMIFTTVGLWNSFPFLRCLTGDCSKWNIMPSWYPEAMDGLPAFIYIFEFFIMVFCAALLYNVNCFFSALALSLSSQFQLLTKSFSSIETNAERRKGCKIYNMNVLLRECLIDHQRLLRLVKEMEDMYNPIFLFQMLTSTFTICLVLVQLNDRTSSKGEMPVAMVCKFFMYLMFGSMELLVYSWGGQIIYDQTGEVHRSLYESGWASASHYFRKSVLIAQIRTLRPEYLTAGKFYAVDLASFTQIIKASYSYFTFLHGSGGSSR

>HhalOR72

HMPAPKFVDHYRALLTGLRRVGLATPWIEMPSLLGRLPYLFYNSLLVSIVIYMLCCYVYSVTTITIPFQELCGLGVSTSNFTCALMLTFYQISYSEELKRITDNMDRIAERILSGELAGAEHFLKLYKRTSRLMAILTNQSIFFSFALPLVYSFPVPLMDWMEGHYRSRHPFRIASPFDDKLPGVYELIMLIMTCSISYSTSKKAAMDCLFVTLFTIQSDFLKYLSVAMTELQKELKIGNSTQVRNKFVLWFRLHQDITRNTEELVEAFSPVVIIYYMTTIGIVVCGAFVQSMKENEMVVQSICMACYILITLVYYFLLSNTADELTTEAQDLAFIVYSTPWYDMTKSYADMMRLVITISTRRIEVTAYRAPVFLLNRETFAKFIVTAISAFVTLCQMKMVYG

>HhalOR73

MDPPRFADHYRPLLGWLRTCGLSTPWGEKPSFALGVIITCYNTLLVIMVCYTLLLYFYSILKNSIPFDELCLLGVGFGLYGCNVAVTLYLFLFQGKLKVIINKIDSIAATIQQNEIGGSEFLQEMYKKHAKLMVVLANNSIYFGYLTPMIFCLSVPTMDWYAGNYRANLPIQIDAPYDYHIPLVYELMVLLLSCCLAVSTTKKAATDCLFMSLFNIQITFLKYLSITKSYIQDDFKSTNKTFSKRKLIIWIRLHQQVNLNIQQLVSIFSPLVILYSVGTIMIVVCGTFVQIMDDNNNLMQSISIGIYVAMTCLNQFLMSRTADELTQEAMKLAFFAYDLPWYEMKRADADMVRMVILRSNRPIQVTAYCAPIFMLNRETFRGFMVTSISAFVTFCQIKDRYG

>HhalOR74

MDPPRFIDHYRGLLKWLRICGLASSWHHQSNFFSRFSFFIYATFLVIMVSYVILCYIYTAMMTSLTITDVCTFGVSGGCYVCGLLVSSYLIRYKDRLKKITDEMDIITKKIIESELGEREFLLNEYNKNSKLMAVLTDGSLYLSFATPIFYCVSTPVLEWYNGMYRSNLPIQTLSFFDEKAPGCYELMVIFTACSIAISTSKKSANDCLFIALFRIQTIFIKYLSVSKGALEKKLLADDSLRGQRKLLAWIKLHQDIMKNVEELIIYFSPVVIIYYVMVVEIVVCGAFVQLEKDNDNIIQSISVGSYVATTVLYHFLLANTVDELSDEAQKLAFVEYCLPWDKMNKKNISMVKFVLTMCNKPIKVTAYRAPIFLLNRETFAGFLLSAISAFVTFSGMKHNGS

>HhalOR75

MDPPRFIHHYRPLIGWLRKCGLPNPWAETPSTPRRLLLFCYDSFLVIMVSYMISVYIYSIASISISFADLCSLGTSGCCFMGSLFITLYMALFRKRIKMVTEGMDSIAEVIFQNELGGARLLQEMYQKNAKLMAILTSNSICISFLAPCTYCWSVPFVAWLSGTYRDELPLPLDTSYNYRLPVIYELMVMLISCSIGISTSKKSAVDCFFISLFNIQIDFLKYLSVSKRYLQEEFNSGKDIYIRKKLIMWIKLHQDINMNIQQLLTVFSPVIIVYNLTMVIIVVCGAFVQIKSDSNNLIQSLSIGAYVAIAGLYYYLMAHTAEELTTQAENLAFSAYDLQWYSLKKADIDMLRMVILRANVPIQVTAYCAPRFILKRETFANFVVTSISAFVTFCQIKDRYR

>HhalOR76

DPPQFVNHYRTLIRGLRLSGLPTPWIEKPSKLLRIPYLLYDLLMLTIALYMLWCYLYTTIQGKLAFDELCALGVGISLYTCSLLVTFYHFLFGHRLQNITENTDRLAAALLRSGLNRGHSLHQLYLKDSSAIAVLTKNTILFSFFCPVLYCLSVPVMNSIAGRYRCEQPLPITSPFDDKKAGIYELVALVLAISNAISCSKKGVNDCLFLALFRIQSSFLQYLSTSLENLQKEFLIESNDWNRKILIQWIGLHQEVLRNIQELVRIFSPVVIIYYLNVIGIVVCGLFTQTMNDSGNVMQSIGVGSYVLVTVLLMLLLSNTAEDLCTKAQRIAFVAYDVPWFEMDKTNADMLQMVINNSSKDIHVTAYRAPIFLLNRQTFAAFIINCIKAFVTLVQMKRCFG

>HhalOR77

MDPPRFIDHYRPVLGWLLKCGLSTPWAEKPSLCRRLLLLCYNAVLVIMVCYMLFLHLYSLTQISSPFPDLFHFGISGTLYGCSLSITVYLALCRERMKTITDGLDSIAETIFRNELGGADFLEQLYKKNAKVMTVLTNNSLFISFITPVTFCWSVPFISWLAGKYRSRLPIPIESPYNYRVPIIYELMVVLMSSCLIIASSKKAVADCLFMSVFNAQITILKYLYVTKRYLQVGYDNDIMVDRKKLVLWIKLHQKINENIRQLIETFSPLLIIYSVGVITIVVSGASVLVMNDNNNLIQPISIGTFIAITVLYYYLLSNTAEELSTEAQKLAFMAYNLPWYQMKKADADIVRMVILKTNRPIHVTAYCAPVFLLNRETFNAFMVTTISAFLTLSQIKDRFD

>HhalOR78

MDPPRFIDHYRVLISCIRRSGLPTPWLEKPHSISRVLLLIYDSIIIIMLLYSVVCYMYSIMTSTISFQDLCSLGLSGGCFVCALLINFYQIQYRKELKHITDTMDSIAEKIIESGLADKDIFVKEYTTNAKFTNNLIKYTLMSMMTTPFIYFLSLPIFEWYAGAYKAHFPVPIENFFNDRLPGVYELIVITIAASISYSSAKKASNDCLFICLFKIQTTFLRYMTLSKEVIEKELLAGNKNSQRKLLIWVKLHQEIIKNTKELILIFSPVLIVYYVMQIEIVVCGAFVEIKKDNDNLIQSISVGSYVALSIIYYYLLSNTADELTTEAQKLVFMEYNLPWYEMNKRNASMVKMIMTMCNAPVEITAYRGPTFLLNRENFAGFMFATLSAFLTLCQMKDIYG

>HhalOR79

MENQKRRMAKINLDIIAEATRKLRIIIGLSNIMESYKLFSLITFINSIGGCCLLIYSFYHFRHSSDDLAGVAYNFGLLNGVVVGSVSGHLGKIKLFKIVDDSYFTYDYQSSIMKKKIEELEKPRAKALKRFFKTLLILTYAAQANLTGLTVLIGMIKKEEIYLFPCWHPFDLSNILSQIFLLAWQQFIILGITFVAFGCWGILYITYSHIKTEISLLDFAIRNISSRAREMMQHRESVEKGEDYDKILLACYKQCTRMCAEHHSEIIRFFTNGQDFIGIFYTTAFISGGSACTFGGYYISSENIELQFKYLAMTTFILTYLYILFWIAEAISGKFFTIAQTVYTLEWYNLPKECQSTLRLMQIRSNHPLFYKLVLGQRVDMEAYMSLVKATYYYLNIIIT

>HhalOR80

MSLQFVTAQRTVIQGLRNTGISLPWVEDDSLLTNALRKFYNFFAFFIVTFQLVDTAISAIMLENFDDKCRAGTVTSIESNPTMLSVYYVFYHVRIKKFLEHSDALSQRILNSELGQKEFFEKSYLENAKKNNFFTKIVLIFVFWTPILYALPRPLIDLYNQEYRKTLPMYLVYPFDDHKPGFYEMTFLIQTLGLVCGDLKKFANDCFFLSLFRTQTVYVKYMSASIRDLGEEFKKTGDLNLKKKLIKWIEIHDHFIRNFNELLSLYTPVICIYYANLICTVVLCIFTQLQEKNFGIIEGIGLGGFFSANVFQLYLQCAANDDFIVEADNLALEIYKTPWYEIDKTNKDIIRTMFLMASRPVEITAFKSPTLRLNKEAFLAFVASTITAVMTFKNMSDLHQ

>HhalOR81

MSLQFETTHSFLIRVMRRSGLMVPWVRMDSSLLAAFYFFYDNLLVLIGFYQFIFSVGSIVRIGNFQDMCTLGIVIAVLATGVMLSIFYIHYQQRLEKFINNWDNLNTKILNGEPQLAKYFQRLYLEITKSNETFAKRILFIAFWTPFIYCSPVPIIDVIRGSYRTNLPLPILFPYDDRQPGLYEFTFCLHVFGLLISVMKKIGNDCFFLGLFKIHSVYLRYLSESIKDAKKKFANNDWFIRRKQIEMIKLHTEIIRNAVELVSIYTPIIVIYYGNLIIIVVFGLFTQIKNDRDSVVQGFGVGGFCLINIFQLYMMSSSAEELASEAEQVVLAIYNIPWYQMSKRNADMLRLMLLMARNPVKVTAFRAPTFLLNKETFISFIVNTITALMTFSKMNDRRQS

>HhalOR82

PDFDEMELYWNRKNLADVWTWPHIFWLNIFGWWGEEAKTEFGRKWLTRFRSLSIVYFACLITSMMIQVYIKFAEGDIMQNLFTIFASGPGMVGIFKFFDLVIHRKILKSIMGRLSGLISEVNDPILNTMTRKAFKKTWIIFLSTLLVFDSVVLHWALRPIIAAILHKEKTRIIESWPVFLDTWTQFFLSYLFQVPGVILLGHSFYIYDNLYFCTSDVILCHFEILKHKLNRLVLNENEKSSKDLVSCVKYHSAILSVCNDFRDATSKVIIWQSINTVIMLCTGIFILTYLGKNINSNALMNLGEVSFTLCTCLYYYCWFSNEITLQCTKVSNAVYMTNWISAKSSDKKIMLITMTRAMHPVMFGGIMQINLSTFINVLKTTFSFYNFLIAVQVSSTKNE

>HhalOR83

MFDPVKIYFNILIKSFSFVTGEEKMSTWTFHKGLLRLLGNDWLVRKGEISLLRIIWLVLYPIAYITSMISITTLTIKYVLQREHPTMLDYTRAMNGVVACIAFVYAICKSLVLKLFGKNLWHLMDMVDDLGEVDEIAEPHRDSSVRHATLYLGLLCLIPATWTVCSMFYMHNIPFPTDWPWGDYTPFRYFISFTIDFIAATYCAVTHSTYDTIFPISAGAICGHIASISAKMENLGRTGDKEKDKKILNECYRLHVALLRISDHINNTFGLVFLIQSIYTVLHACVIIYQVMKVSDITLAVLNTAPILASSYAQFLLFCYYGELLTDYFERLRFGFYNNHWYQLDMELKKTLVIMTLAANRTVRLEAYGITFAGHKTYVSGLQDSISYYLILKTVTTDT

>HhalOR84

MSLFTGMRGPLDQPGRGKWYGHLYSANVTFINISLFTNVLTAGLVCFSPGVSVEVRCFTGFPITAGTLAIIRRIDMYLNSDKYKDLTERYLSLYPDSPELKADVQKYGKIIRSIPRIMFLFTAVPMVTVGLLPAVIAITGGPRDLGVPAIYPFDPAEYIFVFCFLCFFQSTAAFHSTLSSLLFENMFNTFACRQLALTKHLSRELYRILSTAKVDVKGVATFESHDGTLMSKEESNQTVLAELKQWVKNHQQSSRLARDLQDVYSISLFVQFVFTGSILCMSAFVVANVVGGIVKVIFCGFYVLGLMVELLITCRLGNLILYESDSLEAMIEGTHVYALPGNIYKEWLRMILIKAKVPTKIVAVSLFPLDVETFKSLLVTSYSFFTLLKTMQHLDLRDS

>HhalOR85

MSLQFETTHRTVIQGLRLTGIPLPWSDEASRSIKILNNAYIYFVVIINSYQFAFSLYTVMTIDDFEEICRLGIVLSILVNSVMLTLFYALYEPKLKKFYKDSNTLSYNIMNSELEHYDLFKKNYLKVAKSNNNFTRNVLFFVLCTPLIYCVPTPIIDLCNREYRKHLPFIVRYPYDEHRPGIYEITFFLQMLAILYGDVKKFANDCFFLTVFRIHTVYLKYLSASIRTLGQDFEEIGDAVIKRKLVTWLKLHNHLIRNANDLISLYTPVICIYYANLICIVVFGVFTQIKHESGSIESIGLSGFCIVNLFQLYMQCSTNEEVGVEVDKLAFEIYNIPWYQVSKANKDIIRLIQVMANRPVDITAYRAPSLRLNKEAFLAFISRTVTAVIAFGQMSEIHQ

>HhalOR86

MRAPIEESQLLEGMSIFLLKSTGLWNAINTYLTTRRRTIGLNILTAYSIFYALPYVLFQLVSMFVIHVNVEKLTFLFLNSFPCIQVFLKVSVFWYRIEEQCDIFTLLKQDFLSCIPPHKMPKVREIYKQWARYSNVACVMAFASMILCMSSWIIVPGIDGVDDTGAVSKKILGGWYPFPFSRPPWNYIVFYYEMLLMSSHGSLISLFECVMIQPLLCLCAHFTVLGHHISTLKISDVVYSKSKNELHYMNSELRAILLDYDRLLRYTAVMQDILNLLVTAILGTGIVILIIGVLQFKFGTLDPMFVFHFLTFLSYQATEVFLICTSSSALHSASSDICFSIYSSDWYLADREYARTAQMIMVRTYKPSTLTAIKMYPVSVEILVGLFQFTYTAAMVLSK

>HhalOR87

RFVDHYRPLLGWLRTCGLSTPWGEKPSFAWRLIIACYNTLLVIMVCYMLFLYIYPIMKNSIPFAELCLLGNGFALLGCSLAITLYLVLFKGKLKVIIENMDSIADIIHQNELGGAEFLQEMYKKNARLMKVLTNNSIYFGFLTPVIFCWSVPTMGWFSGNYRANLPIQIDSPYDYHIPLVYELMVLLLSCCLAVSTTKKAATDCLFMSLFNIQITFLKYLSITKSYIQDDFKSTNKTFSKRKLIIWIRLHQQVNLNIQQLVSIFSPLVILYSVGTIMIVVCGTFVQIMDDNNNLMQSISIGIYVAMTCLNQFLMSRTADELTQEAMKLAFFAYDLPWYEMKRADADMVRMVILRSNRPIQVTAYCAPIFMVNRETFRGFMVTSISAFVTFCQIKDRYG

>HhalOR88

RKKKTCRNMFSLLKRYKEEEILNDHCQQFCSEWLSFLGIQPHKTLTLHSAFNSLILIFTLAVWTYTYLEMKQGDSTEALHRICLSLLALGLFSIQFFKHEEIMSVAKQIDDCFSYSNSDAKPFFKKRQKDLFTSGMKIYTDLFTIIVFTTWGSILANLYTQQLFVSDVKPPIPMILPIDSKLLYYFVYFTETAFVVIASIADVLVAHLFMMFTLQLTANFEVLCLNLNSMDRFMKNDLAMLPDSEIIPKININIRHHQEIFKSFNSLKKLFDQIFYILYFSMMSAIAMCRTLLVGDIDIKSLLPLFYLETGYIHIFCHFADMLAEESANVRLAAYSTPWYTFSSNVCTSLRIMVIRALKPPKLYFFIGGSDVSCATFTLILNASVTYFCISFMMENQN

>HhalOR89

MAKSYIQWPWEKENITEIWKWPIIFWLNIYGWWAEEASSTFWRKWLSRLRVICFINASACFWSMIFAIIVKFSQGDIMVNLFSVFGAGPGVVGTFKIVQCIRHRKPLKKAMDLLDVMMSEIDEPELEPIVRNGMKRCWIAFFLCLFFGSCISLHWLSRPLIILLFYGERTRIIDTWPVLDDDWFQWSITFLFQGTNVCLCGHTFYIFDNVYFCISESLLCQLRVLKYRLTHLKLDGSVGSDAALEICIKQHTQILKVCDKLKFASEGVIIFQCINTAIMLCTGIFILTLMEHINFNVLLNLGEITLVIIIILYFYCWYSNEITFQCSELATYAYMMDWTDGTFDQKKKLLNLMTMPMHPVIFGGIVEINLNTFINVMKTAFSYYNFLAAADAGGDHAK

>HhalOR90

MNGEDEKENVCLYESLFLDGIVLTSSKPHIKLPIPVVIFLGLVMIITVTLHAFMYAETINALDKLIDYGFPAILVSSMLCTLKYRREIHNLSRDVDSWGTFRYLEKETEDVRMNAKLLAQRFSRFYMFTIIMGTGAYCLAPLGKFLFLEPSDPADLLIFPCWTPLPIDTNWGFYTTYFCQSVIIICANIAFSRVFCYKIICFYTIGQQMRLIGSALSTIEQRAERQMREKKNLIVKGKTNIHFIMEQEIKQCARQYQTAYRAVKDTTSIFRPVTSVVYHYGMWVICATGVKIAIEEQLFMKVQILMLLNVILVNQYLYSVSSENLKNEVELLRIVTYSCPWYKMPIHCQKSILLMITSMSHIPSFNTIFGTETNMENFGKILNGSYYYISLLLTILSD

>HhalOR91

MARIMLENIKAATDKMVFLVTLSGFYLSPRHPLVQELFLFTNGFLATSLLIYSLNYYWGRIEKTGAVAYYLILCADVCVGSSMGYFMKSSVYSMIKGSIWSHDYESSIITIKRRELETARERDIKMLYKLLVVGIFYMMLDVILMAFVEARINNDLFLLFPCWIPFDMNQISTHISLAIWEIFLVLMVALCMFGAMGIICLFYSHISTEFNILEYAMDKLEERALEMASDYGGFDTRNAEVMKLCYKRCLCMCVDHHAEIIRYFRNATLFLKVSYFAAFGTGVVIFTFAGFFVISDNYAVKIKFTLISLAQLFFLYIISLIAEMIVEKSMTVREKTFDINWYDLPMECQTILVVMRTVSNKPLETKLLSGQTVDLAGFMSLVKVSYSYVNMLLAITH

>HhalOR92

STLPMNTERAESLVYLLKIGGLWINFTNHQFGSLIAVFQIFRLIYLVVGWFLMINVIFLKGVKFLLTPSAVFIPLGFNVVGTSVLYFCKIRMMEKLILQCDKNLSSYNEYWEKKLIQTDTEKVKYIANCFSYGLTCFGIVYTIMHVIVNIVRSITGNPERYTVPLPFDGYLDDTEERNFNFYIYTLLSDLWLAFGVPNTVAFQCTIFYVVSCTTTEINIIKEYLRILQIGQNNNATILPDWNLNQIILKHGQILRFNDLQNQSIALPTLVQCRIIFTLSICFVMFLILQLFGKNTFVMLLAAVFILILFALGLVLCSAGEYLEEKSDELFYAVCSLPWYKQSLTLQKSYFLLLLQTSKTLVFDYAFTSSLNLRCYMALVNNAFSYLMIMKSLSPADE

>HhalOR93

LIPLKVLLKGTWPAMERFCTSFLEYFEGDKLRLGFAPQRVMFKVMGCFWWKKPSLLQKMYSCGIWIGIQIVSLICIVLYILRTEMKTAEDFTKIINSLVMLIPFLMGILKVGILHINSANFKAMVDFLESFPHDGTIFPAGYKFFMNISWNLITIACWSINGFIRGNLVWYAVLPWDTSTPTGYKLGIASQLVSAVPAGSTHVMIGSVMVAAVERLRPQISKLKVVFSKIGPNHEDNKHIIKEAVDLHSAIKRGVNLINNAMGIIFMVKVLSLVPLICINAFVITKVGDIHYIVINLLPMSLCVCGELFMFFGSGQILATEMENMDHVCYDNEWYLAPVEDRRKLSIIIECSRKQLTINAYGIAYANNGTLLAVVQQGYSYFLFLHTMADIFDKN

>HhalOR94

MAKIKLESIEAATKNLKQILTVSTHILSNRFYSLLTFIHATFGFCLLGYSVYYFRNSPEELAQVVYYMGICVAVIVTTIIGYVPSYRSRNVLEDSYHTYDYQSEIMRKKIDEVKESCVKSLKGFFKTIVFLMVWVSFNLTAMTILVGLIKQEPIYLFPCWHPFDINNIIFQILILLWQQYFLSTMIFMAFGGGSMLFIPYTHIKSEISLLKYALKKVEARAHEMARTRKASNVGSVKSKVLSECYKECIKMCVEHHIEILGYFYRGKRLTGIIYTTGFFSGVIACTFGGYNINSENLALKFKNLAILFFILGYLFAMFWIADATTTEFLTIAQTVFEVKWYELPKECQSTLQFMVFMSNQPLFYKLVLGQKVNMEAYMSLVKATYYYLNFLTA

>HhalOR95

AMSDQAIEDSEVLDGLNIRYLKFFGLWKVINDFRMTGKRNKIIKIKVFITFAFALPYIFCQSMSYFVINVDLQKAIFLNLHLFPGTQMCCKIVVFWFRIGSQCKLFNLLKKDFLSVPKGMQSKANKIFTKITRRTNLLCMAAFIVNASIVILSIVDPGISVDYILYHTGDMAAVTTGKKKILGGWYPAPMAETPYYEIIFVYEAIAGTWAGILLAVYVCLFYQVLMCLYAQFTILDLKASSLKIKPINSRRKYKINSQINSSMLKELYEILQEHQKLLSYAKELRSVYNPLVTLILGIGIFVLIIAIFQFLFGGKGNLMFIFKSLQFLAYQCIEVSMFCFGSTYIQTASSDLVFAIYSSDWYKADIKFRKAAQMMMMRARKGEALTAVRMYPI

>HhalOR96

MQWPWKKKNLSDVWKWPLASLVNLFGYWAEEAKTESNRIWKKRFRALTMLFLPYIMMSMVIQGFVKFAEGDIMQNLFTIFAAGPGMVGLGKFLGIVFYRKQIKSVWDRLSAMISEADNPKLEHIARNSFKRTWILFIVYLSIFTASVTDWLMRPPAAAILYHEKARIVDTWPVFLDTWLQWFLSYLLQFPAIVLLGHSNYTYEILYFCTSEVILCHFKLLNYKLKHLVLRDNKESTEKLISCVKYHQNLLSVCNDFKNSTSKVLIWQFLNTVIMLCTGIFILTVLIKQTPYVAVINLFEVCTFEIMSLYLYCWYSNEITFQCSDVSNAVYMAEWINAKPSDKKTMLITMSKAEQPLLFGGILEIRLETFINILKTSFSFYNFLLAFQVNSTEK

>HhalOR97

MANIKLDVIEDASRKIRLLLSLANIILSYKLHSLTTFINSTLACCVLAYSIYHFRNSPEDLAGAAYNLGACASVPVACVSGYLAPVTLRTIVDGSCCSFDYRSSIMRKKLDEVENKRAETLKGFFKTIEYLIYYSTINLLGLTALLGIIQREVVYLFPCWTPFDQNNFFWQITMLLWQVYLIISMTFMAFGGGVLFYISYSHIAAEVKLLKYALKTVEERAHEMAKSLKRSQGEPYARILSFCYKECVRMCAQHHSEIIGYFNNGKDITGIYYSSGFLCGGVACTFSAYFIISDNFALKVRYLGMTLLLLGYLFVLFDIAEETTNELQTVAETAYAIEWYKLPKECHSTLKFMLLRSCEPMFYKLLLGQRVGKEAYMALVKATYYYLNMMTA

>HhalOR98

MDPPRFIDHYRGLIAVLQRCGLPEPWLSSSPSRLRTLYQLFVVFSITYIIFSYVYTFIISSMPFQDFCAIGIAGGCNVCGILVFIHHKRYSTRIHELTDTMDAICKQIMTSDLNKKDEFLKEYERNSKIMTALANYSFYLNFTTTCSYFLSLPAIEWYTGNYRANFPLAISNSFNDRIPGVYELIVLAIAGSTSISTVTKSTYDCLFVSLLKIQTTFFKYLYETIDNLKGDVQSQHHFLEWIRLHQEIMKNNEELLQTFSPVVIIYYLLVINIVVCGAFVESKKENDHLIQSISVGSYVVVTILYYFLLSNAADELTNEAQKLTFAGYALPWYQMKKRNSSMIKLILTMSNKSIEITAYKAPVFLLNREMFVGFMVAALSAFLTFCKMGGS

>HhalOR99

RQTVSSASYREYYSKKVDMSVPPTRWVRSALEVSGMLAPSEYPKLYSIFSSYSTAATAYCCGASLAYFIIMEGATTEKLQSIQITLTILSGLMKLCNAVLQQNKLNTLLLDFDGLWDEFYEEQLNREIMDKSEKFCRLLFKLYKYFVTVTPITNIVMALIGYLATGADQLILQIYMPFDNKKYYIPTQIVQMMLVISPLLANTTSFLLYLVASEQLTTYMRVLRRKLREEKICNQTIRQHQEVIKLLNNTNELFSWLLYFETTAISVECSCSAYALYKVHIKEGQRENVFIDLINFVFCFFTPYVISYCGSKITTESNLLFREAYNNAWYEHDLKAKKDLRILMLGASKILNLQYGNIVVFGMEHFKSIIQVTFNVFNAIIWLEHADQHH

>HhalOR100

MVKILKSLLQELPGVLEITFVISRSPVRRFIQAFVTITNVVMSIANAVSLYFLGLERSLGGTASFAAYGIIICIKHAIYYFRENEIKQLLGFFVEIEKRHKKGWEKEMFEKISKDAWNVVYKYWMIILPYEILYINVLTLLDFVIGYVIPNFPSIRVDVPCEGFIEFFEPRTLKRFIITVPILIWTMASMTVHLGSETLVFISIIYTKIELRIIRKKLEMIKNYLEKKEDDYKIKSEKLLWKVIAQHQRTIEVLNNMKGILGLPVAIHNTAISVTLCVIFYCLMTFDERGSLSIKFHGITLIMCCGSLVLGLCYFGESMEEENNEVICSIYDLPWYSEGKNFRRTVAIMLMQAQKPFVIKYRGLATLNLKTFMQIMNASYSYLMMLKSTV

>HhalOR101

ASLVTMLPRFSGDKDLADVLRLQKISGFWFHFNEKYVIFLQMLRFLYYVTFFIFANVIAYRIGMKNALSGSYFLVSWGFYGIPQLITYMLYNKKANNVIERLKLLYGKRTEQWQWDMFHKNSKFVWIIIRFNLIAAFLFIVVYYLPPLLNDLFRLIFNAGGENMFSFPYPLKEIQSESRDFKYYGIFFVSLFWTTTTIFYGVGNITFHPIVCSYCCIEIQILRKTLEDGRKKKLLNDKTFLKKIIANHNEILRIVEDAQVVLGPAEGAQIATGGLLLTAFVFALMEIKGNIILLIAHISCLFVMTLLNFSICFFGEMLQLQGDKLFECLCGLPWDEMTPKTRKNFNLILLQARKPICISFKGLLPVNFESFKFMLNTSYSCLMLLTTMKE

>HhalOR102

MVKVIRSQLGDLPTVLEDTFLISSNPRARFRQTLIMITNAIMSLTCAVSIYFLGLERSLEGSANFAVLTVTNAIKHIIYYFRKNEVRELLNVTVKLQEEHKKEWERKMFEDGSNDAWNDVHKFSVILTCYVIFVLALPSILDFVIGIFFPDAISIGVHLPCEGLMDFLGPRSLERFVFTVVVLLWCFEAITIDIGTESLTFIPIMYTKIELRIIINKLLIIKNLMNERDIGYRDESEKLLREVISQHQRILEILNSMSKTLGLPLAIHNSTFAITLCFSFYCIITIDESGSMAAKVQGTSAIIIIGSLLCALCYFGEQLETENQELLKAIYDLPWYNQNLNFRRAVLVMIRQSQKPLVINYRLISNLNLQTFMQIINKTYSYLMMLKSTV

>HhalOR103

MVKYIRSLLGDLPTVLDKTFLISIDSDKRFKQIFFTICNAIMTTACVGSMYFLGLEKSLEGAAIFSALSTTISVKQLIYFFRLAQVKKLLYTLKKLQNHHRETWEIEMFEAGSVDTWNAVHTFCLTLLCYLMLFLVLSAVLDFTIGIIFPRAPSLLVQLPGQGFIDFFEPRTLEWLLVTTLFLMWAFEAMVIHIGTESLTFVPIMYVKIELKILRHKLLLFKEELEKLGKNTRHADKLLTDIILHHQRTIEVLNVMKKTLGLPLAVQNTAFSITLCFNFYCIITFNEGGSLAVKFNGVLVVICIGLLLFGLCFFGESLEKENHEVLNCIYDLAWYLQDKNFRRSVLTMLRQAQRPYVINYRRIANLNLTAFMQIINTSYSYLMMLKSTV

>HhalOR104

MGWPFNRKEISEIWKWPHIFWLNIFGWWAEHAKTEFLRTWLSRYRFILCVYFTIVTSMMLIAVGVRCVQGGILDNMFTVFGACPGIVGIFKVYCLILFSKPLKNAMEGIDELFLDSKDPNETKMIRNTLKRVWIIFTFYLIMGSCISMHWVVRPVISAIFFGEKTRIVDTWPPFIDTWPQFFFSFCYQLPMILGLGHAFYIFDNIYFCISECILCNLAVLQYRLNRMKLKEKQGRSVDLVHSIEYYSKILRTCTYLRDSSSAVIIFQCIITVTILCSGVFIITMAEHTDVNVLMNLGEESTVVVFVLYFYCWYSNEIRFQCQDVCNAAYMSDWTNGTEENKKSLLVLMTRTAKPVMFGGILEIDLSTFFTVLKATFSYYNFLIACVSNK

>HhalOR105

MIGLRVGDLVHLLENTFLYSEKSKTFRKFLNMFMGAYSVGTLVVCLLAAIEGGFKKTMEQTAFYIVIGIITSAQQVYLLCYQDQVKKMVDILKGYQEQHVGKWQTEMFERDSLGVWKVIRYYNMVMGSYNVFYFSLPFVVDLVGGLINPNFPVVRFPLPSQQYVEDYEPRSWGNVLYSLIGITTGILTIEMNIGIEALIYISIIYTKTELNMIKKKLSIIKEVMERKGDNYRMDVVRLMKDVTRHHQSTLEGLDMMDNILGFPIAVQNTIYSICFCIDLYVLTIFDSEVKPGPFIVSIFSLTMMAIILFLFCYIGESLENTNNEVFEALYDVPWYQEGPQIRKHLTMMIQQANKPFVINYHGMSNLNLHNFMQVINTSYSYFMMLKSTM

>HhalOR106

MLGIKLASYETDPAKLTVFRNCLIYAGALNDGTLKSKFFIFYRAYGLIVGFLQYAIMATKTTEFMTVVEVFHWMCDFTIMTSMTMSCLYYNPILLRMEARIKAGFFDYGGPLTPEQIKVRATMNRNSQLMSKLYCSLCYCGLVATYIHVFFAKKEYLLPYPMWFPHEIRNMYQYVLTLIHVFIVAETMTICAFSQLSAFVALSSHLIAQYKILIIAIKEIDLIAGTKATPEEKILKMHAKLKTCVKHHVIINKYFDDLHKLFSIPLLTTAIFICLAICTLGFILLSPNVSVPVIGALIILFLPEMFIIVVYCVYGQKVADVCEEFGHTIYYSDWYTKPLSVQRDFLMILIGSRKKRQLTGFGLHEFSMKGLSEIIKATFSYFNMLRAMN

>HhalOR107

MAIDVFRKHRYVLRFLGIVLPGELADSWLRILFHIWFRYLSVCLPVLTLCFLFGITQLHDQYSVTACMYSFAGVVSTTSASFKLILLYSHRYDIKELVDFLKDFKADTTVGRITYVLIYFYEFLALSFRVIALIEIFMKAKLKLLMPFWTPFPRDNVMVAICTYFFFQGTAIIKTICNFFIDTLFLLISNQTCHRMFILRNTFRIIGLPEEEKLERLKHKHMLKLPPGVRPTDSNILKLCVEEHILLLKVVDKFTKIINRIFLPQSFNAFSSIVIVLFLVSQAEDMVRESFKVIPWTTVIFLQLFVSCFAGELIKSRCFDFFDSSYANQWYNCDQKIKSSILVIQTYTSRPMIIRGAYVFELSLHTFETAVREVFSTFTVLYQLFNTK

>HhalOR108

MLERIKGKKNLTDLLLLLKICGLWFDFKGNIVGVLQKFRYLYFLTVFIFMNISAFQNGIKSAFSGTFFLVSGGFYVSLQVVIYYLFRRKTWDIIEKLRMFNKERNEKWQWKIFDEHSNVVWIVFWLYGVAAITFILVYYTAPLLYDLCLLIFNTDDDGYIFSFPYPLEEIRKNNRDFVHYSMQLMSLFWSLLLVGYGMGSLGLQTIICSYCCIEIRILCKTIEDGGKRKLLNNKQFLKKIIKNHNDILSVIADAQEVIGPAVAAQIALGAILLAAFLFSVMEIKGNISLTVTYIILFNIMASLNFTFCFFGEMLQTQGNELFECLCCLPWNEMTPKVRKDFNVILVLARRPILISYKGLLPVNFESFQFVMNSSYSFLMLLTSTK

>HhalOR109

MWDWRQLNWLYYFGWWPSAAKTEVGYKINRVYGIALFIWDFIQIGPEVAALYIALSNGSLKGTVLNLNTVLMGGVCFMKITGILLNEANIKWIIAKLEEMENRGKNMLGLNEYKMIADYRDRRCKLLVIIVFTYLSGLVQWIVRPIYDICNGRTSLIIEAVIPWDKDTIYGWTIVFILQFVHLTTAIMALIIIYVLYLSIMEMILCQIDVLHYSLKKLDFSPPGADYHSSITLRYCVKQHQDILSLCYRFHKVVNVQLFVFMMFSTVVLCLSVFELSSIKDTTLFKVSNLLELTLNTVFLIFMYCLYCHNTVDKLTEGTLRAAYKNNWYMGREEDKKSLDILCTMSIKPFEFGFIIPVNLDTFITVLKSAFSYYNFLKAIADEE

>HhalOR110

GMDVFRRHKYMFRCVGVYFDGELGRSGRVVVELQRWITTFLLVYMHSFGFILGVLYRHSEFPLIPTLVMCCGMVSGPSTLLKHMMLHRRRGQVKELMDQLNGYKVEGGVSTFTKYLIYSYEGMALFFNASSILKNALENPDLTVPYPAWEAYEINTQGRRTLHFFTFYGAIAICWLVTASVDTVVLHLTNQLCDKLAILNNTLQLIGVDQEQRESVLTKKKVLPLQDYKDETILKTSLEEHCHLMRLTKSLSQFLNEVFIYQGVNSMAVLLVCLYIFSNSSDLVNDTVAFSGALVVVFLQVLTSCWSGELIKMRCEQIHFSLYNNQWYNADHNLKRSLFIMATYTAKPITLRGAYVFELSLQTYETFIRQAFSFFTVMYQFLD

>HhalOR111

MWDWRQLRWLSYFGWWPAAAKTKRAYRLLRIYGIVLFLYDFLQLGPELIALYLVICKGSMNEVVLTLNNNLLGLGSAWNIGCVLYNEKNIMWITSKLEEMELRVKKMIGNEEYDKYAAYKYKLCRNYVLTIFIFLAGLIQLTIYSLYWTYHGHVSYIVETWVPWNTDNLQSLIILYTLQVVHSYTGLTAYATVYHLYMSIYEMILLEIKAFHIALSKLDFSPPGVGSHPPVSLDLCVKFHQDLLLLCRKFNETINVTLFLFIMFSSLTLCLSVFELSLIYDRGKLTMLIELIMLTMSMTYFYCVCSQDVVEQMTVGTLRAAYDNNWYVGCAKDQRALHLLCTMAKKEFQFGFIIPANLATFITVIKSAFSYYNFLTAMDLQE

>HhalOR112

MERFCTRVLEYFEGDKLRLGFAPQRLMFKVMGCFWWKKPTLLQRIYGSFFWIGLQLLTLISVISFILNTNIKTAEDFTKIINSLVMLIPFLMGILKVGILHINSANFKAMVDFLESFPHDGTIFPAGYKFFMNISWNLITIACWSINGFIRGNLVWYAVLPWDTSTPTGYKLGIASQLVSAVPAGSTHVMIGSVMVAAVERLRPQISKLKVVFSKIGPNHEDNKHIIKEAVDLHSAIQRGVYLINNAIAIIFMVKVLSLVPLICINAFVITKVADIHYIVTNLLPMSLCVCGELYMFFGSGQILESEIEDMDHVCYDNEWYLAPVEDRRKLSIIIECSRKQLTINAYGIAYANNETLLAVVQQGYSYFLFLHTMADIFDKN

>HhalOR113

MSNIWPQLSKLNWFGHWPTDDGLKGGLQRTASYTILFWTLCLFGAELFSSQQTFLSGDLIGMLGNLNEFFSGLQCYLATLVFVVYRQPLEEAMRLLEELTQEVTTELEEVAMEEFSRRRKNGRRAYYVFFGTFVMVCYWAVRPLLSMAIYGESSLIVHSWVPFAKDSLLGFFGNFMFQIVPCLSFCLGFCLFASIFVFLSEMFLGHFAMLGKKLNSMDYGKEPLESKLANCIDHHTRLLKICSMFQDITSVPLLTQCILYVLGLCISMFELSDVQGASSGRLISVVAEAQQVVLFLATYCWYGNEITIQCLELMRPGYMTNWYQGTRIEKKMLLNIMTRTTRPIYLGALIKMDMITFINVLKAAFSYYNFLAVVAAVNAEE

>HhalOR114

MKFNAFRKHTICLTILGVRPPGGVKWKWLGVLLDIYLIVQLFCIATWGYLFFQGAFLYLPDAFMDRIVGVSAAVSVLGSLAKLITFRRKGHVLKELFDQLGGFPVNTARGRLVAKVVTIYEVSVAAIVVTHNMHIAIDPQIHRSSPYWTASMTSGTKQDIIRDYLFNLDSAIVALSANVIGDTLFLILTNQICHRLLLLAETVASIGKGIRPLEGLSVPEDATDEEVIRRCVDEHNLLLKMTKQVEELYDRIFLVQLVYSCSDACLTLFTISQYEDFVEAMNQLLPQFISLFLEIFLFCWGGQMINNHFERLHLASYDSEWYSLDEKNRSSILVIQTFTGRPVNVTSTSIFHMNFHTYETICKEVFSYYTIMRQLFSQK

>HhalOR115

MDTFRNEKIALRLLGITFPKDLSSPVLKTMVTLYFWLQIAAEGYWAYLYFRGAYLYLHEHFLIRMVGLSAAISVSGTIAKLIAFKLNSGLLQELFEQLGGFVANTDRGKLVKKTTILYESAVAAILVTHNYGYFINSTPTRSTPYYSRYQTGPYEEVALDYWYTLYCVFVTLTGNVMGDTVFLLIINQICHRLLLLSNTVSVMGKQEKNSTMEKMDMRGGESDQEIIQMCVREHNTLLKFKNQLEELYNYVIVVQLAYSFSTICLTLFVISQEANVMEAMSELLPQTISLFLEIFLYCWAGQMINNHFEELRLAVYNSGWYDSCREEDRSSVLIMQTFTTVPVEVTAVSVFHLNFQLYETICREAFTYYTIMSQLLGQ

>HhalOR116

KDPMRPKKEIKDPILFLERLLEWMGVVSIAEDQKPWYYTPMVVLQHISHVNGLLATSLYVLGDNPFFSKMESAQYFISLLHMVAKYYNLHYNNPPYRRLIANAKYLWQEAKKRPRMNNLLAELSQDVDKKMKFFFFIFAMVSPASCLVTLIANLMQEPEERGKPFVVWDPVPESWYWTSCFIEGWCMMVVLSMLGTTLVACYGNCLQAATQMRVLQQMLEDSPLDLRACVQLHQNILRYIEDINDYFSGQMFLEIVFSSLQTAIRGYVSLKFLNAGNPKVISSFFFLSLCLLGPFIVCLSGHVITDSKEKLFISAYNNAWYSASPREKSSLVILLCQASKIKRLNYKNLLDFNMERYSVVVQGTYSYITLLQGADL

>HhalOR117

MERDEPYAVFYKVLEYNGIKENEHSKHRKWFTVLEGILLISLCFDNTFPLIYVIEDHPFNDKAESLQCVTSMLHISAKVIYYRFRGREKLKRLLDDSVKIWKEMRNMKKYEDYLDETWLAINKRVNVITGMFSLCTPIYCLIAFAINLNLDPEDREPLFKIWDPLKYFFKIEDYFFIRMFYEFMMFSYALSTIGTLYLIYLIPSFMAASQIRFLIKMLSSKDLNVTACIEFHQKVLKYVKDINSLFTVHMFFEIILATLVITFRSYQLIMIITNYDHGAVVVSYYLILCFMVPLLVCYSGQMITDASEDLFHWTYQNEWYRLNTKDRKSLCIMMTAASIPLSLCYRNTVTFDMNRYMAVVQATYSYITILINNN

>HhalOR118

MVMIPPFHYTRKQMVMLGILELPNGPKFLRNIITVVIIALSFNSNLSSFLYFLLEDKPFFEKTESILNCIGILHVFSKALVLPFKRNVLLSVMSDLDEMSVDSNRFQEFRKGYNLNMMLSRKIPQTVLLSMCTLMMCGTIVSIVRYTTQGTVTPPVQLYIPFKDNISLTILYNYFLVLPTTFVFSVMISFLISLSLNISIQISYLIMKLENLGMDLSSKEIDSCIILHQKIMRVMTKVNSLMSGLLFFEYLLTSMQCCLSGYQLLADKKNDGIAGFFYHCTFFAISVTFSSVNCYCGDIIKLKSENIFEAAYCNNWYSLSNVERKKLLVLQLASSKPFTLSYRHLITFDLALYGIILKGAYSLVTVLQTMETV

>HhalOR119

RYLKFFGLWQVINDYRTTGKKNNIIKTTVLISLLLAVPYVVFQFLSYFYIEVDIQKATFLNLNSLGAIQLCCRVFVFWIRIDSQSRLVNLMKKNFLVIPIQKQEAVDELYNEISKTTNFWCLTYIIVNISVVILYIADPGVSVDYILYHTGNMDAVTTGRKKILGGWYPFPMNESPYYELIFLYESVCVLWAGILIAVYFCLFFQVLCCLYAQFVVLGYDAQTFNFDLENGEMLHHYHLRLSDTFRRILQDHQNLLRYSDELRSVYNPLVTLTLGFGILVFIIGALQFLLGETMSPAFMFKMSKVFVFQGVEVFLYCFGSSFIEAATELRLAVRHLQQSVVQGEQRDREGSADDDGRSEKWCSADSYKDVSG

>HhalOR120

AQDEEAAGLTLQKALFKLSGVFLWDPPNKMNAIYPILPMFFINIVAILNFLQVFRYSIHTLHDFTEMILTLIVALVFASSGLKLILLHWSKNYIKELVDFLESLPRLESYKKVKHMEGVCMLYMYILIFTAIPKYCLGIMKGELPFESAWPFDTKTTVGWWIAYSTDILSANFCWFAQSSLDTLLVMSVTLMAGHLARLRKILSSIGKDTVMDERIIKTAIKLHVDLLHGADLVNSSYGILIMFQSLYAIVHAVIIIFYLVKVPDILNAVALSFMLMGAYSQLFLYCSSGQLLTTEFEKVHGAVFDNRWYRSKPSIRKSMVMMGIRSQKLVIVKNYRIFTALHTTFLQSCQESFSYFIVFRTLAANIKLS

>HhalOR121

LIQEPILKMAIDYMKLTRKLYVLAGILTEHNTPTSWKVKLISNLAVCSVFLFNLYAFCNGCVVSGLTKDCISHTIVFIVVHIQSYIKFGILVTMRGTRMKYILDFVEGYVFVNDSYAKHIYLTSFYIIALTVLAYAIHPLISHSLPFYYETPWGSESFSAFASSYFVMFFDLYLIQFVSTINDFTYLMCADAICYRLNRVKSLLESIEGEEDEGKLIRAIKEHQDILRTLNMLADTISPNFFMQVFATLSIVIFSAFTAVTSNESQHFSACLSTLATLLTYCWLGQMVTNATEALHFAAYNNKWYNCNRRMKMNLQILSSFTREPFEIRGCSIVRMNLQTFKEVVTDSYSYLMILITMDDTS

>HhalOR122

YPPLQDSNIIDGLRVGYLKFFGFWKIINDYRTTGKKNTFMIFKFYATFIVSTPYILPQLFSYFAINMDIEKATIINLHCLPAIMMCCRVLVFWVRMDSQCKLYNLIQKDFLHIPEYKMAKASIIYKKITRNANSLCILSFIIDVTLVLTLVSIPGVPVDYILYHKGSMFDVKTGRKKILSGWYPLPMAESPYYEIIFAYELVCVLTVGLFQPLYISLFYQILMALYAQFVVLGYHVSTLNISRNSDTRNKIASEKTESGINEDLYKIIQDHQRLLSYADELRSVYNPLVTMNLGLAICFLIVSVFQYQSGETGDMAAVLKALLFLGALLVELFMFCFGSSSLQTAIELESSACSL

>HhalOR123

QIFRLVIFVKSVSLWILSAYIVGAADFLKKIYAGIVLCTPYMVIQTIFIFCNGHHLQSFVTTMANTIAKRNEPWQKEILNNLLNPLWKIIRISTFYSLLLLTVAFYGPILFDTLSTVFTSAEPLNLNVTIDGLLPSVKKGNFFYYFLQWYNAFLEGLGLAHFIGLVSILPLLVNYIRVEIKILCRKIDEYSRRGVAEQEALLREIIEDHINIMRLTELTNKFLAAPLILQNVAAAAAITLFTYAMTNSISQGYDNVTALLFNLNSPIAMTMVVFLSCYSGEMLHLESLNIYDGFCQIQWYNMPKKRQKDILIILVQVQRPLRIQFRGNAVDLRAFKQVINATYSCFMLLKSFA

>HhalOR124

MKVIDVLSHHRVYRWFLNYLAVACIFIDREWIWKKYYVFMTIFSVFGSGVMLYSLIVFIRDIDSVAVIMHHGTINIDIIISTQVCFIHRHTIRELVKGMSKSFDYESPIVSQFLDDLQEKRVKEMEKVFKYIMFTAVFMQINLIIFSIVEKYMKNLDYMLLFPCWFPFDLSYLPYHILAYFWQHINVEAIGLLICAGMAFSYIVYSHLTSQIILLKFAIEKLKVRAYELACSTLGDNTKEIFEDRLRKCYIKGTIQCVKHYSMIIDYYTQAKNLFKILYLIIFLTGLIILTSTGFALVSENTSLKIKFIGVNAVQMVYLYIFCWLAEEISDLGESIRLSIYGIDWFEMPK

>HhalOR125

ERSRLGLGEYLSNQLGMEGEAKPNLVDRDVVYNFEQERELLMMISCYKVSKSSKLNHFLSLSYVSIVWLFVLFELCMGLYSVVLTIDSPKTQLLETLHTVVLTFYVIAHMTNRLQSGFDAALEIINKGFYTYDENMGETHYEIRKEYVRRIRLVNKWFRIIIIYSGISFLMFNTAKKYLENVYKTEPSKIPINPYFPIPYFMPFDTSTVVTFTSAYLLNVALEFFICSVTICIDEIYVSLIEQLKAQFVILNLSISNIVERALRRYQDGKAGSVPNVEELYQQKEFQDCVLQCLKENIRHHHALLRFTALIRNYVQRTFFPCRDNGRSGPCSGGVSHY

>HhalOR126

ALQMCCRMLLFWFHMDRVCRLYDMIRKDFINIPEYMRDSVRELYIKTNRTFNVTCLVIFIWNAGIEFIFIVFPKVSVDYVQHHTGSMAAVKTGRKKILSGWFPCPMDEYPYYEIIYVYEAFCLLWAATLLNVYFCMFYQVLMCLYAQFTVLGARLSNLKLDFPDFGLSKRHKNIIQNCNYNLYQELYESLRDHQKLLRYTDDLRKFYNPLVTVTLGIGILLLFMGAVQILLGKTSDPSFLFQLFQIFSFQFIEVSLFCFGSSRIESASTDLQFAIYCSDWYKADVRFRRAAQMLMIRTRKSSTLTAIVMYPVNLETLGAIVQFTYSAAALMSGMVN

>HhalOR127

LWKVINDYRTSGKKNKIIKFELAITSLLVVPYICFQYLSYFHIDVDIQKATFLNLHSITGVQMFCKMLVFWFRIDSQSKLYNLVRKDFLDIPLHKRTVAKEIYKNITKKSNVFCNAAFLVNASIVTIAIVCPGLPVDYILYHTGNMDAVTTGRKKVLGGWYPFPMAQSPYFEAIFVYEAVILVIGGIFLGSYVCLFFQVLMCLYAQFAVLGYHLSTLKISPERGDTRVTENRRDDSKMYKELYGIVKEHRKLLSYANELRSVYNPLVTMILGMGLLVLIISVFQFLFGSTGNPMFIFRSLQFVAYQGIEACMFCFGSSFVETAAELGPAVRHLQQ

>HhalOR128

DYLQGGEMAKMKLEIIEAGTKNLKLILTVSTHLLSNIHYSLFTFVHATFEFSLLGYSVYYFRNSPEEVAQVLFYMGLSVAVMVTSITGYVPTYRTRELVEDSYCTYDYKSLIMRIKLDEVKGQCAESLKGFFKTILFLIVWVISNLTGMTILVGLIKQEPIYIFPCWHPFDMKNIVFQILILLWQQYVMFTMIFMAFGGGSILFIPYTYIKSEVALLKYALEEIDSRAYEMARNSKFYNDDLDKSRVLSSCYMKCVKMCVEHHLEILRYFSNGKRMVGLSYSTAFCAGIIACTFGGYNINSENLELKFKNLAILFFYTRLSLCNVLDSRRNYK

>HhalOR129

MDPPRFTDYYRWMLQSFIYWGMPTPWLPKLTKSAWWLLVVYDFLTLLLICFALCIFVFTIACGNLGFQDVTVLLPGFVFYTSALYLSLYQYLIKGHLEKIAADVDAIAREIIESKLGLEKELLQVYSDNSKNIIYYCRLLPCLYVSSSTIFFCSVPIVDWFEGNYRTNFAISIVTPFDYRQPGIYEFVVLLMTTALFISTCKQLNNALFFLAFFNTLRSYLKYLYLSMDELQNNIIEEDYTLTRQSIRTWIKIHQEINRCLQVLLQLFSPIIIVNCMYILVYLVGALFLQTQEKKKQHLPDIFSIHWCYGNGYSSVYDLQYS

>HhalOR130

MKVIAPTTIPVKSWRKKREEVVEKKAKTFKRNFDLIFYLTCYTFINLTLYGLIEGILRGEDVYLLFPCWLPFNLDNTFLYIVVILWQAILTANMQFMAFGGLGILYIPYEHIKAEIAILKFALQKMQERSKEMAEERGDFRKDKDKKVDQLLLYSSHLSCMRSCAEHHVEIMQYYNAVTGILTIIYSIAFGTGILCCTFSGYLIVSDNNDLKVKFTGLCCVILLHLFVICWVAEATTNELLSLGNTIYSLEWYHLPKECQSTLRLMLMKASQPLYYYLIFGQRVDIEAYMSLVKASYYYINMMTAK

>HhalOR131

MLISILAKSSLFLKMQATVKSGFYDYEGNMSEEQIIARAVANRDIQVSTSIFSACCMAALLFSYFKAPLFMRTHSLPFPIWLPYKIKSFWSYLPSMIWMWFIAEAMVTCANSNWVALVTLAGHLIGQFKILIMAIKAIDSLAKDKDAEHKVLERIKCCVQHHLLLINFFFDLQSYFNISLLVAAFSTGLILCTLGYLVISPETSLAVAGSLMFVLIPEMCIAGYYCVLGQKIADVTEEIGIAIYNLEWYTMPLPVQRDLLMMLVGSKKTLQLTGFGLHEFSIKGLSEILQASFTYFNMLMALSGGK

>HhalOR132

GTTMTYQDVIEDSDIIDGLSVRYLKLFGLWKVINDYRTTGKRNRIIKITVLVSFILIVPYITFQCISYFKIKVDIQKATFLNLNSMFALQLCCRLLVFWYRMGSQCRLVNLLKKDFLNIPTQNRKAVDEMYKKISKTSNICCTTYMISNIIIVVLLIGKPDTSVDYILYHTGNMDAITTGRKKILGGWYPFPMNESPYYELIFLYESVCVLWAGILIAVYFCLFFQVLCCLYAQFVVLGYDAQTFNFDLENGEMLHHYHLRLSDTFRRILQDHQNLLRYSDELRSVYNPLVTLTLG

>HhalOR133

GTITLSVYIYLVLNQKKLKKFLVQLKVLQRMRREEWEDEMFHKETDKFWRLYFLYSDFIAFYCIVYMTVSFLMDFIVGYFNPDAPSSRLPSAGQGFIDSTEPRSLNNFLASSISILYIAAIYPPFIATEGFLVFSMIYTRTEQKLLNKKVSILKYALENGELDVNKKIKEIINQHQIFLKVLKSLKETIGFPITIKYATTSVILCLNLFTVSTALTTSEYSGTIIAVTAIGSLGTLLLILCTAGESLEAENSELQFNLYDLPWDQFKPQDRKTLVMVLRQVGKPCCINYQGI

>HhalOR134

QDTIRDSDVIDGLNIRFLKLFGMWNAINEYRSTGKRPPVVKIHVFGTLIITIPYIVCQLQSFFNIDFDLQKLTYLYLHPLPAASLCCRILIFWFRMESISRLYNLMRNDFFNIPQHAKAGVKKIFKKISRLSNICCIFLLLWNAGIEILYLIFPGTSVDYIQHHTGSMAKVKTGRKKIFGGWYPVPMSEYPYYEIIFAYEFVCLFWAATLLAFYFCLFFQVLMCLYAQFLVLGFRLSSLKVDNDDSDSRKNNTYNKNNNTRIYEEL

>HhalOR135

SSEYIANHVGNMKDVTTGPKKILGGWYPVPFTRSPWEEVVYVYEFLLFFWVGYTVMIYELVITMEVMTLHAQVSVLNYHVSTLSKKEIVQYSGKKGLTQREVEDLFYKELLAIIRDQEMLFGYGERLKNCFNGYITMLLATGGLLLIASIFQFLFGAKDAIVSINYMFYLLYEVAEFIFLCFATTMLETSSTNIAFSIYNSEWFTSDKRSRDTIQMIMIRSRKPMSLIAVKMYPVNVETLMSVFQFAYSASALISRMVE

>HhalOR136

VLSHHLTTLKFKLNQKTNQRNNSLLYNQLRDIIKDHQKLLSYANELRSVYNPLVTMILGMGIFVLVIAVFQFLFGKTGNPMFIFKFFQFLAYQAIEVSMFCFGSSYIETASSDLQFAIYSSDWYKADVKFRKAAQMMMVRSRKGETLTAIRMYPVNMETIMSILHFTYSVATLMSRMTE

>HhalOR137

LEERALEMASDYGGFDTRNAEVMKLCYKRCLCMCVDHHAEIIRDLVDLWPPELAFLVQEPKRRKKADNYAVKIKFTLISLAQLFFLYIISLIAEMIVEKSMTVREKTFDINWYDLPMECQTILVVMRTVSNKPLETKLLSGQTVDLAGFMSLVKVSYSYVNMLLAITH

>HhalIR41c.1

MSIYHFYFYKTLRSEEIYKQMKLVLLLAPLIFHPSGGIELTEDPTSKSLSSLISKIFLDMPKCVVVVLSDFSSLELPNNFVYLRAQSNSSNLFSSLRASYKMKCKGYLIDENSANDFLTTMGKARSAAPERHTPRIVVIPNHKGNYSVDIFMHPESTYIPDMIVIQIIDDDKSEKGVELEVITNSFHNNSIFPEGEVLGIWPIDDMIFFPDKLKNLNGKELITATLHYPPYVLLHPIFDGMETRMVNEFCRLYNCKPIPLTDEYLWGEIFSNGTGIGVSGNVYMDRADIGIGALYLWENEYQYVDFAYPYLNTKITLLVPKPAQKAEWRIPFLPFSLTLWVLQVLSIVLAAAVMFVVNKVSSHIAQGLMLGGEFATGGGIFLRALAMSLLQPPPNRLPSGSPLRRFFTIFEVLFLFFTTIYSGALSSVLTVPLYYPPIDTPQQLYEAGIPWAADHYAWVFSIQGATEQPFKALTERFEAHSHDTLISLLKKGGYGFGIEIMPAGHVTQTKYLNGEAILNHHVMKHELYTTSLVMNLRKGSAYTEKFNNLIGKLLDAGLLLLWETDVTLTYLSSRLQTALKISEGTKVEVHHPTKLKLTHIQGAFMFLVFGLFVSFAVFMLEILGHSPSSPLKVYVLQKNP

>HhalIR92a

SSLCLEKCLSSSIIAINRTSVNSYLEVLANEIGEKYFSGHRCVVVMSDAGLLRNFKINTTVVHIEMNPTNESCVQEVADLLRITYRDNCDGYIIQSSNPNCAFDGWLDIYFHSSVARHNPKYLFLPVYPHLLTYGDELLSRRESDQTADFLVSEINENSTDWAVIISTNNFYVHPTVPGREPKIYLDEWHYETGFKENVNLYPDKVLNLKGKPFRIATDKYFPLTSAYPLEGSEVRISLYFCEKYNCTPEAVTDNSLWGTIYDNLSADGVLGNVYNDRADVGVLAVYLWLNEWYYIDYCTGYLAADVTVILPKPTKLSPWILPFLPFKYDVWIAFFISLFLSASSLYFITRVSIRFTRFRDQLIMKVQFTTIDDSLMRAIGLAVLQQPSSRLIGDSPNRYLFTSYEFLYLVLSAMYSAELASFLTVPLYYPPIDTFHAFAHSGIHWYATGSAWSNALKNSDEEDAKLIVNRFGVLSMDQLKEKIKEGKHGFTVERMAGGSIGEQDFQTSDVIPMYHIMKEKLFGSPLVGAIKKGSPYLKHFNEVVLKLVDYGHYLYWENDVARQYLGSSIQAAYEEAKTVQIDDSPAVIQISNIQGVVLIYTIGIIISIIVFFYEIITYRKNENSLLIEKKK

>HhalIR75b.2

PLKVNLYLCIYYIKVFWSSMVNYAFYFDIVNKYFNFLHIQSVVMLICPQEAVKVNVMKEMSDRSFLLSFSVDSIRDNPIIRTGTVLDISCHNTSLILQMMSARKMFNSDMEWLLMEEVNTTESFQQTLENDVLKDTLALPGSCVTLAQFSENERRVKFYEVYRTAIWEPMKFRFLTDYFMNGSKEFSFRRGYRSFEGVVLRTASAVLYPGLFLGWESQELKEVDTISKAGYAFMKDIADHLQYNYTLLFLDFYGYETNGSFNGIMGYMQRGDIDVAANGLMMNQERMPYVDFVGDILVLRSPLIFRQPSLSTVSNLFVLPFHKTVWMLTVVVTLLYSMALFLNLYLKMRLLRLKETDDSSVPEIISVIMAYVCGQGTGLELRPGAGRITLCILSVFCFFLSVSFSAKIVALLQSSATTIKSLSDLTHSPMSVGMQNVFYNTHFFSISTNIEVRELFQKKILPLGDKAIMKPDIGIQKVKEGLYAYKVETPWAYTIITRTFEDKEKCDLDELNPFPLPTLAVGLQEKSGYKEPIARSFSRLLEVGIKRRTMNIYYPQKPFCNSNNIGYTRVKLTDFKPALDFILYGLLSSFILFLFELLFKTRYLFRRIVRKEWPAHKEVI

>HhalIR1

MKVLLFYFSLFFVFFCTKGNIELNFNFNNDYLFPIDSTYLIEMMNYTFRDNYCVQLVADESIIANRIREAMFNVNFLRGVSDKRGRFQCINCVFIASTVEIFINILKTRPVDTDKFLSIIININDENVLYELSHKDVTNYLTNWHGVVIDLKAPHFLYRYIPLHKTFKTHNLSDIRNREIGINYGSFGGRKLRVGTFNCSVNSQIGPLDAKGRPSWFGGVEMIFLDAITQKLNFTYEIILPPDGEGVGGRLDKYGNLSKGLVGLVLDQSVDVAFCGIWQTNYINTRSLSISSPIQEVCITYLVPRPLPFNQMGLGLFTSFEGKVWMLIFISIITTAISSQLIAFMAVKHGFSNFQLIKFLKPNNSLLQLWSILCNNAPKSLATFGPLRHVLLWWAVYSLLLNAMFSSSLVSHLTATLYDKPLDSIQALVENDYYWSLNGMPYLGSILNSSNEWGKKWVKRFHFKSEKSLCSILQVDNNKLAMFAVNYDYGVMLEENIECKHLLRKYQTLPDCISKYRTVFLLRHRSPYRELFSDYITRYRENGMFQKQYRELFYKKILKDNYISEVRLPLNINSAKKVKLRLSNTLAIFYIYGLGIMISFVVFLLELLWYRKSYMLRIFV

>HhalIR41c.2

MIRLKNMFHAIMVYLLLHCSYGLIKQEDTRSQKLNKLISQIILEMPDCLVIVMSDSNDLKVPKDIIQMRFSPSSDELPAFLKEAYELKCDGYLVDQESAGYFFISLKIARLHANVRLVPKIAIIPNKIGEYSEALFRDKETAFIPDLTIIQIDNSTTEERFMVVTNDFQGTNRKLKGDFIGIWPDDKIVFFPDKMADLKGKELYSSVVHYPPYVLAQDPVEGVECRPQLEFCRLHNCSLKIRTSEYLWGDIFPNGSGNGIFGEVFLDQSDFGVGGIYTWLEEFQYLDYSTPYGIGRINVLVPKPTKVDEWKTVFMPFSPTLWLLLVFSILVAATLMHCANIAAGKISDELILGGEFTTWVGIFFRAIGMSLLQPPPSILSASPLRRFFTIFEVLFLMFTTIYAGAIASVLTIPKYNPAIDSALQLYESGLRWVALHEVWILSLVESTEPYIKTLVGRFETQDEETLVRYTKEGVYAIGIELMETGQMIEASYLSVETVANHHIMKNDLSWSHVIMLHRKGSPYLEEMNRVIGRCRAAGLFLLWATNITANHLSSRFQIVMQISKGSSKVEIDEPIKLKLLHVRGAFMLFIAGMSLSSLVLMVEIIKFKIFNTKSRKLI

>HhalIR84a

MKLLRMIFFIFCYEGTWSSSRCYIPDGIHDVIDFYFRDLFGAHLYFCKLEDAVKAFKRFTSGGPKYNIRHNRDFREPHELLMSYWQPSRLGIFLDTSCDHGIFFFNYTTDLFNASYSWIVWSEEVNFTMFEDTRLSADSEVKVVKPGLEIYDIHRVHISSELKSKLVASWNATDGLVQISPSMDRRDFGGFTFTASIMMIDIKFNESNLLEPLLDKTYEPGKDMVRRYGLAVFLHVAELYNFTYNYILTNAWGDPQPDGTWTGMVGQVARGEAEFGLAPAKYIVQRFEIIDFINSMHIVKCCFTFLQPKLFGSAKALVLPLDEIVWVCLAVLGILSVIVFRILAKYDNTGLSNDSWGGSALLVVGAISQQGIPDNTEKVSTRIVYIFLLIVSFFVAVYYNTAILNGLLLPAPNAIQDIEQLLKSDIKLGMLDIPYLRNELIQNDTMTIRVREKIAKAKPKQIFYSVPDGVRMIKKGKFALFTEDEAIYTEILHQMTDGEVCSVSEVLKYNPFHVGAVAKKNSPYKELFNRAFAVMRERGILKRQLEHWLVKKPECNWKQDALSLSMEPLALAYALFSFGAIFSFFILGFEIIHSKKSKKKAFD

>HhalIR75h.1

MRLQVLLIFALFSTCTCKNMTDYFEIINIYFSYRNVKSIDIFPCTESEGYWMFKRLLKDGYSTRVNRNLLDKEFQELPYIKGWFVDLTCSKNIIFFSNNVVQITGLDGIWLGYPSVASDLNMTSLRLDSDVMVGSADGRLWDLYGKPLERMILNGAGTWRPANISIPKIKFRYDLGGVVLKGVLVELDLQFFANNITHKIADHDYFPDMEMTQRVSYIFVTYLAKFYNFWFDIVPTDTWGANLKNGSCTGMIGILQRKEADIGISACSFRVERVEVVSFAQRSQELRFICMFMEERIHGTYSSLLIPFSFQAWLCVFVAIVVGATIFSFLQKRDLTEGIIFVTAILSQQGLQKDYRSISARVYSISLLILGLVLYSYYSAAIMNGLLSPAPGSIRNVDDVVKSPMKASLARVPYMIPKVNQKAYVTPELLAKTKRQEKSQQILDVFVGVDRIRHERLTLVADDMSLYAIINEMYTDAQKCNLMEIEVIRSFPFGNPLQKNSQLREMMSQGTLRLRENGIAKREMRVWYHPKPQCLGSTTYTHVTLEAVGLAFTLFMTGVVLSIFILLTEIALKIFLKYNLFSKKFSKTQARPFLR

>HhalIR75h.2

ESMYRLVILLLIGSTHSLRYMNNTLEIITTYFNFRATNVINIYSCNHREGVWLLKHLNALGFMIQIKHHTFDEKEESRPPYVRGWFIDLNCTKNMNFFKTINGLDGIWLGYPVKSLDMNLTKIRLDSDVVLGTEDGLLWDLYNNTKRNLKVSSAGTWRPPKIFMPNSKHRYDLNGTVLKGVLLELELQFFKNNMTEKIGDYSYYPEWETSERLAYVYVQNLAQFYNFEIEIVPSETWGYVLPNGSFGGMVGVIERGYADLGLTACAMREDRMRAVNFVPQLQDLRFICLFMEERIRGTYSALILPFSFNVWLCIFVAIVIGAAIFSLVQNKDVTEGIIFVTAILSQQGLLHDKNRMSARVYSISLLFLGLVLYSYYSAAIMNGLLSPVPPSIHNELELQKSTMKASIAKVPYLMATFSQKAYLTSGMFAKTREHEDTMMDPFIGLDRVRKERFILISDDLALYGILKVAYTDLEKCNVREIETMRPFPLANPIRKDSPFREMIAQGMLRLQENGMGKRDRRVWYPPRPHCFASTIYTHVTLEAVGLAFTLYVLGIVLSFTILLSEVVMKRFKKFKRTDPDTEFQGYY

>HhalIR8a

ELLWDLLRQMSMIWRAIFAIGTALFLASDVGSVKLLILKEENGTIWENVKTEEVWEEQHVILNRENEEESFNKVCEELSSGAWMVLDLTWSGWDSVAEIAGIRYLRADLGISPFMRATELTLIKLRNSTDAALIFQHSHHFEQSLWYLVRESSLRVTVHLGLDDESAESLLDMRPSPSSYVIFADHDKANTILKKAVDKKLVYLDDRWAMVFMDIGTAPINKTMLKKRIMQVYMPKSICCTDPGQMLPCHCTEPFNFEIEGAKQLKIALSKAISSSLTEGLSAEPLNYSCSSSALSPKNDSIFYDNLDKALSDSWLLHKTYGEVRLNLRFEIRNIGENRAQQLGNWDPIAGIRTAAMPRVKRFFRIGTGFSTPFAYPTNQISPDGSARWEGYALDLIERLAESMKFEYQLITVENHMFGQRFENGSWNGLVGMLATGRVDMVVGSLTMTSEREEVIDFVAPYFEQTGFSIVLRKPLKKTSLFKFMTVLRVEVWLSILGALCLTALMIWFLDRYSPYSARNNKEKYPYPCRDFTLKESFWFAVTSFTPQGGGEAPKSLSARTLVAAYWLFVVLMLATFTANLAAFLTVERMQSPVQSLKHLARQSRINYTVVKDSDAHNYFRNMKFAEETLYKYWKEITLNSSSDQSQFRVWDYPIKEQYGHILNAIEKAGPVANIQEGLRKVIESEQAEFALIHDLLELKYHVFKNCNLTLIGEPFAEQPYAVAVQQGSHLNEEISRRILDLQRERFFEATASKYWNSTIKSKCDNVDEDEGITLESLGGVFIATLVGLFIALLTLGAEVWYHKKKSKNQVTMKGQQQLQSIIRDEIFTTKEFGHHNFANRKASLLATKPKVKQITVYPRGQLY

>HhalIR75d.2

SQCNTMLGQIHTIIDYFKALKISSLSLLLCLSEEMKILALRQFSQEGFQISFGFSSDSRGTVLDLNCNNQTGGRMDGEWLLLGKAEDATELRALPDSKVTLLTEEGVLMDIYRPTMSSLLSFANYTGQRDHSDLKGETLKAVTVEDEGTSVRLEEGGDLFGHYGCLCKNAGGWSPQMSEVLVENLKQQLNFKVKRKKVTDCNALYSTLSEGAVDFSSTAIMILPEKMEYSSFTGDLFEYKSMILFKHPRLPVMKNIYVLPFHRYVWFCCVGLFFLCFGLLLAAARSYPGVFEEYTYSSPSDILTLLIGVASQQGIDLGARSPSTRLALLLFSVCSLFLSTSYSANIVALIQSGAETITTIADLVQSPLSFAVQDVSFVKVYLKNGEKEVKDLYETKIMKGNSNPFISAERGVEKMKNEVFAFHVETSIAYNIMSKTYTSQEKCGLSELHVYTYPRFSIPVIKDSGYRDLFASRLSRQRECGLIKRAELLALERKPACTAKDSGFVSVTFGDFLPGLLVLCWGMVAAVIALFAEITMSKCTLLAKQEKKKEK

>HhalIR76b.1

DIAEGRLPAMQGLYYLLLALCSNYPPPPSEAEFTCKLRKGHPEKEILKGRTLKIVTYDDRPFSGATANASGALEGHGLVFEVLETLQEKFGFEYELQKEKRLMGDETSGLLGMLVAKDVDMIAAFLPILPGTHNYVTWGTQLWQAHYYVLMKRPDDSATGSGLLAPFDDKVWILILISLTSVGPIIYLIMWLRIKLCPNDNKQLFPLSSCIWFVYGALMKQGSTLSPLSDSARLLFATWWIFILILTAFYTANLTAFLTLSLFTLPIKEVEDVAKPPHKWFTTEGSSVEYAIKNKDDGDLNVLLSSVRRGNGRFIDTSSENHVLEMLYDGWLYLDTSDTLNRLMFDDYKRKTIEGEDENKRCSFALTQYPFLVRSLAFAYPKGSRLPELFNPIVQVFVESGILKHLLNEDLPDTTICPLNLGNKERKLRNTDLFTTYIVVLAGFSGALIVFCIELLWTYCATRSFNSKSNKLQRLKNNYNKFVIPSEFEKNAAIPNQVQTKINGREYFMITAKEGDKRLIPLRTPSALLFQYGLNYPTMF

>HhalIR75d.1

MGVHTEWLLLDDGSSLDVIKDAYILPGSFVTIAQILGEEVVYLDVYRTSPYRPLKYTVLDNQTLEQFIQLPDRPSRNDFEGISLLGAAVLYYPHMFCGFDCRDHPEVDTIAKTGFPISQHFQEQLNFTLTFQILNDYGWKTNNTFSGLMGLLQREEIDMGVIGIFMRPDRIGTVDFTGDTFEIKSLVIYKQPALSAVSNIFVLPFTRMVWVCCVALSVLTGIFLMADVATSFGNRQTFEESVTTSDVVTLIIGCICQQGTTLVPKTLSARIIIFLFSLCCLFFYTSYSANIVALLQSSSATFRSLSDLTNSPLGIGIQDVIYNKIFFGEATDENVRELYDKKIAPQGPKAYLNPVEGIKKLRSGMFAFDVEIHWGYKIISDTFHENEKCDLDEMRIFLLPKLSIPVVKKSGYREYFTRMNTWQRDVGLHSRIRQRWLPKKPICDNTGRGYVSVGLTDFKPALLVMVYGISFSIAAFFLELFTRSKLFYRCKFLNKSKRKRRKFNDWDRVM

>HhalIR93a

DFLEKKELLESGDWSPRSGPVLVDQIFPNVAHGFRRKIIPLFTFHNPPWQIVKYDQNGKPSQVKGVIFEVVDHLAKSLNFTYEIILMSNTSLPANQTKFYRFNESVGDVVLDQSTEFLAWEQVVRLIQNKKVLIGAAAFTITEKRKKYLNFTLTIRTENYAFLVARPKELSRALLFIQPFTSDTWQCIVAAVLVMTPLLNFVHRVSPFYEHYSQREKGGYMKMMNCFWYLYGALLQQGGGVMPEANSGRLVIGTWWLVVLVLVTTYSGNLVAFLTFPKMDKVISNVDQLMEQRGEVTWGMPEDSTLHIILKSTDNDKLNELSDSAQLHRMVTQDIVSQIRKGEHVYIDRKSILLYLMKQELLTTNRCSLSIGEEEFLAEHLAMVISPSSPYLELINKQIYKMHQVGLIDKWLTDYLPTKDRCWSNTLSSESQTHTVNLDDMQGSFFLLFLGVTLGFILIIGELLFKKWKKTQEKQVIHPFVT

>HhalIR2

KNLAIIGRYNDEKVTLSRLHSFSEDKETITLASWSWKEEFRGTLFPKDEIAPAFKSKQLTVLLRHDPPFVIVKSYLNGSKYADGYLIDLWNCITERLDLSFEIKYFPTMEGGDFWNFANNGLRVEMERSEMDVALYASADPVDIYSNYTGVHTGVHLRLCTYEHKKRINFYQFIKTINSSCFAVLTFIGVLSAFLMYIAGNNLADSFLYFLAISFNQGPGEEPPTTSSVRIVIISFSISVLVFFYVSSASMASLSVNNQDLESTTIEEVVDHSYIRRSIIVEYTSAHVMMRYSDLATTFDKLRRVKMAKLPSVRDALTVTSKLKWFSFIERERVWPYWEEFKCCVFESDLRIVRPTHFMVRRNLSYTQVFRQESARLMENGVVSLLIKKWWPIVDASRKEFIPISLADVSIIFDLFAFGALVSLLIFFIERHVGILFH

>HhalIR68a

MFILLIFIILTVDCSLVSCTLSSSLIKRLWEYKKDDDFGWLLEDLIQRVLFDAKCLTIISDQFYNDMFTTRMFQKLSVIPIFVIFINENEDLLSPNYKTLSVILQARRKGCNGYIILIANADETMRFLRFGDRHRVIDTRARFFLLHDVRLFHKDYFYLWKKIVNVIFIRKFFEVNRYELMTVPFPSPIVHNWKPIRIDSWKSGKYQKNNELFIDKTSDLRGEIVHVAVFEYMPSVLKKVVEDDQSGNIMPIEYSGLEVLILRSLSDAMNFQILIYEPPNSKTEAWGKQQLNGSYTGLLGEMVSGRADFALGNFLYTPKNIKLFDLSIPYITQCFTFLTPESTTDNTWKTLILPFKKFMWLGVMLTLVVSSLVFYVLANFHKYYENGRISVRPVSIFVKKFKL

>HhalIR3

WTRVLLLLAVTGGSWGRKRGISDMAKRINEDNKHLNHSDAQIHRLFNYVMPDMRCYQVETDGTAFGQSFQEDLHQSNAVPTLVTGGSWSVVEGCKGFVIIATFFSSVIPIVQKMPRWAEHRILVILKGTGANTLLVPIMVDMRIFRDAEVAIVSSSDLTFGYRLTWTERYYVSFRSEPNATWKRLKEGPNDFLGREIWVQTSSCSMFSQIGPINAKGQPAWTGGAEMIMFQDIAERLKLRPRFNYTKFLHAGWFKEPLTSENKSDVAFCGILVSSRTLELKNIKVSQPLALLCLKLLVPRPQRVSDQWDEIFEPFSPGLWLLIATVTFFTTFLLQRFTTVTRRLVFTKRIKSMAGVTYIGWKIIFSFLRYKSLLLSRIRIVGGKFPSDRCYFGLIRRA

>HhalIR75f

MLDFNYEPEEEFLSRYGFSIHKILEDAYNFRMNISIYDDWGYFNEKLKIWDTGMFHGLSAGDIDLGTSISRVYGQRLDVSLYFPPYLKFRTCFIFKHPSRLGEFTALVKPLTLGSWLCILSGVVLSGLTLWFIKWFETVDIPSNENDLASSLLSSLGTFCQQGLSSDSLRLPVRVLYIFLLVASLVIYMFYGAAVVGFLLLPSPKTIDTVEKLIDSPIIPYAENLAYHKTHFQGNFSDKAAKAYEAIKETKTEKERWIDLTAGIQKVKQGRAALYAQDTNLYRAIENSFSNSDICVLAEIEIVLIWASTVIRKKSPYKELLYQGMILTHENGLLNRVMKTWQAQRPTCFAQNESPTVSFEA

>HhalIR4

NNDYLFPIDSTYLIEMMNYTFRDNYCVQLVADESIIANRIREAMFNVNFLRGVSDKRGRFQCINCVFIASTVEIFINILKTRPVDTDKFLSIIININDENVLYELSHKDVTNYLTNWHGVVIDLKAPHFLYRYIPLHKTFKTHNLSDIRNREIGINYGSFGGRKLRVGTFNCSVNSQIGPLDAKGRPSWFGGVEMIFLDAITQKLNFTYEIILPPDGEGVGGRLDKYGNLSKGLVGLVLDQSVDVAFCGIWQTNYINTRSLSISSPIQEVCITYLVPRPLPFNQMGLGLFTSFEGKVWMLIFISIITTAISSQLIAFMAVKHGFSNFQLISKYSKLYCIRYRVYYLF

>HhalIR75b.1

DGVTFTAATSVLYPNIFEGFSEENLNHPEADAYAKVGFAIERNVGQQFNFSFTIKIFFNSYGYLKNGSFTHVMGLMVKEQIDFTTGLMMRDERMDYIDFAGNTFATYSPLIFKQPSLSSVSNIFLLPFEAQVWLATGVLLFVSTIILFVEIIITSRLLFRTRYSFLEVFMGILEDAFLQGSTLQFESAAAKLTSLLFSIVSYFLFIAYSAKIVALLQLSTSSITSLSQLSNSHMAIAIQDVVYNRVYFQETKDPYVKEFYQKKIYPLGEKAYLPPKDGIMKIRSGFYAYKLETDWAYKLISETFNENEKCGLTEMNIFVLPMISPAFPKRSGLREHFSRSIIWQ

>HhalIR21a.1

TYWMFTIIITACYTGSIIAFITLPVYPEVMNTMHQLLHKNYRVTTMADEGWWTLLIGSDDDVASGLAGTAETVNNVLEGLSTVIKSSKEDKPVTFLGSSEHLKHILKSNYSASDVSKRQLFHISRQCFVPQMISMIMPHDSIYIDSFSRSLIKAIEAGFINKIQQDLEWNLYKSSARQTLLQGNLKLEALERQLTLEDTQGMFLLLGCGFAFAVCAFGFELGTWIKNRKGEKLTIVVKERILAASRRVSAAFLTPYKSHIRDHYPLYFRNVSRRKSSLFGSMIILEASDRQKPPTAVSEIHLMPPKVEKPIRLLSF

>HhalIR21a.2

AFKRILENGEVIWDGVEVRLLLLMKEILNFTMEFQDHIGKQSSDTLQILRQGNTDLVVGGFVMTKEIYGKTSMIYPHFMDCAAFISLTSIALPKYKAVMGPFLWDVWISITLCYILAIIPIAFSAWHTLGPLIQHPSEIENMFWYVFGTFTNCFTFRGQFSWTKSVKNSTKLFIGTYWMFTI

>HhalIR60f

LSTDLRLGLVWITAAGDKNLVFTFPSLIIPHPFMTEHICFFFKNPKEVATWKLIFVGFNDVVWIVLIATAFAFPCCLFLLARFQNYQHPFQKFSISIMSSYALLVSFPSSVDPRTIVFRLAFATWFFYTIHINLAYSAALKSLLTAGKTEPKMASF

>HhalIR5

EWYGIFSPYTTNLWLAIIVAYLSVSLLLPTLAYMDSLISVFGECRYLSFERSWILLAGMLLQGNWMRSTHSQGPCRHLIAWWTVFSLLVGSVFSSSLASYLTRAGYTWKPETIEDLLQTDYSWT

>HhalIR25a

MCRLLLLLLLEHALSQTLQTINIMFINEAKNQLAEMSFDVVLNYLKKNPKLGVKVEAAVRVSISGTDAKAILESICEAYNGTVSDGKPPHLVLDSTMNNVPSEAVKTFTDALALPTISSSFGQEGDLRQWKTLDNEKQKYLIQINPPADIIPEIVKSIVQLQNITNAGILYDDSFEMEYKYKSLLINMATRHIIVHVHNADSIENQLMRFRNLDIVNFFILGGLSTIKMALDTASKKQYFGKKFAWHVITQDKGQLSCSCSNATILYVKPEPEPGMKERLDGLRNSFNLVEEPEITSAFYFDFFLHSILAIKNLLDEDDWPKDFNYTLCDDYRADREIVRKEIDLMKNLRLVSEPYSYAPFLLERNGNSFPEFVMKLEKVTIVNSQSESAESMGTWKAGLISPIILKDATAMNNFSAVTVYRVVTVKQKPFVIEYEEDGRKKYKGYCIDLIDEIKTLVGFDYEIYVAPDNQFGNMDENGNWNGMIKELVDKRAEIALGSLAVMAERENVIDFTVPYYDLVGITILMKKQTTATSLFKFLTVLENDVWLCILAAYFFTSFLMWVFDRWSPYSYQNNREKYKDDEEKREFNLKECLWFCMTSLTPQGGGEAPKNLSGRLVAATWWLFGFIIIASYTANLAAFLTVSRLDQPIESLDDLAKQYKIQYAPLNGSVAMTYFQRMANIEKRFYEIWKDMSLNDSLSEVERAKLAVWDYPVSDKYTKMWQAMKEAKLPNTLEEALDRVRQSKSSSEGFAFLGDATDIRYQQLVNCEFQMVGDEFSRKPYAIAVQQGSPLKDQFNNAILQLLNKRKLEKLKEKWWSENPERQKCEKQDDQSDGISIHNIGGVFIVIFVGIGLACITLGFEYWWYKYKRPADGGGGPMVVKPTIGGGRNVEKLSVTGLADFGHHTTFRSRNTHNSNMRRGNLSHIPTSQW

>HhalGR1

MINNNIMRDRIDVDLLGNITTQSILHYLSEEERKDNKKKKEKADSRSTIQLELRTPLLLAQFLSLLPIYGVSNPDYRKLRFEWKSWKVLYSFGIITFNIILCIFALNRYIRDRGVTYRSLGNPASIRDIMFFGSTLLISMSFLILVRQWKDFIGKWASLQGEMNNFESSNVQKKIKLLTNCLLFSSIFEHILSNTQFMLNVEENQDRLRMFLDDKNVSSVLGFNTWCYIFIYFGNFLITLIWNYTDIFLTAVSLSLANMFKQFNRDLRMTAAMVSHDNALSYWRRKRELYNSLSLLAKTVEEKISRLVLISFSVNLYFICIQLLNSIQPLTSFLQVAYFCGSFGHILFRTCSVCFAATSIYEHSKGSLPTLYSVSSEQFNSEVQRLIDQVTGETLALTGCKFFTVTRTLMLTIAGTIVTYEVVLVQFNNPTDDSGSDAHNSSRRA

>HhalGR2

MINNNIMRDRIDVDLLGNITTQSILHYLSEEERKDNKKKKEKADSRSTIQLELRTPLLLAQFLSLLPIYGVSNPDYRKLRFEWKSWKVLYSFGIITFNIILCIFALNRYIRDRGVTYRSLGDIMFFGSTLLISMSFLILVRQWKDFIGKWASLQGEMNNFESSNVQKKIKLLTNCLLFSSIFEHILSNTQFMLNVEENQDRLRMFLDDKNVSSVLGFNTWCYIFIYFGNFLITLIWNYTDIFLTAVSLSLANMFKQFNRDLRMTAAMVSHDNALSYWRRKRELYNSLSLLAKTVEEKISRLVLISFSVNLYFICIQLLNSIQPLTSFLQVAYFCGSFGHILFRTCSVCFAATSIYEHSKGSLPTLYSVSSEQFNSEVQRLIDQVTGETLALTGCKFFTVTRTLMLTIAGTIVTYEVVLVQFNNPTDDSGSDAHNSSRRA

>HhalGR3

MLTIYKNQIFAEGVYSRNHHKNVTNVGTKHRNIHFKIQPQLMAKDNIYFNEMKPIFIMLRMVGRFPYSFTKTGFAPFSFISWPVLYSLVFNLVFVLMTIRSMQIMINDKIYPSRSYDETLFWFLLLLFALQSFTGPITFWMDAPGLVSYFQKWKDFEDFWRLGTLYSVNRHRFTRFFSISIMPLVALFVTYETLTLPKISILIFLPHIPIVIALLLMQVYWWLTLHDLTQYSEKLLTSMLKVKDRRGMGYRRRIWQQISNLVTEIGNAIGASGLSYSITNFVGFILSTYGILINLASPGSSDVSVMGLLLPAVACAMTIFLVTDAAYKATECVGHKFTKNLLQIDLSTLSRSCLSEVDLMFNSLSANSPVIEYLGFMKITRNTFLQFVSHTATYLIVLVQMKTQPKHSKNG

>HhalGR4

FNVLWPNKTLNMMKPDVKRCLGILMGPAKMLGLFPIAWDERRCYRISVPFIAISSLKCLAYTVVTIVYLSVNFTASENIALGAEIDYISLILINAIPILSVCELVCTLHEFNECIFFLEAAELQLLQLGKFVDYDTSKRPLWINALLALGAFFARFAKNAIIDPAAVLLSGLQVLVTFSLISHMIVLVYWYFGVVGILTKLFAACNQEVRNYVRDFVVLKMRKVEKLARAHHTLCLCTTTLNDIHGAQLVAIFLSCFVLSVTEVYRCIIFLEEKVDVTFFLVIAVKLCCIILCFNLCLQIVTACKECSAEAKEFHTLLYQLMLDDKTNDLSNNKKLCLHIAMKREVVFTACGFFKLDYTLVHSMIAAATTYLVILIQFGQPRSMPTLPTDSLKSSEYTNATSPSTRII

>HhalGR5

MKQGSMSKAWITTSVSHNKPYKKVPVKKYFQEIKPLILLQRAFGKLPYSFNEEGFAPFKLLSFPVIYTIIFIVFQSTWTVYSLCIIIQEKIHKAPSYDVTLYWVSIGLFLLLNFTTPMTKWIDIRKFVHHVSSWQDFQNNHLDAELGANLSLTLMIASVLLLPIASVFIYCQSYLLTDLSLFVMVPYIFSFVETGVIIIHWGVVLYELRIASRTLLSKIIMDGCRQMSTYRRTWLELSKLVSGVGESLGHTGLVISIVLFTTFVLAMYALLSSLFEPAKTCNHVWGLLINAVLSLLCNLFLFNAAHRTTQEVGPDFSCKILASDLTHLSQVEMNEISLMVQTISANPPTVEYLGFVTVNRSLFVSLVSNAVTYLVVLIQFKASAPEKPVKEEVVQ

>HhalGR6

MYFSSECIFNIILLMTKLLGVFPYDHVRKRVSYQWFTYSLIIVLVCTVEAAFVVFDPPRLAKNSLLRNILFRCQMLAMYACIVIFNACILIHRRKISLVFLILEKLKSETRGQPSFLLVLGYFCWQSLAITVTAYNDWNGNTPLDGTVSLALCYYYGMISIIVVGTAPFVALVLLVSTKLEHLVIALKSKVTLGSGRLYSYVLLYDRMVSLLTSLNEAFSLQLLLISFVSFFNLTINMFFIADYVVNDASIHRAKMVPVYMGWITMFSSMVIFPVYSCHRTTKQAKEFNTQLYQLMIDDTTNDISNNKKLRLHIAMKREVVFTACGFFTLDYTLVHSMIAAATTYLVILIQFGQHQSTAPELPANPPDFSNTTALPLSTSTI

>HhalGR7

TYTSLQDSTMVKGVHNALSLVLALSRAFGAFVLRAQGRTYVFSYKLISYFIVLAMGTIAEGIIEASNLQSECSFSLLLIVDIFVFCFYLITAIIVLYQHYRLREPLPAIISELEDMEDHIGDVSYGRYYSYGVIFLSILNALPRIFAVLRRSFAWNYLLTRILYYSFTQIPLLIAAQYAMFLHILSRQLYTLSEQLNTAIFNLEVWRLIDIHHNLVLLADRINRAFDLFLIHMVTFIFVINILRLYFLIVYIVNPVSYTDLELTIISFVDILINCGSLIIIVSAAMEATKKAELFNKQLLKSLLISKTIAQDEKIRTYLGMKHSIQQSACNFFSLDYHLLTSMAAGTTTYVILLVQFTLL

>HhalGR8

KGVHNALSVVLALSKAFGAFTMSFQGQIYVFSYKLYAYFIVLVIGVLVQGIIDAFIASSEHPLSLVMFIDYFVFSIYIFTAIIISFQHYYLRESLPAIISELGDMDQLIGNVSYSGYYKFGVIVLFVLNALPRIFAVAERPFTWNHLLKRTLYFFITVVPVLVGGQYGSVLQILSRQLHSLSEQLNNFQSNLEVWTLIDVHHNLVLLADRINKAFNMYLLNTITFCFVIDILKLYFVIVYIVKPVTYTDLELTVISSMDILVNWGSIFTIVFAAMEAKKKAELFNKQLLKSLLISKTIAQDEKIRTYLGMKHSIQQSACNFFSLDYHLLTSMAAGTTTYVILLVQFTLL

>HhalGR9

QNALSFVLAMSRPFGASVLRVQGWTYVFCYKLYSYLILLVTTTIAEGIIETSFLISLYSLSFLFIIESSVFWINTTTAVIVIYQHYQLREELPAIISDLECMDSLIGGVTYSGYYNYGAIILSIVTATPRIIAILLRPLSWNNVFMRVLYLFITEIPILIATQYAILLHILSRQLHTLSEQLDAVMFNIKVLSLIDVHQNLVLLAGRINTAFDTFLMYMITSIFVINISTLYFLIIYIMKPVSAIDLPLSIVCALSFLVNCGLLMLMVFPAMEATKNAELFNKQLLKSLLISKTIAQDEKIRTYLGMKHSIQQSACNFFSLDYHLLTSMAAGTTTYVILLVQFTLL

>HhalGR10

GNFIRRMKQGSMSKAWITTSVSHNKPYKKVPVKKYFQEIKPLILLQRAFGKLPYSFNEEGFAPFKLLSFPVIYTIIFIVFQSTWTVYSLCIIIQEKIHKAPSYDVTLYWVSIGLFLLLNFTTPMTKWIDIRKFVHHVSSWQDFQDGCRQMSTYRRTWLELSKLVSGVGESLGHTGLVISIVLFTTFVLAMYALLSSLFEPAKTCNHVWGLLINAVLSLLCNLFLFNAAHRTTQEVGPDFSCKILASDLTHLSQVEMNEISLMVQTISANPPTVEYLGFVTVNRSLFVSLVSNAVTYLVVLIQFKASAPEKPVKEEVVQ

>HhalGR11

GNLLRRMRNRTLSEAWVTTSVFSIKPYTKVSVNKYFLEIKPLIILQRALGKLPYSFNKHGFDPFKIISFPVLYTIVFFTLQSAWTIHTMSVIIKEKIFNAPSYDMALFWVSLELILLLNIASPITKWIDVHKYVQFVNNWKDFQNGCRQMSTHRKTWLKLSKLVSEVGDSNAHTGIIMSVTYFTSLVVTTYALLSSFSRLADYNSHFWGHLVSTLIGFLSNFVLCDAAHRTTQELGPEFSSKILAMDMTHLSQSEVNEICLLLQTMSAHPPLIGYLGFVTINRNLFVSFMSNAVTYLVVLVQFKSTSPLNPIKEDITQ

>HhalGR12

PFSFISWPVLYSLVFNLVFVLMTIRSMQIMINDKIYPSRSYDETLFWFLLLLFALQSFTGPITFWMDAPGLVSYFQKWKDFEVKDRRGMGYRRRIWQQISNLVTEIGNAIGASGLSYSITNFVGFILSTYGILINLASPGSSDVSVMGLLLPAVACAMTIFLVTDAAYKATECVGHKFTKNLLQIDLSTLSRSCLSEVDLMFNSLSANSPVIEYLGFMKITRNTFLQFVSHTATYLIVLVQMKTQPKHSKNGPFPDSYGNSTDFENT

>HhalGR13

EVAILKKLWLTLANLTTELGHVLSLTLILFMISCSIIGIANCYSLLFFLRDCFLGNCDSSSYQQIPIISQVGTLIVSAIIIIAICEHGHRCTVSVGSNFLKEILKINFSLRNENTQRELHSLVQTILLRYPDMALGCYFTVNRRLLATMVTTAITYLIVLLQF

>HhalGR14

INRAFDTFLIHVITFNFVIGTMKLYFVVVYIVKPIAYTDLELTVISAVDFLVNFGSIVIIVTAAMKANKKAELFNKQLLKSLLISKTIAQDEKIRTYLGMKHSIQQSACNFFSLDYHLLTSMAAGTTTYVILLVQFTLL

>HhalGR15

VELWSVVDTCEAVIQKAKDFSAALYQFMIDESTESCKNKKIRLHLTVQKEPCFTAYGFFDLDFTLLHSIIAAATTYLVILIQFSQTTTSYPKRVLLNTTASYNSSYSNYTE

>HhalOBP1

LIILASVLALAATAKTQAKQQICVAPTTAPHKIEKVLSQCQDEIKYALLQEALSVLGQSVERQKRETFSGEERRIAGCLLQCVYRKMKAVDDNGFPTAPALVQLFTEGVKDRNYYLATIQGVQQCLAKEIQQRKSNQSLAEAEGYTCDVAYDMFMCVSEQIESLCGISP

>HhalOBP2

MKTFTSLSLFMTLFIVAKCDITSEPTTTPQPSGGSTPTGGVTVSKSPEEIRHKIKEQVLALTESCKTSTKITAEQAKIVSNQAIPKTEAEKCFLECMYNGLNITKDGKFIEISAKGLAQHRFANSPDELTKANNMIETCTKEAIVKDANEKCAIGRLVRECFVKHGSKINFFPKP

>HhalOBP3

MTKYISVVPVTMSRLHDVKLMLLCTACLMAVAHGGVVVDECTSFQEKPPPLCCRYGDGNNDNDKEEEMERNMKLCLNEYMGDLSRTSRGEERISLMECVGECVFNYSKLLTPDLKLNKDVIMDMDDMVDEDPKWKAVDEAAINTCFDKIDAEISDSAKCKSGSYQLVRCMVRERFFNCPKDAWTESDECREYKMVVQRCTGLIPELYE

>HhalOBP4

MSPLAALLLAISVFGVVRAKDPVDCSKPPAGWPRQPPNCCDQPYPTDQMRKHLVGCIRQYGAPSSAVLTEKSVRERRSCVEECVYRSAGFINKEDSVLQREAIEKQLQSISGESWKNAISDSLNACFKEAEEIEDSLSASSSVEEESSCSSIPERLTFCLSRQLFLNCPEDTWKNSQECQVVKNRMEECKQLLPPPPIRFIRPGPRPPIVDQ

>HhalOBP5

MSYVLYLLLLSTFFSAQGLRCRMEQDEESQDEFEDIVRNCLREETVSPHHKENQDNRNKGYTRNNQQEKFNSSGGKREKEDNYGKDEEEYDYNEYDNDNSNNRDKNTQRRTYSQSTQKPQSNQYQGSKQTGARRNQGSRNIGSPLESIEPCIIHCIFKQKKMLNQNNKIDKSNTMHILTQKIRDSELKEFVEDSINECFDKMDNEKSEGKCEDSKKFALCLEEKGKNNCEDWDTNSNHTADNTNTFSNFNYNGPDESQSKRGERGNNRHQYETNNNSKDKMSGCFVRNERDEREGSKMKHESSNDKSSNNRKSSDSDHNEQKIKFSDYFQTRG

>HhalOBP6

MNSPVIVVALVLACTFSSYAMSPEFKAAIDECKTEHSIESGQIKEAVEHKKLPETENGRCFMSCVMEKMGVIKDGKIDQERVLEINKMKFKNPESLEKANEVAKRCANVEGTDERCSLATEMVKCAMENALELKLEMPEE

>HhalOBP7

MQAISSFIFFVVCLALAQGATVQEQVSAIAKKCAAENKVSAEQAKIAYAVVVPKTQEESCYLECIYTGAGVIKDGKFVVDGAKKLSAQRFTNAAEKTAADSVIDTCGKEVSAGKDKCALGKAVRECFVKNGKNISFFPPPS

>HhalOBP8

MERSLMILVFLMACALVEESSAAMSEAQMKGAMKTLRNMCLPKSGVSKEALANMKEGQFDDDDRKLKCYMGCIMNMMQVVKNGKISMTMVKNQIMKMVDPTWGAKLVATFESCASVEGSDNCDLAYNFGKCVYETDKEAFVVP

>HhalOBP9

MGSHCNFIYTICFVITFSVKARVLAAPTETEDCSVPIHLKNTDCCTSSSSVSLTNPVVNQECVKILSEAKASSYEEFESAVECFLECLFKTIHLLNGENKVIYEEILTNITQELKPDFANLEINKIKHCVEEDYTRKKSSQCKSGSLQFFLCVLREKILNCPPEVWKNSDECNKYKSVLENCPKRTPLFQIFDLEPDVKSNKTE

>HhalOBP10

MKRFSSQICAIVLLCGLGAQLVLALTEDECTLKPEEVPVCCKEPKAKVDAKEDKGREELGKKCVENNAKGKKATTDDEEFQIAECVDECIFRDVFGYVNKKTNKLDETAIVNTFTKRFDGNSKWKEATQKVAKSCLSESGKDIKASSKCKSGALQFLRCYTRGVFLNCPAESWDNTPACNKLKTLVQKCPNVYTILQN

>HhalOBP11

MKAIAVMVFALSSIAFVRAGVKEELKYQLEECRSTFNATDDEVKGIAVKQPPSSQEGKCYLHCILSRMDVMTEEGEMNSEGMKGVLREIPDLKESERKKMEQVADKCSRVSRLGDDRCENAVTIYNCINTESDELGVKG

>HhalOBP12

VADKCSREPEFCKPVDKRLYRPWLRLYKLYTPYSQQKYPKMNSLLFLTTILSAVCLSWAASDEYKMKVIASVAACAKEHGANIADVIEIVKAEKLPTNKAQKCVAGCFFHKMGYVTDSKVDWAKVKALNPKKIRK

>HhalOBP13

MQNHRVLFLLYALLAILLNESNGVEEQAECKDKSNLQVLGRCCNIPNEELLSEEEKAVGTMCDKTVYGDRITSPTTEEEQLELYECMDECFYNGTHLLTADMKLNETALIAEYDVSTENFSHWKEPITKAVKSCLEKNVVSEVKPDAKCKSGSYQFSQCFGLKLYLYCPPEDWKVKDEGCNKAREVLMNC

>HhalOBP14

MKSFFVVIALSALIALAKGDDVKEKFKKTAEKCKEKHAVTEEEIEKLKAKDTEFQYSHVAKCYMACFLEEGKILQNGKYNKENALVMTDVIHKENPEQAAKGKEIIETCAKQYPEVGEDQCEFAYQVSVCAAKEAKKVGLENTDFFAK

>HhalOBP15

AINDVLLQNLYKLPMAYVYTFAVVQVLLKMNIYIVNFIILTSISFTFASTVKPGIISQAFQIVGRCNKEHPLDMKEMEKAVKNFELPSSEEGKCFISCFLEGFGLVTDGQINLDRSLEFNKMQFHDPDNLEKANAISAACKDEMSTSTEKGCDFAIAASKCMLEKSKEMGFRFFQFHS

>HhalOBP16

MKHILGVGLMTVFLLLAGKARSSPSLGHTRNAMGGYNMNYDSEEEAASSYLGKDSIFKYSPQEETIAKILKSFGSCQAKHPLSADELTALKNQDTEMHFSHDAKCFFACMFKAADLLKNGRIIPENLIEYFNEDSIGVSNSREIIEYCNQEVSESEPDLCEYVYKMTKCWTKKILTS

>HhalOBP17

MNAALCLTTLLAAVCLSGAATPEYKAKVITSVTACAKEYNVELKDIIEIMKQSKLPETKEQKCVVGCFFEKMDYVTDHKVDWEKVKALNPQKYDTPELVEKINQVTDTCSKVVTEKSTDICELGVPAVKCLKEESEKVQLPKPEVKFDSA

>HhalOBP18

MNRINLLLLVVLVASAVGWPQPPPPPPPGGEDVPEECRPKPPPNPGKERAVCCDMPHPIAKDSDQFKTLFEECKEQIKSEHPEAMPPHHPHHPPPPPPPPAAGGPPPPPPPHHGGPHLFMTCMDECVFNKSGLLTDSKLNQEALTKMVDSFAPDGSWKTIALEATKFCYEKTSNETKTEDKDAKCSPVSHDFVKCMIKQMYLKCPSDKWQNNDDCNAEKQLIEKCPNLMPFPPHPPMKKKE

>HhalOBP19

MQPISSTIVFVVCLALAQGAAVQDQVTAIAKKCASEHKMSEEQAKIIFSLVVPKTPEENCYMECIYIGAGVMKDGKYSVEGAKKLADQRFTGADEKTAAVKLIETCKSVSAGSEKCGLAKSVRECFVKNGNNISFFPPPK

>HhalOBP20

MLSKGTVLVFAAINLMMVTAYREELKRTISGCQNGAEVTDDELEEFEKPLIPKNDEEKCLMACVFKSFGVIVDGTFDPKLALAVAKDLLKSDPEKVKKITAVVEHCGDDIPKKMDNECELAGEIMKCYVKWEKEVGLA

>HhalOBP21

MKYLVLCVVLSYAYAASVDTSKAILQKQFDVFRKCMVVGNVTESEVEVIFKKGEVPEGRKIKCLLGCFMKGMGYLTDDGKIDWSIMDDINRKEYVNPDEVKRALEISALCSKSVPQNLGNLCDAGYAGTKCFLDEAVKRKLTLHGPKDAE

>HhalOBP22

MFSSRMENKHFGRPRRTKPMFIVLLFLSSAYHSISFINAQPDDYSRKASSFKTNFMESSKYCSNIYNTSVFDVLPLFGQSDNGNLEAKCFLLCLLQRYQMIDKGGAFHRDKVDRFLDSMPDSKFKSSLKSFKEKCLKEVTDSQNQCQKAYKFSACFYNKGIDKKHES

>HhalOBP23

MMLVANMNRVVIVVALILACIFNCHGQANPEIKAAVNECRSQHNIEAGQIKDAINNKKIPETEHGQCFMSCVMKKMGVLKNGKIDLDRVSELINNKFKDQENREKAYEIAKRCANVKSPDGKECSQASEMAKCALKNAIELKMEVPKGSF

>HhalOBP24

MRSLVAVIVSFAIITSSLVLIQCQEEDIMEKKTEAITQCTAEHNVSKEAVKSISINDNVPNDPEVKCWLRCIFEKLGVMKEGQIDWERCKFITKHCLSNEQDKAKVDQITEICKAEVPKDEKDECQLAYSAMVCKMKNWKNFGLQ

>HhalOBP25

MKGITTALALTLVLATIEAEENVKAKIAKVFNTCKEKNPVTEDEIGAFRKADIGFSYSHDAKCMLACMLEEGKMLRDGKYMKENALIMADVFHIDDFDEAAKARKVIENCAIEVPEVGPDPCEFAFKMAVCGANESKKLGMKDHDFFE

>HhalOBP26

MKLFESILLTAIVIAVSVSLIGCHEDIVSIKKMAISRCSEIHSISKEAVKSIMNNEEVPNDYEIKCWLSCVLKTLGMLKDGKIMWETCRNITKYGFSEEDKAKVDKITEICQAEVPQEEKDECQLAYSAAVCKMNNWKKLGLPKGNWEE

>HhalOBP27

MNAVLCITTLLAAISVSGAMAPEYKKKLLTSLSDCMEKHNVDKEEIMQVIKNEKLPEDKEKQCLFGCLIDNMGYVKDLTLDWNLIKASNPEKFDRPEDVEKANTVTDTCAKTLSEKQPSLCELGVNAIKCIHQESLKIKLPKPEFSFD

>HhalOBP28

MTLEGKIDNMHLYLAGICSLLISLEIVSVANGAATPDECNDTGGENTGMCCKHKREPESKEKQEEYENNMKNCFSKYFGDIQDPPAEDVKISLMECVGECFFNASGLLTPDLKLSQEKLNAKDDIVDPDPKWKAIDEAAVKSCFDKSVAEGNGSAKCKSGSFQFLRCVLRERFLNCPKDVWTDGDECNNYRKVVEKCPNVIPGVA

>HhalOBP29

MVLIMSNYILALVLAVVSVDGSVGGLQGELSEKELLGLADMYHHSKAISKRSLRNTGDMDDHRYHKRHMFPDYKHCCGETDSRNNIRRKEAWTVVKDCVEQVKTKSNELEEFVNPITDEFSCERLKQEKNRHYCIADCVLKNTEALKDGTVNRDKAKEFFTKGISAEWLKEIAENAVEKCAEQKVFTSNGEQLDCSPQAVNLKHCFWKEMILTCPKEHFKKSPYCSRVKEAFEEMEN

>HhalOBP30

MKPLHVLCLLGAFAFASGVDIPDELKEMAQMVHDQCIGETGASADAIEGTKKGVFPADDQKLKCYLKCIYSNMGAISDEGELDAEAFGSIMPEELGRVLNPMINKCKDTTGPDGCELAFNFNICLYNADPKNYLVI

>HhalOBP31

MEFRSIIWTTLTILWSVHSLKLESLGHYIEGLYLAHDVQHPTGVIGARHVREVARTRHHQDYDMLFPFKYCCGGENNTVNNRKTVQSTRNVSMIVNRCFEERFEENDEWTTFGNPTKDPFSCENFKKAKDSFNCFFNCLMRGNGVLRDDGTEDLQVWEQITTETLAFPWLKDLAVTAFHKCVANKDYSWLQNGDKLECSPRVIDVNHCVWSQIILTCPEEHFVKNPYCKRVKVAFEDIDG

>HhalOBP32

MSSHNRLSSKGHFSEEHITQFFSHPQVTGTRHVREVAVSDNNNEGHKRKRPHRDGLLPYKHCCGGENNTEHSNHKEIKDIYLKCLDSELELKNEWSLHGNPTKDPFTCERMKKFKNHFYCIADCMMKNYGALGDDGSVDIDRWTEFTINDLAFPWLKDLATIAVEKCIANEDYSWLKNRGELECNPRAVDVNHCVWSQIMMTCPDEHFVKSSYCVRVKEAFEDVDG

>HhalOBP33

MMVQGAPEDYNECRNFVEKGLPDCCKRPEEGKRASAETVAIKKKCDQAVMGNRQMPPKSEDRMSIYECLDECYYNATKQFLDDLKPNEDAMIEAFLEGLESYPQWKDTVVKSVKNCVNSDYVSRLRKGAKCKSGVLELETCLGKQMYLNCPEDAWQSNRLECSNAKEIYIKCTPDI

>HhalOBP34

MVQKCLLVLTACILGVFGISSNENPLGFKIATFEECQAEGNVPELAMKELFYFKSAEGIKHHKCVMKCINEKYGIYQNNGTFNFENAVEAIKELFHDPVVQNTLINAGKECVKTVKENSDACDYAYDLVDCFIKETNKEGVAPHGEMPL

>HhalOBP35

NAVLCITTLLAAISVSVALGPVYKKRLLSSLTDCMERHRLNIDEVMDIVKNEKLPEDKEKQCFVGCTMNNLGYVKDLILDWNEVKKTNPEKFDTPEEVEKANTVADACAKTVSGKQPSLCELGVRAIKCMHQESQKIKLPIPDFSFD

>HhalOBP36

VSIRCSKNTYKLQFFNMFHLKLVLSLVLLTTYTVLSDEYEEIRAACEKEHNFDHKNMKFSDPNFPHEAKCALSCLFEKTDVFKKDGTIDREREKEMIQKVVKDEDLKKKFLKATDECDVSAKADKCETAYEFVKCKTEKTANMK

>HhalOBP37

MKNSLFLLAFLGIFVNNEAFGIGKEMAAMQCKAKFRVPYSDIEKIKNRQMPETKEGKCMMACILKKLKVISRDGKFQVNTVKGWIANKYKDDTKKLNRAYAKADACAEELPMLGEDECEYAVKILECSRRRKKLKVST

>HhalOBP38

MVSAASPIIFFLGLSVVLSLTPSEWDLAWQNAEAKCEHLMRIRTVDKKAFMTKLNDVPRRAKCFVSCFFDEVGLTNGTEINDALYTGWLQEELAHSKNKKAIISSVKQCVAGIKKIEKCQTAFSLYECFGKRYFT

>HhalOBP39

MLNLQIVLSLVLLTTHYALSETYEQIKAACESEHKINDLRAININDPAVPVEAKCALACMLEKMKVFQEDGSINKSEEKKIVEETVKEEDTKKMILMTIDECDGSAKATKCDTAFEYVKCKNNKLGTK

>HhalOBP40

MLSYHFVFFAVVLTVHSVWSQVFDEIAEACNKETGFHGDISKLHYTDPDIPKEAKCSLACALDKKGVFKADGTIDREKQKAIPQEIVKDEELKKKYLKAIDECDTSAKANKCETAYEFVKCLDTKVGE

>HhalOBP41

MYCSQLVFFAVLLTTHSVLSQKFDEIKEACNKEIGFDGDIETLHYNDPAFPKEAKCALACALDKKGVFKADGSIDREKEKAIPEEIIKDDELKKKYHKAIDECDASAKANKCETAYEFVKCLKNKVM

>HhalOBP42

MYCSYLFFVVGIFAVQSVQSVSFHDLISECKKNLAYDGDVHKLNYNDPAYPEKAKCVMACALEQKGVFKEDGSIDRVKEKAIPEEIIKDEEIKIKFFEAIDECDVSAKANKCETAHEFLKCIHNKV

>HhalOBP43

MHFSLSGILVVLAAHSVLSLDSFEEIKAVCNKETGYDGDIDKLNFNDPALPTKAKCAAACALEKQDLFKPDGSIDREKQKPLVEELVKDEDMKKSYMKAIEECDISAKANKCETAYEFLKCVDIKA

>HhalOBP44

EHKISEDAIKDIINHIQVNQDEEVKCWVSCLMKNLGIFKEGKIDWARFDEIVTSAVPKENVAKAHQIVQTCRSQVNQDEKNECQLAYSLADCKIKTW

>HhalCSP1

MRLIISCLLLIGLALAKPDTTKTDNKKEGYTTKYDNIDLEEILNNIRLLDKYFNCLIEKGKCTTDGKELKEIVPDALKTGCSKCNEKQKAGVEQVLRYLIEKKRDYFDELAKKYDPEGIYLKKYEAEAEKRGIKL

>HhalCSP2

MKVVLALLICTTVLAAKPGAHKGSYTTRYDNIDLEEILSNERLYKKYFDCLANKGKCTPDGKELKDVLPDALATECKKCSHKQQQGTEKVLRHLIEKRPVDYSVLEKLYDPTGTYKRKYKAEAEKRGIKIH

>HhalCSP3

MQSYTISALLFVCLAAAVSAATTYTTKYDNIDLDEILSNDRLYKKYFDCLISKGKCTADGKELKDILPDALATECKKCNEKQKAGAEKVLKFMLEKKAEDYAALEKIYDPEGAYKKKYAAEAEKRGIKIN

>HhalCSP4

MFIAAALAILLSATLTPSFGDDQEDIYKKIFDDVDIDGILNNDRILDTYLRCFFNSGPCSNLADTIRGKIPEVFSTVCGLCTEKQKGLFKHSLDIFIPKRPDDWKHILEIYDPDGSYWPKIKEFLETY

>HhalCSP5

MLLLIGLLAVRQMFGEAAVSDTQLERQVLARLQVIDVERILNNNRIINKYLKCMLRQGVCPPEARDFRRVLPKLIKHLCEKCTDRQRTALKQIFTFVRTKFPKEWEQMKILYATNPEDQIRMEKFAAT

>HhalCSP6

MRVILAVILFAGVALARPDGYTTKYDNINLDEILNNDRLYQKYFQCLTNKGKCTPDGKQLKDILPDALKSKCAKCNERQRKGAEKVFKYLLDKKPNDYKVLEKIYDPQGVYRAQYKSEAEKRGIKI

>HhalCSP7

MRVILACLLFVGLVFAKPDGYTTKYDNIDLEEILHNERLYKKYYDCLANKGTCTPDGKELKDIVPDALKTGCKKCNEKQKKGVEKVLKYVLENKRADYDYLEKIYDPQGIYRKKYQAEAEKHGIKI

>HhalCSP8

MLRTLLLLAPLVLACFCQAAATQQSYTDKWDRIDVDQILKNDRILKKYVDCLMDRGKCSPDAQELRKVLPEAIQTECAKCTDSQKRMAGKALSYILQNKRNYWNELIGKYDPKGEFRKKYEYEEDK

>HhalCSP9

MKLVLLLVFVIGAAFAAEVYTSKYDNIDVDKILSNDRILSQYIKCLMEEGNCTNEGRELKKTLPDALATGCAKCSEKQKAQTEKVLRHLTKNRPRDWARLKTKYDPTGEYSKKYEPKATTAAPAAA

>HhalCSP10

HDSLSARINHYVGMNPTLVFVVIGVVMTGILCAESRPSVSDEALETALKDRRYLTRQLKCALGEGPCDPVGRRLKTYAPLVLRGTCPKCSPQEVRQIQQVLSHIQRNYPKEWSKILKQYAGQ

>HhalCSP11

MKAVLALALFAAVAVARPGDKYTTKYDNIDLDEILNNERLYKRYFNCLANRGKCTPDGKELKAVLPDALETECAKCSDRQKKGSDKVLKFLLEKKAADYDVLEKIYDPTGIYRKKFTMKKE

>HhalCSP12

MKVVIALLLFAAVAIAKPADMYTTKYDNIDIDEILNNERLYKKYIDCVMDKGDCTPDGKELRKNIPDAIATDCAKCSEHQKTGTDKVLNYMLKNKKADYDELEKKFDPKGEYRKRHNIKA

>HhalCSP13

MKLVILLLVVLAAVASADKYTTQYDNIDIDEILSNDRLYKKYYDCLMGKGKCTPDGQELKKNMPDAITTDCSKCSEKQKVGSQKVLKFMLDKKESDYTNLEKVFDPTGTYRKKHAQS

>HhalCSP14

VIVIGLFILTILAPAAPLELLPPKVDGHDVTQILTDDKLFQQYFDCVMGRTKCTPGGQIVKDGIPAQLKDGCANCPPIRRIGAQIIVRFMIATRCPQYEEFEKKYDPQQKLRKLYAA

>HhalCSP15

MMLVLSLLMVSVAAALPADTYTTKYDNLDVGEILKNDRLYQKYNECLSNTGTCTPDGKELKDILGEIIKTDCKKCSEKQKKNIVKFLKQILEEKPEDFVKLEKIYDPDQVFRKKYAS

>HhalCSP16

MSSKAPSPLCDTVAMRRTSFLVAVCMLFSLSAVLADEEDIDVYEKILEDFDVDTIINNDRLLDSYLKCFFNTGPCSDIAEMVKGKIPEVFSTVCGLCTEKQKGLFKHSLDIFIPKRP

>HhalCSP17

MRFTVALFAFVVFAAAAPQSAKIDKYDIEQIFNDEKLFQQYFNCVMGRGQCTPGGQKLKDSIADHVKSGCANCPPERKARAQKMVKFMIAKKAPQYEEFEKMYDPEQKLRKLYA

>HhalSNMP1.1

MAAPLRLGVAGAVLSLVGIIFGFWGFHKFLAYKINQNVALKKGNDMRAAWSKFPIPVEFKIFLFNVTNPQEVHTGQKPKLQEIGPYFFDEWKEKVKLEDDPAEDTVSFNQRTAWIFQESRSEGLTGEEMITIPHPALLSMVLTVEKQKPGALPMISKALPALFNSPSTVFLTAKAMDILFRGVPINCSSNDFGPKAICTMIRANPKGLKKLNDDIFLFSFFGEKNYTIDEGRFTVKRGVRDAKEVGTVVKFNGKEIQDVWSGPECNALRGTDSTIFPPFIDDSEDIVSFAPDLCRSLGAKFKHKIVYKGIPGNHYTADLGDMSSNPEEKCFCPTPDTCLKKGAFDISKCVGAPIVLTLPHFYETDPSYLATVEGLHPDKEKHQIFLNFEPMTGTPLGARKRLQFNIPIHPIKKVALMKELPDALVPLFWVEEGLELDQKFIDLLDSQLFRALRIVGVSKWVIIVLGLAMIGGGVGLHYYRKNKMNSPTVTQVSPPPNKF

>HhalSNMP1.2

SPQYFHCSEYFIRQSRYPPLQSVLSMSAKKAQFSDVVLERIKAVPQKLKEAPPKKYGKVGAALVLGGVGFGWLIFPYILSFAVGKIVTLDDGSDVRKIWKKIPQYLDFNIYLFNITNPMEVQNGGKPILQEVGPYRYIEWKEKVNLVDDIGEDTITYSNLNTWYFLKEKSLPLTGDEMVTIPHLPLLSVLLVAEQDFPTPMLTIVNAAVPHIYGKLGSVFMTAKVRELLFDGVLIDCTAKNIVPKAICIAIKQNSKALVKLGKNKYLFSFFGIRNATPEDVRITVKKGVQDVYSIGKVVAMNGNTENVVWSGGECRRFSGTDSTIFPPFRKPDNYSIVAFSPEICRTMSGSYVGEGAYQGVRGYRYVVSLGDMKRNPGEMCFCPSPDRCLGKGTTDLTKCQGAPLIGSLPHFYDAEEEYLNGVVGMKPDKDKHEITFIMEPISGVPLLARKRLQFNIHLHPVRFVNLTKKLTPTLVPIFWLEENLDLGDELMGFLEANLLTNLRLVDVVKWMLIVVGAGVCVAGVVLYRMKKEANKKTNSREDLLPPGNDDVEVLDPSRLSGSTASLVRSLVQPTAPVTHTAWDVVPSPSLQLPAIETIGASPGISSSAPLEPRARSTAEALHSSLSTGRLRKF

>HhalSNMP2

MKGLMELRGLSRVVYTGSAGCFIIVLSIYFGIAGFPYLLQQQIKSKIVLNNGSEGMEAWESLPLPLEFKVFIFNVTNPDEVSKGMQPVVQELGPYVYDQYRRKVNIEFTEDDTISYRIEKKFYFNKNKSGCYRESDVVVVPNVPLIGTAYRVEERFPMGLIFINSSASLLFPGIKNLFLTTTVGDLFFNGVRIKCDYLKGPAMPVCQGIKRNLPPSLKEIPLSRDFAFSYFSDANSSVSGVFKTYRGNKNVYDLGRIIKYDNNTHLTMWDKNTTCSELKGTDSTILAPIQNKDQDIYIFLPEVCLSLKAVFSRATNMYGIDVYQYMASHHNFDSEKRNPSHICRCKKQEDEPNAPPMCLKDGAIDASKCQGAPVVFTYPHMLFADPEYQNYVKGYKGDYEKHQTEVFIEPRTGVPLAAFKRIQMNIFLRRLEDVDLFANISEGLFPLIWIEEALTEELVQTYLSDMKELMSTTRIIMSVTGLLIGVGIFCLLVALILYIKHRNVACMKENQVVSNISLIGHGIVTRKDPDTAEKRIAYDLPNSLSSDRLTVQKF
